# Knee osteotomy: Quality tools and readability data of information on the internet

**DOI:** 10.1016/j.dib.2020.106624

**Published:** 2020-12-08

**Authors:** James M. Broderick, Andrea McCarthy, Niall Hogan

**Affiliations:** Department of Trauma & Orthopaedics, St. James's Hospital, James's Street, Dublin D08 NHY1, Ireland

**Keywords:** Knee osteotomy, Patient education, Internet, Quality of information, Readability

## Abstract

Supplementary data for the article **Osteotomy around the Knee: Assessment of Quality, Content and Readability of Online Information** is provided. 45 unique websites were evaluated. The DISCERN score, JAMA (Journal of the American Medical Association) benchmark criteria and HONcode (Health On the Net) criteria are provided for reference. Readability of online information was analysed with Readability Studio Professional Edition, Version 2019 (Oleander Software Ltd.). The software assessed readability using eight different instruments: Flesch-Kincaid Reading Grade Level (FKGL), Flesch Reading Ease Score (FRES), Raygor Estimate, SMOG, Coleman- Liau, Fry, FORCAST and Gunning Fog. Data is also provided on the percentage of complex words, long words, Dale-Chall unfamiliar words, Fog words, as well as the number of ‘wordy’ items, overly long sentences and longest sentence length.

## Specifications Table

**Table:** 

Subject	Health and medical sciences
Specific subject area	Osteotomy around the knee
Type of data	Appendix (1) Table (3) Figure (1) Raw Data Document (1)
How data were acquired	Primary data was acquired by searching four Internet search engines (Google, Yahoo!, Bing and Ask) with the search terms “*high tibial osteotomy*” and “*knee osteotomy*.” Each website's text was converted into a Microsoft Word document (Microsoft, Redmond, WA). All hyperlinks, pictures, advertisements, copyright notices, and any other text that was not directly related to health information was subsequently removed. Secondary readability data was then acquired by analyzing the re-formated documents with Readability Studio Professional Edition, Version 2019 (Oleander Software Ltd.).
Data format	Raw Filtered
Parameters for data collection	Online information related to osteotomy around the knee.
Description of data collection	Readability of online information related to osteotomy around the knee was analysed with Readability Studio Professional Edition, Version 2019 (Oleander Software Ltd.).
Data source location	Institution: St. James's University Hospital City: Dublin Country: Ireland Primary Data Source: The list of unique websites evaluated is provided in Table 1.
Data accessibility	With the article
Related research article	Broderick J.M., McCarthy A., Hogan N. Osteotomy around the Knee: Assessment of Quality, Content and Readability of Online Information. The Knee.

## Value of the Data

• Readability, a metric that determines the ease with which text can be read and understood, is a major factor affecting the ability of patients to utilize health information. Readability of information is a key component of health literacy.
• Researchers involved with examining the quality and readability of health information, as well as those tasked with generating patient educational materials, will benefit from this supplemental material. Tools are provided to assess the quality and readability of healthcare information. The ‘wordy’ items list will act as a repository for commonly encountered complex words or phrases, with suggested alternatives, which content creators will be able to cross-reference against.
• Future studies will be required to assess the progress made in providing accurate, high quality and readily comprehensible healthcare information. The supplemental material provided here may be used in designing such studies, and furthermore, will provide baseline readability data against which future studies may compare their findings.

## 1 Data Description

**Appendix A**. The DISCERN Instrument, JAMA Benchmark Criteria and HONcode Criteria

A.1. The DISCERN Instrument

The DISCERN Instrument was developed by an expert group in the United Kingdom to allow consumers and information providers assess the quality of written health information. The DISCERN Handbook ensures all users are able to understand and apply the instrument effectively [1]. 16 questions address clarity, balance, and content of information with each question representing a separate quality criterion. The original tool has 16 questions, each rated on a 5-point scale (1 = definite ‘no’; 2–4 = ‘partially meets criteria’; 5 = definite ‘yes’). The first eight questions relate to the reliability of the publication and seven questions address specific details of the information about treatment choices. The final question is an overall quality rating. Similar to Weil et al. [2]., we omitted the final question to obtain a minimum score of 15 and a maximum score of 75. Websites were then classified by their total score as ‘excellent’ (63–75), ‘good’ (51–62), ‘fair’ (39–50), ‘poor’ (27–38) or ‘very poor’ (15–26).

A.2.TheJournal of the American Medical Association (JAMA) Benchmark Criteria

The Journal of the American Medical Association (JAMA) benchmark criteria, originally published by Silberg et al. [3], represent four core standards to determine whether a source of information is credible, reasonable or useful: authorship, attribution, currency and disclosure. Authorship requires that the authors and contributors provide their affiliations and credentials. Attribution entails listing of references and sources for all content and indicating any relevant copyright information. Currency refers to the provision of the dates that content was posted and updated. Disclosure ensures that websites have properly demonstrated ‘ownership’ with any sponsorship, advertising, underwriting, commercial funding arrangements or potential conflicts of interest noted. One point is allocated for each core criterion that is met, with a maximum score of 4.

A.3. The HONcode Criteria

The Health on the Net Foundation is a non-profit, non-governmental organization, which seeks to establish ethical standards for publishing medical and health-related information on the internet. The Health On the Net code (HONcode) seal accredits websites that agree to comply with eight core standards (the HONcode Criteria [4]) and publish transparent health related information.

An Internet search provided the primary data source for assessment. Four search engines (Google, Yahoo!, Bing and Ask) were searched for the terms “*high tibial osteotomy*” and “*knee osteotomy*.” Forty websites were analyzed from Google and ten each from Yahoo!, Bing and Ask. Following exclusion criteria and removal of duplicated websites, forty five unique websites were available for analysis.

Readability of a text is defined as “the determination by systematic formulae of the reading comprehension level a person must have to understand written materials [5].” The scales used in our study include seven reading grade level (RGL) scores (the Flesch-Kincaid Reading Grade Level [FKGL], Raygor Estimate, SMOG, Coleman- Liau, Fry, FORCAST and Gunning Fog) and one index score (the Flesch Reading Ease Score [FRES]). RGL is reported as a United States grade level, which denotes the years of education (based on the U.S. educational system) required to easily read and understand a piece of text. For each website evaluated, the seven RGL tests generated seven RGL scores, as well as a mean RGL. The FRES formula presents readability as an index score, based on sentence length and number of syllables. The score ranges from 0 to 100, with a higher score indicating easier readability.

‘Wordy’ items are either complex words or phrases that contain too many words and therefore require more advanced reading skills. A complete list of all ‘wordy’ items encountered is provided, along with suggested alternatives to improve text readability.

A summary of important linguistic units that influence readability scores is presented (Fig. 2). This provides an ‘at a glance’ overview of key elements analyzed by the readability formulae.

**Readability Raw Data Document.**

Raw data from the readability analysis of the 45 unique websites is provided.

This secondary data is provided as:

• *Readability Scores*
  ○ Raw scores of each of the 8 readability formulae for each website.
  ○ Summary scores of each readability formula for all 45 websites.
  ○ Grade score summary for each website.
  ○ Cloze score summary for each website.
• *Histogram representation of scores for each readability formula for all 45 websites*
  ○ Coleman-Liau
  ○ New Dall-Chall
  ○ Flesch Kincaid
  ○ FRES
  ○ FORCAST
  ○ Fry
  ○ Gunning-Fog
  ○ Raygor-Estimate
  ○ SMOG
• *Box Plot representation of scores for reading grade level tests*
• *Words Breakdown*
  ○ Difficult Words
    ■ % of complex works
    ■ % of long sentences
    ■ % of SMOG Hard words
    ■ % of FOG Hard words
    ■ % of Dale-Chall Unfamiliar words

**Fig. 1:**
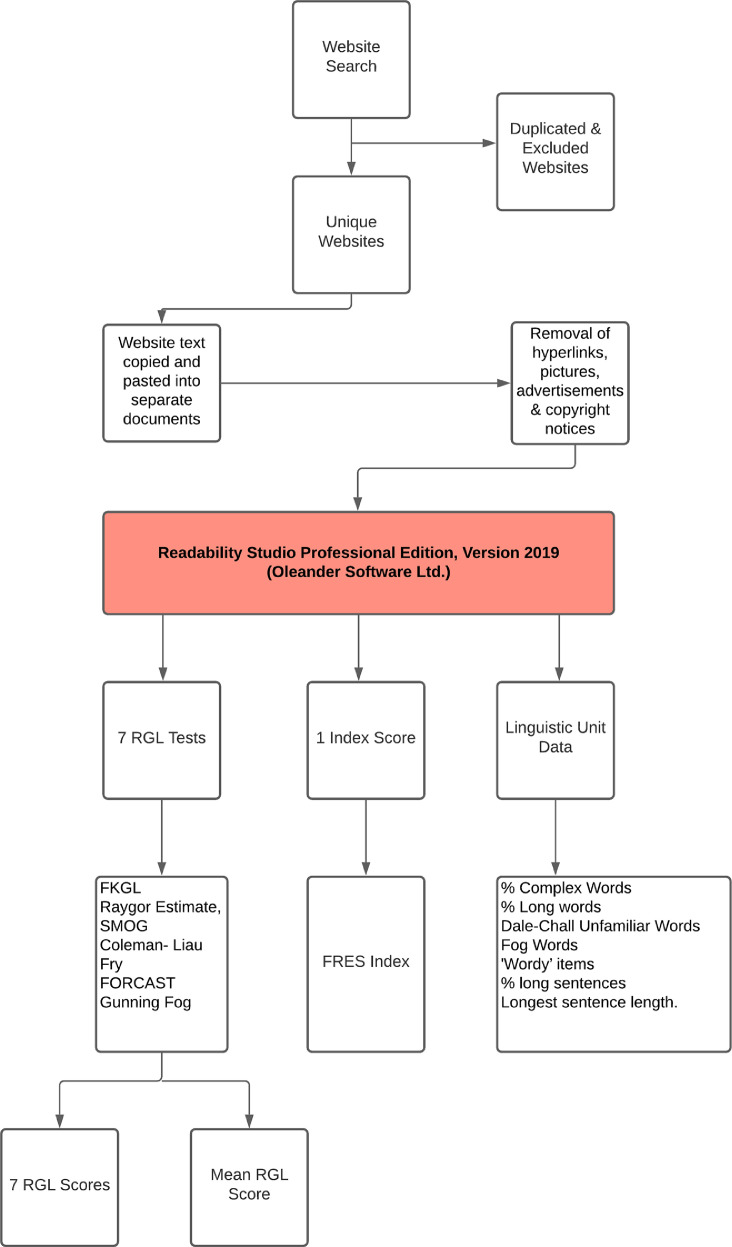
Study design.
  ○ All Words
    ■ Individual word count
    ■ Number of websites word appears in
• *Sentences Breakdown*
  ○ Long Sentences by website
    ■ Number of overly long sentences
    ■ Longest sentence length
    ■ Longest sentence
• *Wordy Items*
  ○ Presented by website

## 2 Experimental Design, Materials and Methods

Four search engines (Google, Yahoo!, Bing and Ask) were searched for the terms “*high tibial osteotomy*” and “*knee osteotomy*.” To reflect the disproportionate use of the search engines [6], we analysed the first 40 websites from Google and 10 each from Yahoo!, Bing and Ask. Searches were performed on April 08, 2020. Websites were excluded from further analysis if duplicate findings were noted between search engines, if the site was inaccessible, if the site was solely for advertisement purposes or if it was in video format. A total of 45 unique websites were analysed (Table 1).

**Table 1:** List of unique websites evaluated.

Search Engine		Website
Google	1	https://www.hss.edu/conditions_knee-surgery-high-tibial-osteotomy.asp
	2	https://orthoinfo.aaos.org/en/treatment/osteotomy-of-the-knee
	3	https://www.ncbi.nlm.nih.gov/pmc/articles/PMC3374001/
	4	https://www.orthobullets.com/recon/3135/high-tibial-osteotomy
	5	https://www.arthritis-health.com/surgery/knee-surgery/knee-osteotomy-surgery
	6	https://www.mayoclinic.org/tests-procedures/knee-osteotomy/about/pac-20394514
	7	https://josr-online.biomedcentral.com/articles/10.1186/s13018–019–1333–4
	8	https://www.hindawi.com/journals/jhe/2019/8363128/
	9	https://tocamd.com/knee-re-alignment-osteotomy/
	10	https://www.hindawi.com/journals/crior/2018/2493095/
	11	https://www.mihaivioreanu.ie/
	12	https://www.sciencedirect.com/topics/medicine-and-dentistry/high-tibial-osteotomy
	13	http://rebalancemd.com/wp-content/uploads/2017/08/HTO_Recovery_Guide.pdf
	14	http://brochures.mater.org.au/brochures/mater-hospital-brisbane/high-tibial-osteotomy
	15	https://en.wikipedia.org/wiki/High_tibial_osteotomy
	16	http://www.pinehurstsurgical.com/wp-content/uploads/2016/05/High-Tibial-Osteotomy.pdf
	17	https://jeo-esska.springeropen.com/articles/10.1186/s40634–019–0177–5
	18	https://www.raleighsportsmed.com/high-tibial-osteotomy-dr-barker-orthopedic-surgeon-cary-garner-nc.html
	19	http://www.e-aosm.org/journal/download_pdf.php?doi=10.14517/aosm14009
	20	http://fowlerkennedy.com/wp-content/uploads/2015/11/HIGH-TIBIAL-OSTEOTOMY-HTO-PROTOCOL-November-2015.pdf
	21	https://www.aspetar.com/journal/viewarticle.aspx?id=393#.XpAwfv1KjIU
	22	https://www.ftlauderdaleortho.com/high-tibial-osteotomy.html
	23	https://journals.lww.com/jbjsjournal/Fulltext/2019/06050/Does_High_Tibial_Osteotomy_Still_Have_a_Role_in.16.aspx
	24	http://aoj.amegroups.com/article/view/3720/4378
	25	https://www.multnomahortho.com/knee-osteotomy-orthopedic-surgeon-portland-beaverton-gresham-oregon.html
	26	https://www.healio.com/orthopedics/knee/news/print/orthopedics-today/%7B6fc8a303-d686–4ad7-abb8–6474f5fde606%7D/
	27	https://www.orthopedicsurgeonnyc.com/hto-high-tibial-osteotomy-treatment
	28	https://blog.peekmed.com/high-tibial-osteotomy-guide/
	29	https://www.arlingtonortho.com/conditions/knee/knee-tibial-osteotomy-with-open-wedge/
	30	https://www.newyorkortho.com/high-tibial-osteotomy.html
	31	https://www.brighamandwomens.org/assets/BWH/patients-and-families/rehabilitation-services/pdfs/knee-high-tibial-osteotomy-bwh.pdf
	32	https://www.tandfonline.com/doi/full/10.3109/17453674.2012.688725
	33	http://drhipandknee.com/high-tibial-osteotomy-procedure-for-knee-arthritis/
	34	https://www.researchgate.net/publication/330282444
	35	https://medcraveonline.com/MOJOR/high-tibial-osteotomy-in-patients-with-stages-2-and-3-of-knee-osteoarthritis-short-term-result-and-factors-affecting-the-outcome.html
	36	https://www.ismoc.net/high-tibial-osteotomy-il.html
Yahoo!	37	https://www.oakneepain.co.uk/treatment/high-tibial-osteotomy
	38	https://www.nuffieldhealth.com/treatments/tibial-osteotomy
	39	https://www.kneeguru.co.uk/KNEEnotes/courses/realignment-osteotomy-knee-pain/high-tibial-osteotomy-and-distal-femoral-osteotomy
	40	https://www.cumbriankneeclinic.co.uk/hto-high-tibial-osteotomy.php
	41	https://www.bonsecours.com/health-care-services/orthopedics-sports-medicine/knee/treatments/knee-osteotomy
Bing	42	https://www.ncbi.nlm.nih.gov/pmc/articles/PMC4999379/
Ask	43	https://www.guysandstthomas.nhs.uk/resources/patient-information/therapies/physiotherapy/Physiotherapy-following-high-tibial-osteotomy-surgery.pdf
	44	http://www.newcastle-hospitals.org.uk/services/musculoskeletal_treatment-and-medication_high-tibial-osteotomy.aspx
	45	https://www.ncbi.nlm.nih.gov/pmc/articles/PMC4969364/

All text from the articles was copied and pasted into separate Microsoft Word documents (Microsoft, Redmond, WA). All hyperlinks, pictures, advertisements, copyright notices, and any other text that was not directly related to health information were removed. Readability of the re-formated documents was then analyzed with Readability Studio Professional Edition, Version 2019 (Oleander Software Ltd.). All reading grade levels (RGL) were reported as a United States grade level, which denotes the years of education (based on the U.S. educational system) required to easily read and understand a piece of text. We selected eight different instruments to assess readability (Table 2). These included seven RGL tests (Flesch-Kincaid Reading Grade Level [FKGL], Raygor Estimate, SMOG, Coleman- Liau, Fry, FORCAST and Gunning Fog) and one Index Score (the Flesch Reading Ease Score [FRES]). For each website, the seven RGL tests generated seven RGL scores, as well as a mean RGL. The FRES formula calculates the readability of a document, expressed as an index score, based on sentence length and number of syllables. The score ranges from 0 to 100, with higher scores indicating an easier readability. All websites were also analyzed for the percentage of complex words, long words, Dale-Chall unfamiliar words, Fog words, as well as the number of ‘wordy’ items, overly long sentences and longest sentence length. Complex words are defined as words with ≥ 3 syllables and long words as those with ≥ 6 characters. Dale-Chall unfamiliar words are defined as those that do not appear on a list of 3000 common words that are known to most 4th-grade students. Fog words are words that contain ≥ 3 syllables that are not proper nouns, combinations of easy or hyphenated words, or two-syllable verbs made into three by adding -es and -ed endings. ‘Wordy’ items include complex words and phrases that contain too many words. Overly long sentences are defined as those with a word count greater than 22 words.

**Table 2:** Summary of readability formulae.

Readability Test	Score Type	Description	Formula
Flesch- Kincaid Reading Grade Level	Grade Level	Part of the Kincaid Navy Personnel collection of tests. Designed for technical documents and suited to a broad array of disciplines.	*G* = (11.8 X (B/W)) + (0.39 X (W/S)) −15.59
Flesch Reading Ease Score	Index Score (0–100)	Developed to assess the readability of newspapers. Best suited to assessing school textbooks and technical manuals. Standard test used by many US government agencies. Scores range from 0 to 100, with higher scores denoting easier readability.	*I* = (206.835 - (84.6 X (B/W)) - (1.015 X (W/S)))
The Raygor Estimate	Grade Level	Designed for most text, including literature and technical documents	Calculated using the mean number of sentences and long words (≥6 characters) per 100 words, which are plotted on to a RE Graph, where their intersection determines RGL.
Fry	Grade Level	Designed for a variety of texts including technical documents and literature, across a range of levels, from primary school level to university level.	Calculated using the mean number of sentences and syllables per 100 words, which are plotted on to a Fry Graph, where their intersection determines RGL.
SMOG	Grade Level	Generally appropriate for secondary age (4th grade to college level) readers. Tests for 100% comprehension, whereas most formulas test for around 50%−75% comprehension. Most accurate when applied to documents ≥30 sentences in length.	*G* = 1.0430 X √*C* + 3.1291
Coleman-Liau	Grade Level	Designed for secondary age (4th grade to college level) readers. Formula is based on text from the 0.4 to 16.3 grade level range. Applicable to numerous sectors.	*G* = (−27.4004 X (E/100)) + 23.06395
FORCAST	Grade Level	Devised for assessing U.S. Army technical manuals and forms. It is the only test not designed for running narrative.	*G* = 20-(M/10)
Gunning Fog	Grade Level	Developed to assist American businesses improve the readability of their writing. Applicable to numerous disciplines.	*G* = 0.4 X (W/*S*+((C*/W) X 100))

*G*=Grade level; *B*= Number of syllables; *W*= Number of words; *S*= Number of sentences; RGL= Reading Grade Level; *I*= Flesch Index Score; RE= Raygor Estimate; SMOG= Simple Measure of Gobbledygook; *C*= Complex words (≥3 syllables); *E* =predicted Cloze percentage=141.8401 - (0.214590 X number of characters) + (1.079812*S); *M*= Number of monosyllabic words; C*= Complex words with exceptions including, proper nouns, words made 3 syllables by addition of "ed" or "es", compound words made of simpler words.

**Table 3:** ‘Wordy’ items with suggested alternatives produced by readability studio software.

Wordy Items	Suggestion
ability	skill
absolutely	wholly
absolutely essential	essential
abundant	enough
accelerate	hasten, quicken
acceptable	welcome
accompanied	went with
accompanying	going with
accomplished	did, done
accordingly	so, just so
accuracy	correctness, exactness
accurate	correct, exact
accurately	correctly, exactly
a certain amount of	some, much
achievable	doable, makeable
achieve	do, make
achieved	did, made
achieves	does, makes
achieving	doing, making
acquired	gained, got
actual	real
actually	really
adapt	make fit
adapted	made fit
additional	added, extra
additionally	added, more
adequate	enough
adhere	stick to, follow
adjacent	next to
adjacent to	close to, near, next to, beside, by
adjustment	settlement
adjustments	settlements
administered	managed
advantage	plus
advantageous	helpful
advantages	pluses
adverse	harmful
advise	tell, recommend
advised	told, recommended
advocated	spoke for
aggressive	forward, strong, attacking
a great deal of	much, vast
a large number of	numerous, many
alleviate	make easier
alleviated	made easier
alleviates	makes easier
alleviating	making easier
all of	all (unless proceeding a pronoun)
almost all	most
alteration	change
alterations	changes
alternate	take turns (between), every other (adj.)
alternative	choice
alternatives	choices
amendments	changes
an alternative	any other, another
analysis	review, breakdown, exam, study
and also	and, also
anterior	front
anticipated	expected, awaited
a number of	a few
anxiety	fear
apex	tip
apparent	clear, plain
appear	seem, come
appeared	seemed, came
appears	seems, comes
appropriate	proper (adj.), set aside (verb)
appropriately	properly
approval	praise, consent
approximately	about
are prone to	tend to
as a result	so, then, thus
as a result of	because of, due to, following
ascending	climbing, upward
as long as	if, since
as of now	about
as opposed to	compared to
assist	aid, help
assistance	help
assisted	aided, helped
assuming that	if
as to whether	whether
as well as	and, also
at about	about
at all times	always
at present	now, today
attempt	try
attempted	tried
attempts	tries
at the time	when
at this time	now, right now
attractive	pleasing
augmentation	increase
available	offered, ready
a wide range of	assorted, extensive, numerous
beneficial	helpful
benefit	help
benefits	helps
bilateral	two-sided
both of	both (unless proceeding a pronoun)
by means of	by, with, from, in, over, through
capacity	ability, power, position
cartilage	gristle
categories	classes, groups
category	class, group
certainly	surely
cessation	stop, pause
characteristics	traits
characterize	describe
characterized	described
clarify	make clear
collection	mass, heap
combined	joined
combining	joining
commenced	began
commences	begins
commitment	pledge
compensate	pay
component	part
components	parts
composed	made up, created, calm (adj.)
compress	squeeze
compresses	squeezes
comprise	form, include
comprised	formed, included
concerning	about, on
conclude	close, end
concluded	closed, ended
conclusion	close, end
conclusive	final
congenital	inborn
consequently	so
consolidation	combination, merger
consolidations	combinations, mergers
constitute	be, form
constituted	was, formed
constitutes	makes up, forms
construct	build
construction	building
constructs	builds
containing	having, holding
contains	has, holds
contemplate	think about
contemplating	thinking about
continue	keep, keep on
continued	kept on
continue on	continue
continues	keeps on
continuing	keeping on
contribute	give, help
contributed	gave, helped
contributes	gives, helps
contributing	giving, helping
contribution	gift
convenient	handy
conversion	change
conversions	changes
create	make
created	made
creates	makes
creating	making
criteria	requirements
debilitating	weakening
definitive	final
demonstrate	show
demonstrated	showed
demonstrates	shows
density	thickness
descending	downward
desired	wished
despite the fact that	although, even though, despite
determine	decide, figure
determined	decided, figured
determines	decides, figures
determining	deciding, figuring
detrimental	harmful
develop	make, grow
developed	made, grown
developing	making, growing
develops	makes, grows
deviates	strays, turns away
deviation	change
difficult	hard
difficulties	troubles
difficulty	trouble
discovered	found out
disrupt	interrupt, confuse
disrupted	interrupted, confused
disrupting	interrupting, confusing
due to the fact that	because, since, given that
eccentric	odd
elect	choose, pick
electing	choosing, picking
elevate	raise, lift up
elevated	rose, lifted up
elevates	raises, lifts up
elevating	raising, lifting up
elevation	height
elicit	draw out, call forth
eliminate	cut, drop
eliminated	cut, dropped
eliminating	cutting, dropping
eminence	high place
employment	work, job, use
encountered	met
encourage	urge
encouraged	urged
encourages	urges
encouraging	urging
endeavours	tries, attempts
ensure	make sure
ensured	made sure
ensures	makes sure
equines	horses
equivalent	equal
established	set up, proved
evaluate	check, rate
evaluated	checked, rated
evaluating	checking, rating
evaluation	check, rating
evaluations	checks, ratings
evidenced	showed
evidences	shows
evident	clear
examination	check
examine	check, look at
examined	checked, looked at
except when	unless
exchange	trade
exchanged	traded
exchanging	trading
explain	show, tell
explained	showed, told
explaining	showing, telling
explains	shows, tells
external	outer
facilitate	ease, help
facilitates	eases, helps
familiar	known
feasible	can be done
final	last
first introduced	introduced
for the sake of	for
frame of mind	attitude, posture, view, viewpoint
frequently	often
from start to finish	completely, thoroughly
function	act, role
fundamental	basic
general	broad
general consensus	consensus
generally	broadly
generate	create, make
generating	creating, making
gives rise to	causes, leads to, results in
has a tendency	tends to
has no	lacks
has the ability to	can
have an influence on	affect, influence
have a tendency	tend to
have no	lacks
have the ability to	can
hazardous	risky, unsafe
heterogeneous	varied
high degree of	abundant, ample
horizontally	sideways
however	but
identical	same
identical to	the same as
identification	ID
identified	named, found
identifies	names, finds
identify	name, find
identifying	naming, finding
illustrates	draws, shows
immediately	at once, right away, right now
imminent	near
impact	hit, change
impair	harm, weaken, reduce
impaired	harmed, weakened, reduced
imperative	urgent
implementation	carrying out
in advance of	ahead of, before, by
in an effort to	to
in a similar fashion	like
inasmuch as	because, since, as, as far as
in association with	along with, as well as
in cases where	where
in certain cases	at times, sometimes
incision	cut
incisions	cuts
in close proximity to	close to, near
incomplete	partial
in conjunction with	along with, and, combined with, coupled with, joined with, paired with, with
in contrast to	compared to
inconvenience	bother
incorporated	blended, joined, mixed
indicate	show
indicated	shown
indicates	shows
indicating	showing
indication	clue, sign
indications	clues, signs
individual	person, single
individuals	people
individuals who	those who
inferior	lesser
inhibition	restraint
in isolation	along
initial	first
initially	at first
initiate	begin
initiated	started
in most cases	mostly, most of these, often, usually
in order for	for
in order to	to, for
in other words	that is
inquiries	questions
in some cases	at times, sometimes
in spite of	aside, despite, although
institution	office, company, school
insufficient	not enough
in terms of	as for
internal	inner, inside
in the absence of	without
in the case of	in, with, if, by, for
in the context of	in, about, for, of
in the presence of	with, before
in the right	correct, right, justified
in the sense that	in that
investigate	review, check, look over
investigated	reviewed, checked, looked over
investigating	reviewing, checking, looking over
is able to	can
is composed of	comprises
is comprised of	comprises
is consistent with	coheres to, conforms with
is defined as	is
is dependent on	depends on, hinges on
is in line with	conforms with
it is important that	must, should
it is important to note	note
it is probable that	probably
it should be noted	note
just about	about
known as	called, named
limitation	limit
limitations	limits
limited number of	a few, little, meager, not many, scant, only so many, some, spare, sparse
located	found
locates	finds
locating	finding
location	place
maintain	keep, support
maintained	kept, supported
maintaining	keeping, supporting
maintains	keeps, supports
majority	most
manufactured	made
manufactures	makes
massive	large
maximum	most, greatest
methodologies	methods, designs, plans
methodology	method, design, plan
meticulous	very careful
minimize	decrease, lessen
minimised	decreased, lessened
minimize	decrease, lessen
minimized	decreased, lessened
modification	change
modifications	changes
modified	changed
modify	change
modifying	changing
monitor	check, watch
monitored	checked, watched
monitoring	checking, watching
multiple	many
necessarily	needed, needed to
necessary	needed
necessitate	cause, need
necessitates	causes, needs
needs to have	needs
need to have	need
never ever	never
no matter how	however
no more than	only
notify	let know, tell
not many	few
not possible	impossible
numerous	many
objective	aim, goal
objectives	aims, goals
oblique	slanting
observed	saw, seen
observing	seeing
obtain	get
obtained	got
obtaining	getting
obtains	gets
occurrence	event
occurrences	events
of major importance	is important, are important, was important
on a regular basis	regularly
on the basis of	by, from, because of, assuming, based on, from
on the contrary	rather, instead
on the other hand	however
on the side of	with
on the surface	seemingly, apparently
operate	run, work
operated	ran, worked
operating	running, working
opt for	choose
option	choice, way
options	choices, ways
other similar	similar
outside of	outside (unless proceeding a pronoun)
over and over again	repeatedly
over the course of	during, throughout
participate	take part
participated	took part
participating	taking part
particular	specific
perform	do
performed	did/done
performing	doing
permit	let
permitted	let
physician	doctor
physicians	doctors
point of view	opinion
portion	part
portions	parts
position	place
positioned	placed
positioning	placing
possess	have, own
posterior	rear
predominant	superior
predominantly	superiorly
preferable	better
preparation	readiness
previous	earlier, past
previously	before, earlier
primarily	mainly, firstly
primary	main, first
prior to	before
probability	chance
procedure	rule, way, method
procedures	rules, ways, methods
proceed	do, go on
produced	made
produces	makes
producing	making
proficient	expert, skilled
profound	deep, thoughtful
program	plan
propagated	bred, reproduced
propensity	inclination, tendency
purchased	bought
rapid	quick
rapidly	quickly
reason why	reason
reciprocating	giving in return
recommend	suggest
recommended	suggested
recommends	suggests
reduce	cut
reduced	cut
reduces	cuts
reducing	cutting
reduction	cut
regimen	routine, rule
reimburse	pay back
reinforce	strengthen
remain	stay
remained	stayed
remaining	staying, left over
remains	stays
request	ask
require	need
required	needed
requirement	need
requirements	needs
requires	needs
requiring	needing
resulted in	lead to
result in	lead to
resulting in	leading to
results in	leads to
retain	keep, hold
retained	kept, held
retention	keeping, holding
reversion	return
review	check
reviewed	checked
reviewing	checking
rigidity	stiffness
satisfied	happy, content
scrutinized	inspected, examined
segment	part
segments	parts
similar	like
simultaneously	at the same time
so as to	to
solicited	asked for
sooner or later	eventually
speculate	reflect, guess, surmise, suppose
strategy	plan
submit	send, give
subsequent	later, next
subsequently	later, after
substantial	real, strong, large
substantially	really, strongly, largely
sufficient	enough, ample
sufficiently	amply
suitability	fitness
superior	better, boss
take into consideration	consider
taken into account	considered
taken into consideration	considered
tertiary	third
the fact that	that
the majority of	most, most of
the other way around	the opposite
the reason for	because, since, why
therefore	so, thus
thereof	its, their
the sum of	all
to a certain extent	in a sense, somewhat, partly
took into account	considered
transmits	sends
transmitted	sent
typically	often
uncommonly	rarely
up until	until
usually	often
utilization	use
utilize	use
utilized	used
utilizing	using
validate	confirm
value	cost, worth
variation	change, difference
variations	changes, differences
via	in, on, by
viable	workable
visualize	picture
visualized	pictured
visualizes	pictures
was comprised of	comprised
whenever	when
whereas	since
with regard to	about, regarding
write down	write

‘Wordy’ items are complex words and phrases that contain too many words.

**Fig. 2:**
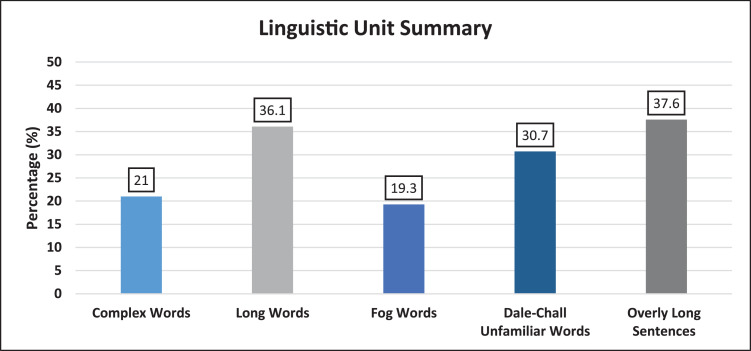
Linguistic unit summary of all articles Complex words are words with ≥ 3 syllables; Long words are words with ≥ 6 characters; Fog Words are words that contain ≥ 3 syllables that are not proper nouns, combinations of easy or hyphenated words, or two-syllable verbs made into three by adding -es and -ed endings; Dale-Chall unfamiliar words are words that do not appear on a list of 3000 common words that are known to most 4th-grade students; Overly long sentences are defined as those with a word count greater than 22 words.

Experimental design is outlined in Fig. 1.

## Ethics statement

This study analyzed the educational material on publicly accessible websites and as such was exempt from IRB review.

## CRediT Author Statement

James M. Broderick conceived and designed the study, participated in data acquisition, performed the statistical analysis and drafted the manuscript. Andrea McCarthy performed a literature review and participated in data acquisition. Niall Hogan participated in study design. All authors read and approved the final manuscript.

## Declaration of Competing Interest

The authors declare that they have no known competing financial interests or personal relationships which have or could be perceived to have influenced the work reported in this article.

## Appendix B Supplementary materials

Image, application 1

Image, application 2
