# Supplementary material for: Knee osteotomy: Quality tools and readability data of information on the internet
Source: Data Brief. 2020 Dec 8;34:106624. doi: 10.1016/j.dib.2020.106624 (PMC7744941; doi:10.1016/j.dib.2020.106624)
Supplement: Supplementary file 1 [file mmc1.docx]

**Appendix A**. The DISCERN Instrument, JAMA Benchmark Criteria and HONcode Criteria

**A.1. The DISCERN Instrument**

| Items of the Reliable Score |
| --- |
| 1. Are the aims clear? |
| 1. Does it achieve its aims? |
| 1. Is it relevant? |
| 1. Is it clear what sources of information were used to compile the publications (other than the author or producer)? |
| 1. Is it clear when the information used or reported in the publication was produced? |
| 1. Is it balanced and unbiased? |
| 1. Does it provide details of additional sources of support and information? |
| 1. Does it refer to areas of uncertainty? |
| Items of the Treatment Score |
| 1. Does it describe how each treatment works? |
| 1. Does it describe the benefit of each treatment? |
| 1. Does it describe the risks of each treatment? |
| 1. Does it describe what would happen if no treatment was used? |
| 1. Does it describe how treatment choices affect overall quality of life? |
| 1. Is it clear there may be more than 1 possible treatment choice? |
| 1. Does it provide information for shared decision-making? |
| Overall Impression of Website |
| 1. Based on answers to all of the above questions, rate the overall quality of the publication as a source of information about treatment choices. |

This questionnaire includes the 16 questions of the DISCERN instrument extracted from [www.discern.org.uk](http://www.discern.org.uk) [1]

**A.2. The Journal of the American Medical Association (JAMA) Benchmark Criteria [3]**

| Core Standard | Description |
| --- | --- |
| Authorship | Authors and contributors, their affiliations, and relevant credentials should be provided |
| Attribution | References and sources for all content should be listed clearly, and all relevant copyright information noted |
| Disclosure | Website “ownership” should be prominently and fully disclosed, as should any sponsorship, advertising, underwriting, commercial funding arrangements or support, or potential conflicts of interest. This includes arrangements in which links to other sites are posted as a result of financial considerations. Similar standards should hold in discussion forums |
| Currency | Dates that content was posted and updated should be indicated |

JAMA, Journal of the American Medical Association

**A.3.** ^†^**The HONcode Criteria[4]**

| 1. **Authoritative** | Any medical advice provided and hosted on this site will only be given by medically trained and qualified professionals unless a clear statement is made that a piece of advice offered is from a non-medically qualified individual/organisation. |
| --- | --- |
| 1. **Complementarity** | The information provided on this site is designed to support, not replace, the relationship that exists between a patient/site visitor and his/her existing physician. |
| 1. **Privacy** | Confidentiality of data relating to individual patients and visitors to a medical Website, including their identity, is respected by this Website. The Website owners undertake to honour or exceed the legal requirements of medical information privacy that apply in the country and state where the Website and mirror sites are located. |
| 1. **Attribution** | Where appropriate, information contained on this site will be supported by clear references to source data and, where possible, have specific HTML links to that data. The date when a clinical page was last modified will be clearly displayed. |
| 1. **Justifiability** | Any claims relating to the benefits/performance of a specific treatment, commercial product or service will be supported by appropriate, balanced evidence in the manner outlined in principle (4). |
| 1. **Transparency** | The designers of this Website will seek to provide information in the clearest possible manner and provide contact addresses for visitors that seek further information or support. The web-master will display his/her e-mail address clearly throughout the Website. |
| 1. **Financial Disclosure** | Support for this Website will be clearly identified, including the identities of commercial and non-commercial organisations that have contributed funding, services or material for the site. |
| 1. **Advertising Policy** | If advertising is a source of funding it will be clearly stated. A brief description of the advertising policy adopted by the Website owners will be displayed on the site. Advertising and other promotional material will be presented to viewers in a manner and context that  facilitates differentiation between it and the original material created by the institution operating the site. |

HON, Health On the Net

^†^Reprinted, with permission, from: Boyer C, Selby M, Scherrer JR, Appel RD. The Health On the Net Code of Conduct for medical and health websites. Comput Biol Med. 1998 Sep; 28(5):603-10

[1] The DISCERN Handbook. Quality criteria for consumer health information on treatment choices. Available from: https://wwwdiscernorguk 1998.

[3] Silberg WM, Lundberg GD, Musacchio RA. Assessing, controlling, and assuring the quality of medical information on the Internet: Caveant lector et viewor--Let the reader and viewer beware. JAMA 1997;277(15):1244-5. https://www.ncbi.nlm.nih.gov/pubmed/9103351.

[4] Boyer C, Selby M, Scherrer JR, Appel RD. The Health On the Net Code of Conduct for medical and health Websites. Comput Biol Med 1998;28(5):603-10. https://doi.org/10.1016/s0010-4825(98)00037-7.
