# Supplementary material for: Knee osteotomy: Quality tools and readability data of information on the internet
Source: Data Brief. 2020 Dec 8;34:106624. doi: 10.1016/j.dib.2020.106624 (PMC7744941; doi:10.1016/j.dib.2020.106624)
Supplement: Supplementary file 2 [file mmc2.docx]

**Readability Data of Online Knee Osteotomy Information**

| **PROJECT TITLE**: | Readability Data of Online Knee Osteotomy Information |
| --- | --- |
| **DATE**: | 28/10/2020 |
|  |  |
|  |  |

- Readability Scores
- Histograms
- Box Plots
- Words Breakdown
- Sentences Breakdown
- Wordy Items

**Readability Scores**

**Raw Scores**

| Website No. | Coleman-Liau (grade levels) | Coleman-Liau (pred. Cloze scores) | New Dale-Chall | Flesch-Kincaid | Flesch Reading Ease | FORCAST | Fry | Gunning Fog | Raygor Estimate | SMOG |
| --- | --- | --- | --- | --- | --- | --- | --- | --- | --- | --- |
| 1 | 12.7 | 38 | 13-15 | 14 | 37 | 11.4 | 15 | 17.1 | 13 | 15.6 |
| 2 | 10.2 | 47 | 9-10 | 9.9 | 55 | 10 | 11 | 12.8 | 9 | 12.5 |
| 3 | 14.5 | 31 | 16+ | 16.5 | 24 | 11.8 | 17 | 19+ | 17 | 18 |
| 4 | 13.1 | 36 | 16+ | 11.7 | 39 | 11.8 | 16 | 15.4 | 17 | 14.6 |
| 5 | 11.3 | 43 | 11-12 | 10.8 | 50 | 10.7 | 12 | 13.4 | 11 | 13.3 |
| 6 | 10 | 48 | 9-10 | 9.9 | 57 | 9.9 | 10 | 12.5 | 9 | 12.6 |
| 7 | 14.9 | 30 | 16+ | 19 | 19 | 11.8 | 17 | 18.5 | 17 | 19+ |
| 8 | 15.2 | 29 | 16+ | 15.6 | 26 | 12 | 17 | 18.2 | 17 | 17.1 |
| 9 | 10.8 | 45 | 9-10 | 10.2 | 55 | 10.2 | 11 | 13 | 10 | 12.8 |
| 10 | 14.8 | 30 | 13-15 | 14.6 | 28 | 11.7 | 17 | 16.8 | 17 | 16.1 |
| 11 | 12.2 | 40 | 11-12 | 11.5 | 44 | 11 | 15 | 13.9 | 12 | 13.9 |
| 12 | 12.8 | 38 | 13-15 | 12.2 | 41 | 11.1 | 17 | 14.7 | 13 | 14.4 |
| 13 | 9.7 | 49 | 9-10 | 8.7 | 62 | 10.1 | 9 | 10.2 | 9 | 11.4 |
| 14 | 9.7 | 49 | 7-8 | 8.3 | 62 | 10.1 | 9 | 11 | 8 | 11.3 |
| 15 | 15.1 | 29 | 16+ | 16.2 | 23 | 11.6 | 17 | 19+ | 17 | 17.6 |
| 16 | 11.5 | 42 | 13-15 | 13.1 | 43 | 10.6 | 13 | 16.4 | 12 | 15.2 |
| 17 | 15.9 | 26 | 16+ | 18.1 | 16 | 12.2 | 17 | 19+ | 17 | 18.9 |
| 18 | 12.3 | 39 | 13-15 | 13.6 | 41 | 10.4 | 14 | 16.5 | 12 | 15.3 |
| 19 | 13.3 | 36 | 16+ | 14.7 | 32 | 11.2 | 17 | 17.5 | 17 | 16.6 |
| 20 | 15.8 | 27 | 13-15 | 12.5 | 35 | 12 | 17 | 12.2 | Text is too difficult to be classified to a specific grade level because it contains too many 6+ character words. | 14.1 |
| 21 | 15 | 30 | 16+ | 17 | 18 | 11.7 | 17 | 19+ | 17 | 18.3 |
| 22 | 12.1 | 40 | 13-15 | 12.6 | 41 | 10.8 | 14 | 16.2 | 12 | 15.1 |
| 23 | 14.6 | 31 | 16+ | 17.4 | 19 | 12 | 17 | 19+ | 17 | 18.7 |
| 24 | 15 | 30 | 16+ | 19+ | 14 | 11.9 | 17 | 19+ | 17 | 19+ |
| 25 | 11.7 | 42 | 11-12 | 13.1 | 42 | 10.8 | 13 | 16.1 | 12 | 15 |
| 26 | 15 | 29 | 16+ | 16.1 | 24 | 11.7 | 17 | 19 | 17 | 17.4 |
| 27 | 13.3 | 36 | 13-15 | 13 | 37 | 11.6 | 16 | 14.9 | 17 | 15.1 |
| 28 | 10.5 | 46 | 11-12 | 10.2 | 53 | 10.5 | 11 | 12.7 | 10 | 12.9 |
| 29 | 9.2 | 50 | 9-10 | 9.8 | 57 | 9.6 | 10 | 12.4 | 7 | 12.1 |
| 30 | 11.7 | 41 | 11-12 | 11.2 | 48 | 10.5 | 12 | 14.6 | 11 | 13.7 |
| 31 | 13.6 | 34 | 13-15 | 12.4 | 38 | 11.5 | 17 | 14.4 | 17 | 14.3 |
| 32 | 11.5 | 42 | 11-12 | 15.6 | 43 | 10.4 | 17 | 14.2 | 12 | 17.1 |
| 33 | 10.3 | 47 | 11-12 | 10.3 | 54 | 10.1 | 11 | 13.5 | 9 | 13.3 |
| 34 | 15.3 | 28 | 16+ | 17.3 | 21 | 12.1 | 17 | 17.7 | 17 | 17.9 |
| 35 | 12.2 | 40 | 13-15 | 15.4 | 38 | 10.5 | 17 | 15.3 | 12 | 16.1 |
| 36 | 11.4 | 42 | 11-12 | 9.7 | 51 | 11.1 | 11 | 12.7 | 11 | 12.5 |
| 37 | 10.2 | 47 | 11-12 | 11 | 53 | 10.1 | 11 | 13.6 | 10 | 13.1 |
| 38 | 8.9 | 52 | 7-8 | 8.5 | 61 | 9.9 | 9 | 10.7 | 7 | 11.2 |
| 39 | 9.6 | 49 | 11-12 | 12.5 | 52 | 9.7 | 11 | 14 | 10 | 14.1 |
| 40 | 10.5 | 46 | 11-12 | 11.2 | 53 | 10.1 | 11 | 14.2 | 10 | 13.3 |
| 41 | 9.3 | 50 | 9-10 | 8.7 | 58 | 9.9 | 10 | 11.7 | 8 | 11.8 |
| 42 | 14 | 33 | 16+ | 14.2 | 31 | 11.4 | 17 | 16.9 | 17 | 16 |
| 43 | 9.2 | 51 | 5-6 | 8 | 65 | 9.5 | 8 | 10.7 | 7 | 11.3 |
| 44 | 8.9 | 52 | 7-8 | 8.3 | 64 | 9.5 | 9 | 11.3 | 7 | 11.5 |
| 45 | 13.8 | 34 | 16+ | 16.1 | 27 | 11.7 | 17 | 18.2 | 17 | 17.4 |

**Score Summary**

| Test | Valid N | Minimum | Maximum | Range | Mode(s) | Means |
| --- | --- | --- | --- | --- | --- | --- |
| Coleman-Liau (grade levels) | 45 | 8.9 | 15.9 | 7 | 15 | 12.3 |
| Coleman-Liau (pred. Cloze scores) | 45 | 26 | 52 | 26 | 30; 42 | 39 |
| Flesch-Kincaid | 45 | 8 | 19 | 11 | 8 | 12.9 |
| Flesch Reading Ease | 45 | 14 | 65 | 51 | 41; 53 | 41 |
| FORCAST | 45 | 9.5 | 12.2 | 2.7 | 11 | 10.9 |
| Fry | 45 | 8 | 17 | 9 | 17 | 14 |
| Gunning Fog | 45 | 10.2 | 19 | 8.8 | 14; 19 | 15.1 |
| New Dale-Chall | 45 | 5.5 | 16 | 10.5 | 16 | 12.8 |
| Raygor Estimate | 44 | 7 | 17 | 10 | 17 | 13 |
| SMOG | 45 | 11.2 | 19 | 7.8 | 11; 12; 13; 15; 17 | 14.9 |

**Grade Score Summary (x Website)**

| Website No. | Valid N | Minimum | Maximum | Range | Mode(s) | Means |
| --- | --- | --- | --- | --- | --- | --- |
| 1 | 8 | 11.4 | 17.1 | 5.7 | 14; 15 | 14.1 |
| 2 | 8 | 9 | 12.8 | 3.8 | 9 | 10.6 |
| 3 | 8 | 11.8 | 19 | 7.2 | 16; 17 | 16.2 |
| 4 | 8 | 11.7 | 17 | 5.3 | 11; 16 | 14.5 |
| 5 | 8 | 10.7 | 13.4 | 2.7 | 11 | 11.8 |
| 6 | 8 | 9 | 12.6 | 3.6 | 9 | 10.4 |
| 7 | 8 | 11.8 | 19 | 7.2 | 17; 19 | 16.7 |
| 8 | 8 | 12 | 18.2 | 6.2 | 17 | 16 |
| 9 | 8 | 9.5 | 13 | 3.5 | 10 | 10.9 |
| 10 | 8 | 11.7 | 17 | 5.3 | 14 | 15.3 |
| 11 | 8 | 11 | 15 | 4 | 11 | 12.6 |
| 12 | 8 | 11.1 | 17 | 5.9 | 14 | 13.7 |
| 13 | 8 | 8.7 | 11.4 | 2.7 | 9 | 9.7 |
| 14 | 8 | 7.5 | 11.3 | 3.8 | 8; 9; 11 | 9.4 |
| 15 | 8 | 11.6 | 19 | 7.4 | 17 | 16.2 |
| 16 | 8 | 10.6 | 16.4 | 5.8 | 13 | 13.2 |
| 17 | 8 | 12.2 | 19 | 6.8 | 17; 18 | 16.8 |
| 18 | 8 | 10.4 | 16.5 | 6.1 | 12; 14 | 13.5 |
| 19 | 8 | 11.2 | 17.5 | 6.3 | 17 | 15.4 |
| 20 | 7 | 12 | 17 | 5 | 12 | 13.9 |
| 21 | 8 | 11.7 | 19 | 7.3 | 17 | 16.4 |
| 22 | 8 | 10.8 | 16.2 | 5.4 | 12 | 13.4 |
| 23 | 8 | 12 | 19 | 7 | 17 | 16.5 |
| 24 | 8 | 11.9 | 19 | 7.1 | 19 | 16.7 |
| 25 | 8 | 10.8 | 16.1 | 5.3 | 11; 13 | 12.9 |
| 26 | 8 | 11.7 | 19 | 7.3 | 17 | 16.2 |
| 27 | 8 | 11.6 | 17 | 5.4 | 13; 14 | 14.4 |
| 28 | 8 | 10 | 12.9 | 2.9 | 10 | 11.2 |
| 29 | 8 | 7 | 12.4 | 5.4 | 9 | 10 |
| 30 | 8 | 10.5 | 14.6 | 4.1 | 11 | 12 |
| 31 | 8 | 11.5 | 17 | 5.5 | 14 | 14.3 |
| 32 | 8 | 10.4 | 17.1 | 6.7 | 11; 17 | 13.7 |
| 33 | 8 | 9 | 13.5 | 4.5 | 10 | 11.1 |
| 34 | 8 | 12.1 | 17.9 | 5.8 | 17 | 16.3 |
| 35 | 8 | 10.5 | 17 | 6.5 | 12; 15 | 14.1 |
| 36 | 8 | 9.7 | 12.7 | 3 | 11 | 11.4 |
| 37 | 8 | 10 | 13.6 | 3.6 | 10; 11 | 11.3 |
| 38 | 8 | 7 | 11.2 | 4.2 | 7; 8; 9 | 9.1 |
| 39 | 8 | 9.6 | 14.1 | 4.5 | 9; 11; 14 | 11.6 |
| 40 | 8 | 10 | 14.2 | 4.2 | 10; 11 | 11.5 |
| 41 | 8 | 8 | 11.8 | 3.8 | 9 | 9.9 |
| 42 | 8 | 11.4 | 17 | 5.6 | 16 | 15.3 |
| 43 | 8 | 5.5 | 11.3 | 5.8 | 8; 9 | 8.7 |
| 44 | 8 | 7 | 11.5 | 4.5 | 7; 8; 9; 11 | 9.1 |
| 45 | 8 | 11.7 | 18.2 | 6.5 | 17 | 15.9 |

**Cloze Score Summary (x Website)**

| Website No. | Valid N | Minimum | Maximum | Range | Mode(s) | Means |
| --- | --- | --- | --- | --- | --- | --- |
| 1 | 1 | 38 | 38 | 0 | 38 | 38 |
| 2 | 1 | 47 | 47 | 0 | 47 | 47 |
| 3 | 1 | 31 | 31 | 0 | 31 | 31 |
| 4 | 1 | 36 | 36 | 0 | 36 | 36 |
| 5 | 1 | 43 | 43 | 0 | 43 | 43 |
| 6 | 1 | 48 | 48 | 0 | 48 | 48 |
| 7 | 1 | 30 | 30 | 0 | 30 | 30 |
| 8 | 1 | 29 | 29 | 0 | 29 | 29 |
| 9 | 1 | 45 | 45 | 0 | 45 | 45 |
| 10 | 1 | 30 | 30 | 0 | 30 | 30 |
| 11 | 1 | 40 | 40 | 0 | 40 | 40 |
| 12 | 1 | 38 | 38 | 0 | 38 | 38 |
| 13 | 1 | 49 | 49 | 0 | 49 | 49 |
| 14 | 1 | 49 | 49 | 0 | 49 | 49 |
| 15 | 1 | 29 | 29 | 0 | 29 | 29 |
| 16 | 1 | 42 | 42 | 0 | 42 | 42 |
| 17 | 1 | 26 | 26 | 0 | 26 | 26 |
| 18 | 1 | 39 | 39 | 0 | 39 | 39 |
| 19 | 1 | 36 | 36 | 0 | 36 | 36 |
| 20 | 1 | 27 | 27 | 0 | 27 | 27 |
| 21 | 1 | 30 | 30 | 0 | 30 | 30 |
| 22 | 1 | 40 | 40 | 0 | 40 | 40 |
| 23 | 1 | 31 | 31 | 0 | 31 | 31 |
| 24 | 1 | 30 | 30 | 0 | 30 | 30 |
| 25 | 1 | 42 | 42 | 0 | 42 | 42 |
| 26 | 1 | 29 | 29 | 0 | 29 | 29 |
| 27 | 1 | 36 | 36 | 0 | 36 | 36 |
| 28 | 1 | 46 | 46 | 0 | 46 | 46 |
| 29 | 1 | 50 | 50 | 0 | 50 | 50 |
| 30 | 1 | 41 | 41 | 0 | 41 | 41 |
| 31 | 1 | 34 | 34 | 0 | 34 | 34 |
| 32 | 1 | 42 | 42 | 0 | 42 | 42 |
| 33 | 1 | 47 | 47 | 0 | 47 | 47 |
| 34 | 1 | 28 | 28 | 0 | 28 | 28 |
| 35 | 1 | 40 | 40 | 0 | 40 | 40 |
| 36 | 1 | 42 | 42 | 0 | 42 | 42 |
| 37 | 1 | 47 | 47 | 0 | 47 | 47 |
| 38 | 1 | 52 | 52 | 0 | 52 | 52 |
| 39 | 1 | 49 | 49 | 0 | 49 | 49 |
| 40 | 1 | 46 | 46 | 0 | 46 | 46 |
| 41 | 1 | 50 | 50 | 0 | 50 | 50 |
| 42 | 1 | 33 | 33 | 0 | 33 | 33 |
| 43 | 1 | 51 | 51 | 0 | 51 | 51 |
| 44 | 1 | 52 | 52 | 0 | 52 | 52 |
| 45 | 1 | 34 | 34 | 0 | 34 | 34 |

**Histograms**

**Coleman-Liau (grade levels)**


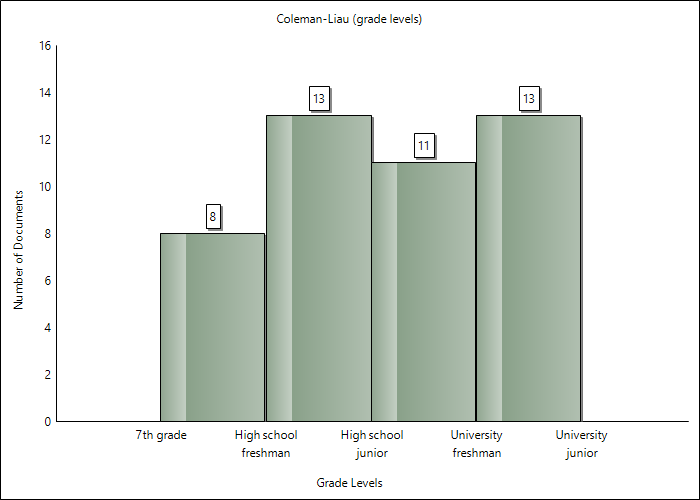


**New Dale-Chall**


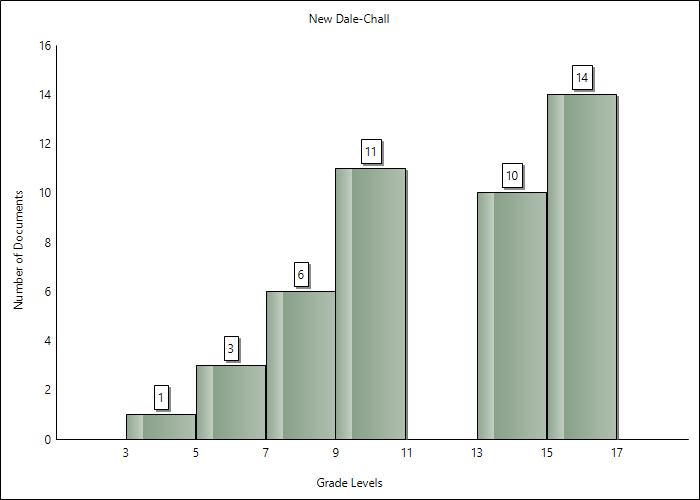


**Flesch-Kincaid**


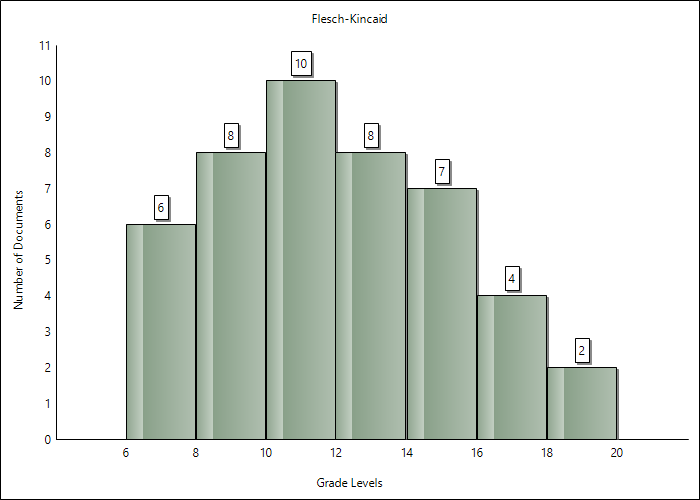


**Flesch Reading Ease**


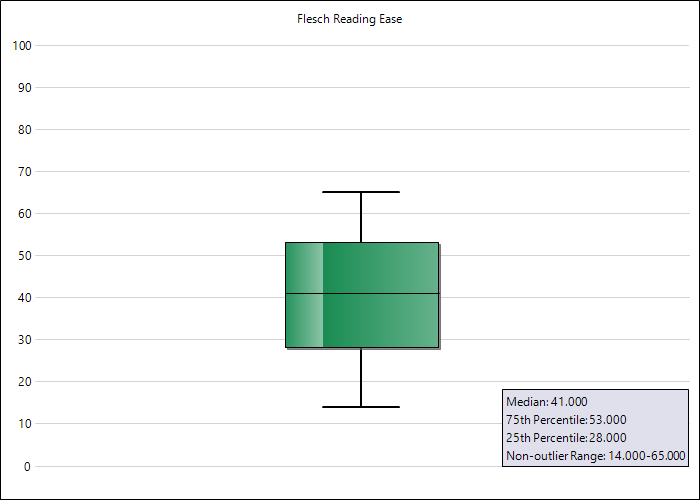


**FORCAST**


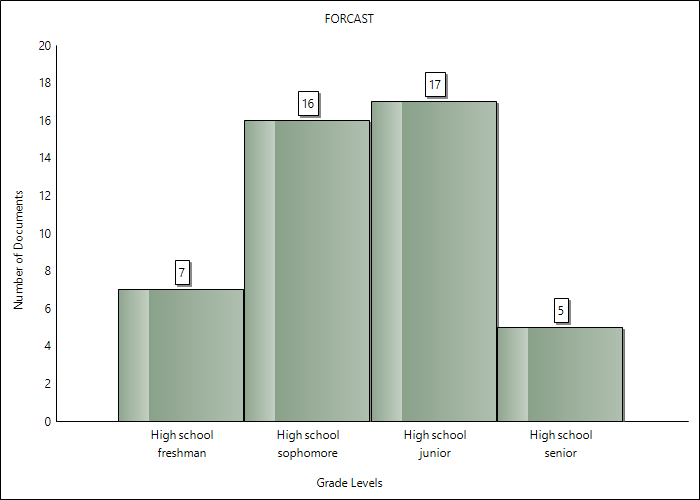


**Fry**


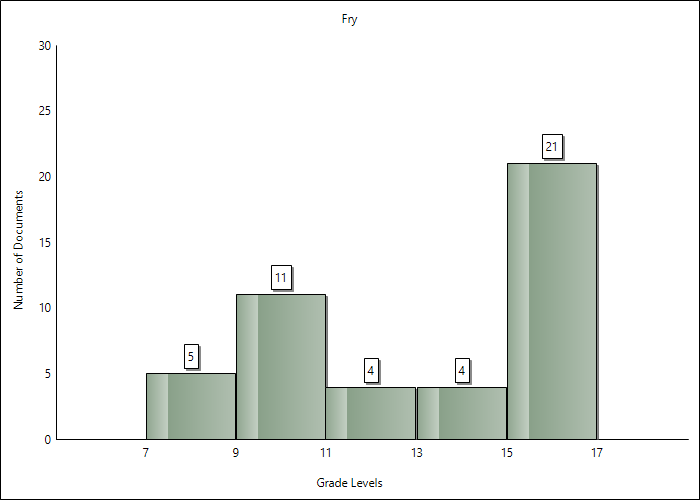


**Gunning Fog**


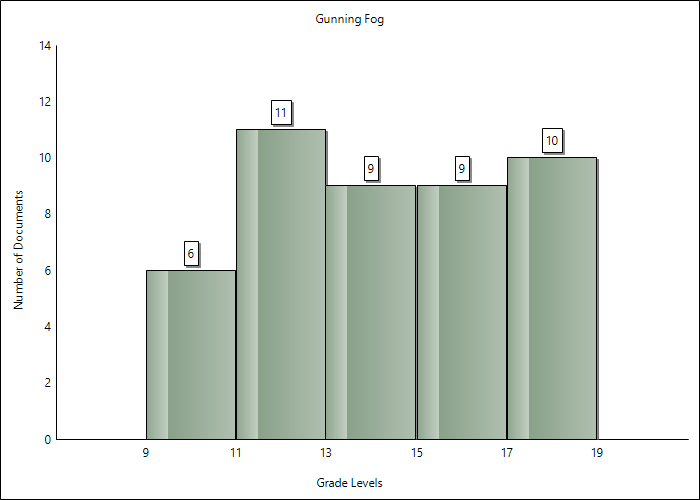


**Raygor Estimate**


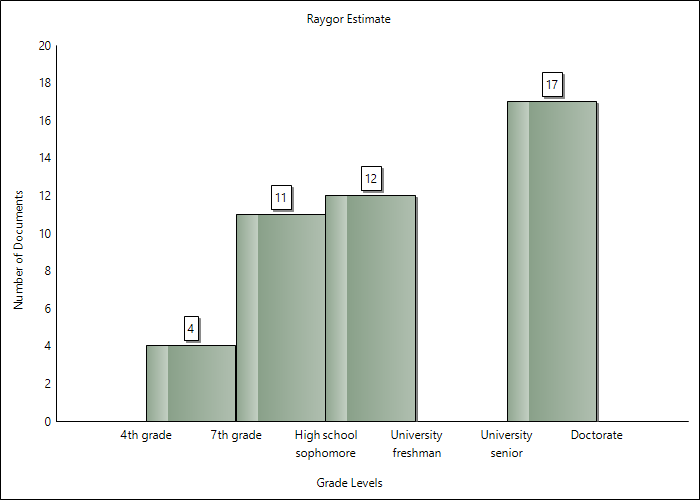


**SMOG**


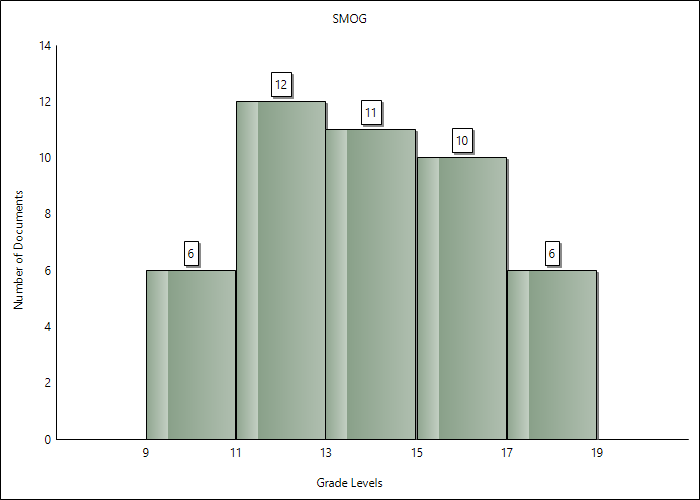


**Box Plots**

**Grade-level Tests**


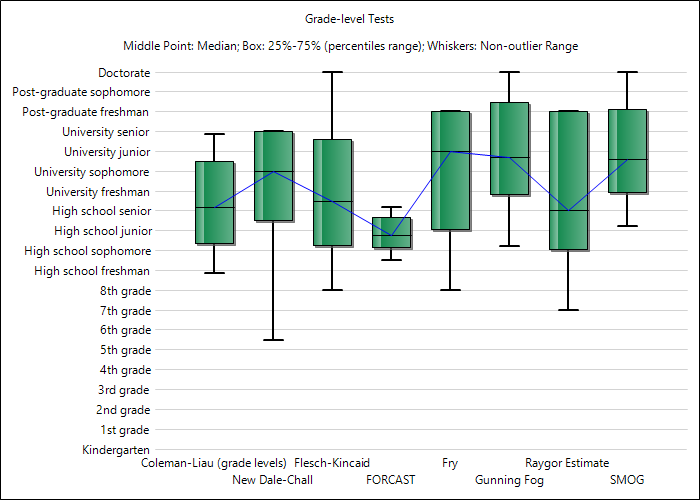


**Words Breakdown**

**Difficult Words**

| Website No. | Total Words | % of complex (3+ syllable) words | Complex (3+ syllable) words | % of long (6+ characters) words | Long (6+ characters) words | % of SMOG hard words | SMOG hard words | % of Fog hard words | Fog hard words | % of Dale-Chall unfamiliar words | Dale-Chall unfamiliar words |
| --- | --- | --- | --- | --- | --- | --- | --- | --- | --- | --- | --- |
| 1 | 329 | 20.1% | 66 | 36.2% | 119 | 20.4% | 67 | 19.1% | 63 | 31.5% | 108 |
| 2 | 1,190 | 15.9% | 189 | 29.6% | 352 | 16.2% | 193 | 15.2% | 181 | 22.4% | 267 |
| 3 | 3,609 | 27.5% | 991 | 42.5% | 1,535 | 28.7% | 1,037 | 26.3% | 950 | 40% | 1,442 |
| 4 | 80 | 25% | 20 | 45% | 36 | 25% | 20 | 22.5% | 18 | 40% | 32 |
| 5 | 3,904 | 17.5% | 684 | 34.9% | 1,361 | 17.7% | 692 | 16.1% | 629 | 27% | 1,070 |
| 6 | 511 | 15.1% | 77 | 29.2% | 149 | 15.7% | 80 | 14.7% | 75 | 22.1% | 113 |
| 7 | 3,637 | 26.4% | 959 | 41.4% | 1,505 | 29.3% | 1,066 | 23.4% | 850 | 39.2% | 1,425 |
| 8 | 3,906 | 26.4% | 1,031 | 42.8% | 1,670 | 27.7% | 1,082 | 24.4% | 954 | 39.5% | 1,543 |
| 9 | 2,965 | 15.7% | 465 | 31.6% | 938 | 16.1% | 476 | 14.8% | 440 | 20.7% | 615 |
| 10 | 2,425 | 25.9% | 627 | 40.9% | 993 | 26.8% | 651 | 23.3% | 564 | 37.6% | 911 |
| 11 | 1,091 | 20.5% | 224 | 37% | 404 | 21.1% | 230 | 18.9% | 206 | 28% | 306 |
| 12 | 571 | 22.8% | 130 | 38.5% | 220 | 24.7% | 141 | 21.7% | 124 | 34.8% | 201 |
| 13 | 2,180 | 13.5% | 295 | 30.9% | 674 | 14% | 306 | 11.3% | 247 | 22% | 483 |
| 14 | 1,222 | 13.6% | 166 | 30.4% | 372 | 13.7% | 168 | 12.9% | 158 | 18.7% | 229 |
| 15 | 233 | 26.6% | 62 | 41.2% | 96 | 27.5% | 64 | 25.8% | 60 | 36.5% | 85 |
| 16 | 821 | 18.9% | 155 | 32.9% | 270 | 18.9% | 155 | 17.7% | 145 | 28.1% | 231 |
| 17 | 6,799 | 28.1% | 1,908 | 44.2% | 3,006 | 29.5% | 2,006 | 25.9% | 1,760 | 39.6% | 2,695 |
| 18 | 391 | 18.7% | 73 | 33.2% | 130 | 18.7% | 73 | 18.2% | 71 | 30.4% | 119 |
| 19 | 4,050 | 24.1% | 976 | 39% | 1,578 | 25.1% | 1,017 | 21.8% | 881 | 35.2% | 1,426 |
| 20 | 1,069 | 24.1% | 258 | 47.4% | 507 | 25% | 267 | 19.6% | 210 | 37.2% | 398 |
| 21 | 1,379 | 28.4% | 392 | 40.6% | 560 | 28.7% | 396 | 26.6% | 367 | 36.6% | 505 |
| 22 | 958 | 21.1% | 202 | 36.1% | 346 | 21.4% | 205 | 20.6% | 197 | 32% | 307 |
| 23 | 909 | 28.8% | 262 | 40% | 364 | 31.1% | 283 | 26.5% | 241 | 39.2% | 356 |
| 24 | 3,691 | 27.8% | 1,026 | 42.5% | 1,570 | 30% | 1,109 | 25.7% | 947 | 39.6% | 1,463 |
| 25 | 297 | 18.9% | 56 | 33.3% | 99 | 18.9% | 56 | 17.5% | 52 | 27.6% | 82 |
| 26 | 1,034 | 25.1% | 260 | 42.5% | 439 | 25.2% | 261 | 22.8% | 236 | 36.6% | 378 |
| 27 | 535 | 22.2% | 119 | 41.9% | 224 | 22.2% | 119 | 20.6% | 110 | 34% | 182 |
| 28 | 838 | 17.1% | 143 | 32.8% | 275 | 18.1% | 152 | 15.5% | 130 | 27.6% | 231 |
| 29 | 427 | 13.8% | 59 | 25.1% | 107 | 13.8% | 59 | 13.1% | 56 | 21.1% | 90 |
| 30 | 697 | 18.7% | 130 | 34.4% | 240 | 18.7% | 130 | 18.2% | 127 | 30% | 209 |
| 31 | 2,569 | 22% | 565 | 40.8% | 1,048 | 22.9% | 589 | 20.3% | 521 | 34.4% | 884 |
| 32 | 2,069 | 20% | 414 | 32.2% | 667 | 25.3% | 524 | 18.2% | 376 | 27.3% | 565 |
| 33 | 1,007 | 17.4% | 175 | 29.9% | 301 | 18% | 181 | 16.7% | 168 | 27.4% | 276 |
| 34 | 2,631 | 26.9% | 709 | 43.1% | 1,135 | 29.6% | 778 | 24.4% | 642 | 40.3% | 1,059 |
| 35 | 3,213 | 19.7% | 632 | 33.9% | 1,089 | 22.9% | 735 | 18.5% | 596 | 30.4% | 976 |
| 36 | 737 | 18.3% | 135 | 35% | 258 | 18.5% | 136 | 17.4% | 128 | 29% | 214 |
| 37 | 456 | 14.7% | 67 | 31.1% | 142 | 14.7% | 67 | 14.3% | 65 | 21.3% | 97 |
| 38 | 405 | 13.6% | 55 | 27.2% | 110 | 13.8% | 56 | 13.3% | 54 | 17% | 69 |
| 39 | 15,704 | 13.9% | 2,179 | 26.8% | 4,208 | 14.3% | 2,238 | 12.8% | 2,016 | 20.9% | 3,279 |
| 40 | 1,735 | 15.2% | 263 | 30.8% | 534 | 15.3% | 265 | 14.5% | 252 | 25.1% | 435 |
| 41 | 660 | 16.4% | 108 | 30.8% | 203 | 16.8% | 111 | 15.5% | 102 | 23.9% | 158 |
| 42 | 3,686 | 24.7% | 909 | 40.7% | 1,500 | 25.3% | 934 | 23.2% | 854 | 38.2% | 1,407 |
| 43 | 1,342 | 13.5% | 181 | 27.6% | 370 | 14.2% | 190 | 12.8% | 172 | 15.6% | 210 |
| 44 | 744 | 13.7% | 102 | 27.6% | 205 | 14% | 104 | 13.4% | 100 | 18.3% | 137 |
| 45 | 2,800 | 24.3% | 680 | 41.2% | 1,154 | 26.1% | 732 | 21.9% | 612 | 38.9% | 1,089 |

**All Words**

| Word | Frequency | Website Count |
| --- | --- | --- |
| abandon | 1 | 1 |
| abandoned | 2 | 2 |
| Abduction | 1 | 1 |
| abduction/adduction | 2 | 1 |
| abductor | 1 | 1 |
| ability | 19 | 14 |
| Abkhazia | 1 | 1 |
| able | 52 | 18 |
| Abnormal | 8 | 5 |
| abnormality | 2 | 1 |
| abnormally | 5 | 3 |
| about | 81 | 24 |
| above | 29 | 16 |
| above-mentioned | 1 | 1 |
| abrade | 2 | 1 |
| abscess | 1 | 1 |
| absence | 11 | 6 |
| absent | 2 | 2 |
| absolute | 8 | 5 |
| absolutely | 3 | 2 |
| absorb | 1 | 1 |
| absorbable | 1 | 1 |
| absorbed | 1 | 1 |
| absorber | 1 | 1 |
| absorbers | 3 | 2 |
| absorbing | 1 | 1 |
| abundant | 1 | 1 |
| abuser | 2 | 1 |
| academic | 1 | 1 |
| accelerate | 2 | 2 |
| accelerated | 3 | 3 |
| acceptable | 2 | 2 |
| acceptance | 1 | 1 |
| accepted | 8 | 5 |
| access | 6 | 5 |
| accident | 1 | 1 |
| accidental | 1 | 1 |
| accompanied | 4 | 3 |
| accompanying | 1 | 1 |
| accomplished | 1 | 1 |
| accordance | 4 | 4 |
| according | 28 | 9 |
| accordingly | 7 | 5 |
| account | 3 | 3 |
| accumulation | 1 | 1 |
| accumulative | 1 | 1 |
| accuracy | 16 | 6 |
| accurate | 19 | 7 |
| accurately | 10 | 7 |
| Acelity | 1 | 1 |
| acetaminophen | 1 | 1 |
| achievable | 1 | 1 |
| achieve | 17 | 11 |
| achieved | 35 | 14 |
| achievement | 1 | 1 |
| achieves | 2 | 2 |
| achieving | 7 | 6 |
| Achilles | 1 | 1 |
| ACI | 13 | 2 |
| acid | 2 | 1 |
| ACL | 87 | 10 |
| ACL-deficiency | 1 | 1 |
| ACL-deficient | 3 | 1 |
| ACLs | 1 | 1 |
| acquired | 4 | 2 |
| across | 16 | 8 |
| act | 1 | 1 |
| acting | 4 | 2 |
| actions | 1 | 1 |
| activation | 1 | 1 |
| active | 69 | 29 |
| active/passive | 1 | 1 |
| actively | 2 | 2 |
| activities | 49 | 20 |
| activity | 36 | 16 |
| activity-related | 1 | 1 |
| activity/exercise | 1 | 1 |
| activity9 | 1 | 1 |
| Activmotion | 1 | 1 |
| acts | 3 | 2 |
| actual | 4 | 3 |
| actually | 25 | 2 |
| acute | 1 | 1 |
| adapt | 2 | 2 |
| adaptation | 1 | 1 |
| adapted | 7 | 5 |
| adaptive | 1 | 1 |
| add | 4 | 4 |
| added | 11 | 8 |
| addictive | 1 | 1 |
| adding | 6 | 5 |
| addition | 22 | 9 |
| additional | 19 | 11 |
| Additionally | 2 | 2 |
| address | 6 | 3 |
| addressed | 4 | 4 |
| addressing | 1 | 1 |
| adds | 4 | 2 |
| adduction | 10 | 4 |
| adductor | 1 | 1 |
| adequate | 9 | 9 |
| adequately | 1 | 1 |
| adhere | 1 | 1 |
| adhesion | 1 | 1 |
| adjacent | 3 | 3 |
| adjunct | 1 | 1 |
| adjunctive | 2 | 1 |
| adjust | 7 | 5 |
| adjusted | 5 | 5 |
| adjusting | 3 | 3 |
| adjustment | 4 | 4 |
| adjustments | 3 | 3 |
| ADLs | 1 | 1 |
| administered | 4 | 4 |
| administrative | 2 | 1 |
| admission | 5 | 3 |
| admissions | 1 | 1 |
| admitted | 3 | 3 |
| adopted | 2 | 2 |
| adult | 1 | 1 |
| adults | 1 | 1 |
| advance | 3 | 3 |
| advanced | 11 | 7 |
| advancement | 1 | 1 |
| advancements | 2 | 1 |
| advances | 9 | 4 |
| advantage | 9 | 8 |
| advantageous | 2 | 2 |
| advantages | 22 | 12 |
| advent | 2 | 1 |
| adverse | 2 | 1 |
| adversely | 1 | 1 |
| advertised | 1 | 1 |
| advice | 2 | 2 |
| Advil | 1 | 1 |
| advisable | 3 | 2 |
| advise | 2 | 2 |
| advised | 5 | 4 |
| advocated | 1 | 1 |
| aerobic | 1 | 1 |
| Aescula | 1 | 1 |
| Aesculap | 1 | 1 |
| aetiologically | 1 | 1 |
| affect | 17 | 12 |
| affected | 22 | 16 |
| affecting | 9 | 7 |
| affection | 3 | 1 |
| affects | 7 | 5 |
| afford | 1 | 1 |
| aforementioned | 1 | 1 |
| After | 381 | 41 |
| afternoon | 1 | 1 |
| afterwards | 6 | 3 |
| again | 20 | 8 |
| against | 13 | 8 |
| age | 73 | 18 |
| age-associated | 1 | 1 |
| aged | 7 | 5 |
| agent | 1 | 1 |
| ages | 2 | 2 |
| aggressive | 2 | 2 |
| Agneskirchner | 1 | 1 |
| ago | 9 | 3 |
| agree | 3 | 3 |
| ahead | 3 | 2 |
| Ahlback | 9 | 5 |
| Ahlberg | 1 | 1 |
| aid | 9 | 5 |
| aiding | 1 | 1 |
| aids | 3 | 3 |
| aim | 17 | 12 |
| aimed | 4 | 4 |
| aiming | 5 | 3 |
| aims | 7 | 4 |
| air | 1 | 1 |
| Airex | 1 | 1 |
| Akizuki | 2 | 2 |
| al | 23 | 4 |
| Al-though | 1 | 1 |
| al. | 107 | 13 |
| alarm | 1 | 1 |
| Albert | 1 | 1 |
| alcohol | 2 | 2 |
| alcoholic | 1 | 1 |
| alert | 1 | 1 |
| Aleve | 1 | 1 |
| Alex | 4 | 1 |
| algorithm | 4 | 3 |
| algorithms | 1 | 1 |
| align | 3 | 3 |
| aligned | 5 | 4 |
| alignment | 157 | 32 |
| alignment.1 | 1 | 1 |
| alignments | 1 | 1 |
| all | 99 | 26 |
| allergies | 1 | 1 |
| allergy | 1 | 1 |
| alleviate | 1 | 1 |
| alleviated | 1 | 1 |
| alleviates | 1 | 1 |
| alleviating | 1 | 1 |
| allo-graft | 1 | 1 |
| allobone | 1 | 1 |
| allocate | 1 | 1 |
| allogeneic | 2 | 1 |
| allograft | 17 | 8 |
| allografts | 2 | 2 |
| allow | 42 | 20 |
| allowed | 33 | 15 |
| allowing | 15 | 10 |
| allows | 43 | 16 |
| almost | 8 | 8 |
| alone | 17 | 7 |
| along | 18 | 16 |
| alongside | 1 | 1 |
| already | 12 | 8 |
| also | 150 | 34 |
| alta | 6 | 3 |
| alter | 8 | 7 |
| alteration | 2 | 2 |
| alterations | 3 | 2 |
| altered | 10 | 5 |
| Altering | 4 | 4 |
| alternate | 1 | 1 |
| alternation | 1 | 1 |
| alternative | 19 | 13 |
| Alternatively | 2 | 2 |
| alternatives | 1 | 1 |
| alters | 1 | 1 |
| although | 46 | 16 |
| altogether | 5 | 3 |
| always | 9 | 6 |
| am | 6 | 2 |
| amazingly | 1 | 1 |
| ambitions | 1 | 1 |
| amendments | 1 | 1 |
| America | 1 | 1 |
| AMIC | 2 | 1 |
| among | 9 | 6 |
| amongst | 2 | 2 |
| amount | 28 | 12 |
| amounts | 1 | 1 |
| amout | 1 | 1 |
| amoxicillin | 1 | 1 |
| amplitude | 1 | 1 |
| an | 467 | 44 |
| an-tero-medial | 1 | 1 |
| anaesthesia | 3 | 2 |
| anaesthetic | 20 | 6 |
| anaesthetics | 1 | 1 |
| anaesthetise | 1 | 1 |
| anaesthetist | 7 | 4 |
| anaesthetists | 2 | 1 |
| analgesia | 4 | 3 |
| analgesic | 1 | 1 |
| Analog | 6 | 4 |
| analogy | 1 | 1 |
| analysed | 3 | 1 |
| analyses | 7 | 4 |
| analysis | 23 | 7 |
| analyze | 2 | 2 |
| analyzed | 7 | 7 |
| anatomic | 5 | 4 |
| anatomical | 29 | 8 |
| anatomically | 3 | 2 |
| anatomy | 7 | 7 |
| anchors | 1 | 1 |
| ancillary | 1 | 1 |
| and | 2583 | 45 |
| and/or | 10 | 8 |
| Andriacchi | 1 | 1 |
| anesthesia | 32 | 14 |
| anesthesiologist | 3 | 2 |
| anesthesiologists | 1 | 1 |
| anesthetic | 3 | 3 |
| anesthetics | 1 | 1 |
| anesthetist | 2 | 2 |
| Angeles | 1 | 1 |
| angle | 94 | 23 |
| angle-stable | 3 | 1 |
| angles | 15 | 7 |
| angular | 8 | 5 |
| angulation | 7 | 3 |
| animation | 1 | 1 |
| ankle | 34 | 17 |
| ankles | 3 | 3 |
| ankylosis | 1 | 1 |
| annual | 1 | 1 |
| Another | 33 | 17 |
| anserine | 1 | 1 |
| anserinus | 12 | 3 |
| answer | 1 | 1 |
| answered | 1 | 1 |
| anterior | 53 | 13 |
| anterior-posterior | 2 | 1 |
| anteriorisation | 1 | 1 |
| Anteriorly | 12 | 4 |
| antero-posterior | 2 | 2 |
| anterolateral | 2 | 2 |
| anteromedial | 1 | 1 |
| anteroposterior | 14 | 6 |
| anti-clot | 1 | 1 |
| anti-inflammatorie | 1 | 1 |
| anti-inflammatories | 2 | 1 |
| anti-inflammatory | 9 | 7 |
| antibacterial | 1 | 1 |
| antibiotic | 4 | 3 |
| antibiotic-free | 1 | 1 |
| antibiotic-loaded | 1 | 1 |
| antibiotics | 31 | 11 |
| anticipated | 1 | 1 |
| anticoagulant | 1 | 1 |
| antimicrobial | 1 | 1 |
| antirheumatic | 2 | 1 |
| antiseptic | 1 | 1 |
| anxiety | 2 | 2 |
| any | 101 | 29 |
| Anybody | 1 | 1 |
| anyone | 1 | 1 |
| anything | 7 | 5 |
| AO | 1 | 1 |
| AP | 6 | 3 |
| ap-plied | 1 | 1 |
| apart | 4 | 3 |
| apex | 5 | 3 |
| apparatus | 1 | 1 |
| apparent | 1 | 1 |
| appealing | 1 | 1 |
| appear | 4 | 4 |
| appearance | 2 | 2 |
| appeared | 1 | 1 |
| appears | 4 | 4 |
| appetite | 1 | 1 |
| application | 5 | 4 |
| applications | 1 | 1 |
| applied | 19 | 9 |
| applies | 1 | 1 |
| apply | 9 | 4 |
| applying | 5 | 5 |
| appointment | 8 | 6 |
| appointments | 1 | 1 |
| apposition | 1 | 1 |
| appreciated | 1 | 1 |
| appreciation | 1 | 1 |
| approach | 11 | 8 |
| approached | 2 | 2 |
| approaches | 4 | 3 |
| approaching | 1 | 1 |
| appropriate | 33 | 19 |
| appropriately | 2 | 2 |
| approval | 3 | 3 |
| approved | 4 | 4 |
| approximately | 23 | 10 |
| approximation | 1 | 1 |
| April | 1 | 1 |
| aquacell | 4 | 1 |
| archiving | 1 | 1 |
| are | 600 | 45 |
| area | 40 | 21 |
| areas | 6 | 5 |
| argue | 1 | 1 |
| arise | 2 | 2 |
| arm | 1 | 1 |
| Armenia | 1 | 1 |
| armpit | 1 | 1 |
| around | 74 | 23 |
| Arrange | 4 | 2 |
| arranged | 1 | 1 |
| array | 2 | 2 |
| arrive | 1 | 1 |
| arriving | 1 | 1 |
| artery | 4 | 2 |
| arthoscopy | 1 | 1 |
| Arthrex | 5 | 3 |
| Arthrexmedical | 1 | 1 |
| arthritic | 43 | 14 |
| arthritis | 124 | 30 |
| arthritis-free | 1 | 1 |
| arthrop-lasty | 1 | 1 |
| arthropathies | 1 | 1 |
| arthroplasties | 7 | 4 |
| arthroplasty | 88 | 17 |
| arthroplsty | 1 | 1 |
| arthroscopic | 17 | 11 |
| arthroscopically | 4 | 4 |
| Arthroscopy | 26 | 11 |
| arthrosis | 8 | 4 |
| article | 5 | 4 |
| articles | 3 | 3 |
| articular | 58 | 19 |
| articulating | 1 | 1 |
| articulation | 1 | 1 |
| artificial | 10 | 4 |
| Artromot | 1 | 1 |
| as | 544 | 42 |
| Asada | 1 | 1 |
| ascending | 3 | 3 |
| ascent | 3 | 1 |
| aseptic | 4 | 2 |
| Asian | 1 | 1 |
| aside | 2 | 1 |
| Asik | 1 | 1 |
| ask | 6 | 5 |
| asked | 11 | 6 |
| asking | 1 | 1 |
| aspect | 25 | 10 |
| aspirate | 2 | 1 |
| Aspirin | 4 | 4 |
| Assess | 21 | 10 |
| assessed | 16 | 8 |
| assesses | 1 | 1 |
| assessment | 20 | 11 |
| assessments | 1 | 1 |
| assiduous | 2 | 1 |
| assigned | 1 | 1 |
| assist | 2 | 2 |
| assistance | 5 | 4 |
| assisted | 4 | 2 |
| assistive | 3 | 2 |
| associated | 59 | 16 |
| associates | 1 | 1 |
| association | 14 | 5 |
| assume | 1 | 1 |
| assumed | 5 | 4 |
| assuming | 1 | 1 |
| assures | 1 | 1 |
| ASTAOR | 1 | 1 |
| asymptomatic | 3 | 3 |
| at | 404 | 36 |
| athletes | 4 | 3 |
| athletic | 4 | 3 |
| athletics | 1 | 1 |
| atrophic | 1 | 1 |
| attach | 1 | 1 |
| Attached | 5 | 5 |
| attachment | 7 | 2 |
| attachments | 1 | 1 |
| attacks | 1 | 1 |
| attempt | 1 | 1 |
| attempted | 1 | 1 |
| attempts | 1 | 1 |
| attend | 1 | 1 |
| attention | 7 | 4 |
| attenuate | 1 | 1 |
| attenuation | 1 | 1 |
| attractive | 1 | 1 |
| attributable | 1 | 1 |
| augmentation | 1 | 1 |
| augments | 1 | 1 |
| August | 1 | 1 |
| Australia | 3 | 1 |
| Austria | 1 | 1 |
| author | 1 | 1 |
| author’s | 2 | 1 |
| authors | 28 | 10 |
| autoclave | 1 | 1 |
| Autogenous | 1 | 1 |
| autograft | 19 | 9 |
| autografts | 2 | 2 |
| autoimmune | 1 | 1 |
| autologous | 9 | 6 |
| automatic | 2 | 2 |
| automatically | 1 | 1 |
| AV | 3 | 1 |
| availability | 2 | 2 |
| available | 25 | 14 |
| avascular | 2 | 2 |
| average | 36 | 10 |
| Averaged | 1 | 1 |
| averages | 1 | 1 |
| Avoid | 54 | 19 |
| avoidance | 3 | 2 |
| avoided | 2 | 1 |
| avoiding | 4 | 4 |
| avoids | 2 | 2 |
| awake | 4 | 4 |
| aware | 5 | 4 |
| awareness | 1 | 1 |
| away | 21 | 12 |
| awesome | 1 | 1 |
| axes | 8 | 4 |
| axial | 19 | 7 |
| axially | 1 | 1 |
| axillary | 1 | 1 |
| axis | 131 | 20 |
| B | 2 | 1 |
| b-tricalcium | 1 | 1 |
| Babis | 1 | 1 |
| back | 59 | 14 |
| backward | 2 | 2 |
| backwards | 1 | 1 |
| bacteria | 3 | 1 |
| bacterial | 1 | 1 |
| bad | 8 | 4 |
| badge | 1 | 1 |
| Badhe | 1 | 1 |
| Bae | 1 | 1 |
| bag | 2 | 2 |
| bags | 1 | 1 |
| baja | 16 | 6 |
| balance | 5 | 3 |
| balanced | 3 | 1 |
| balancing | 3 | 2 |
| ball | 4 | 3 |
| ban-dage | 1 | 1 |
| band | 2 | 2 |
| bandage | 6 | 2 |
| bandaged | 1 | 1 |
| bandages | 6 | 5 |
| banks | 1 | 1 |
| bar | 1 | 1 |
| barrel-vault | 1 | 1 |
| barrier | 1 | 1 |
| barriers | 1 | 1 |
| bars | 1 | 1 |
| base | 3 | 3 |
| based | 39 | 17 |
| baseline | 5 | 2 |
| basic | 6 | 5 |
| basically | 6 | 4 |
| Basingstoke | 2 | 1 |
| basis | 4 | 4 |
| basketball | 1 | 1 |
| bath | 1 | 1 |
| bathe | 1 | 1 |
| bathing | 2 | 2 |
| Bauerfeind | 1 | 1 |
| be | 788 | 44 |
| be-tween | 1 | 1 |
| bear | 13 | 8 |
| bearing | 68 | 24 |
| bears | 2 | 1 |
| beautiful | 1 | 1 |
| became | 5 | 4 |
| because | 74 | 20 |
| become | 28 | 19 |
| becomes | 13 | 8 |
| becoming | 5 | 5 |
| bed | 18 | 9 |
| bed/plinth | 1 | 1 |
| been | 133 | 25 |
| before | 87 | 28 |
| began | 4 | 4 |
| begin | 16 | 11 |
| beginning | 6 | 6 |
| begins | 6 | 5 |
| begun | 2 | 2 |
| behavior | 1 | 1 |
| behind | 3 | 3 |
| being | 32 | 17 |
| Belarus | 1 | 1 |
| believe | 10 | 4 |
| below | 64 | 26 |
| belt | 1 | 1 |
| bend | 15 | 8 |
| bending | 6 | 5 |
| beneath | 5 | 4 |
| beneficial | 6 | 6 |
| benefit | 15 | 11 |
| benefits | 9 | 8 |
| Benjamin | 1 | 1 |
| bent | 6 | 5 |
| Besides | 4 | 3 |
| best | 24 | 15 |
| beta-tricalcium | 1 | 1 |
| betatricalcium | 1 | 1 |
| better | 39 | 16 |
| between | 116 | 29 |
| Beverly | 1 | 1 |
| beyond | 6 | 6 |
| bi-compartmental | 1 | 1 |
| bias | 2 | 2 |
| biceps | 1 | 1 |
| Bick | 1 | 1 |
| bicompartmental | 2 | 2 |
| BICS | 1 | 1 |
| bicycle | 1 | 1 |
| big | 14 | 3 |
| bigger | 2 | 2 |
| Bike | 2 | 2 |
| bilateral | 22 | 10 |
| bilaterally | 1 | 1 |
| bind | 3 | 3 |
| bioabsorbability | 2 | 2 |
| biocompatible | 1 | 1 |
| biofeedback | 1 | 1 |
| biologic | 2 | 2 |
| biological | 3 | 2 |
| biomechanical | 20 | 8 |
| biomechanically | 2 | 2 |
| biomechanics | 18 | 4 |
| biopsies | 1 | 1 |
| Bip-lanar | 1 | 1 |
| biphasic | 2 | 1 |
| biplanar | 12 | 4 |
| biplane | 7 | 3 |
| bipodal | 2 | 1 |
| birth | 3 | 2 |
| bit | 12 | 2 |
| Blackburne-Peel | 2 | 2 |
| bladder | 2 | 2 |
| blade | 6 | 3 |
| Bledsoe | 3 | 1 |
| bleed | 1 | 1 |
| bleeding | 8 | 5 |
| blind | 1 | 1 |
| blisters | 1 | 1 |
| block | 10 | 6 |
| blocking | 1 | 1 |
| blocks | 3 | 2 |
| blood | 50 | 16 |
| blood-derived | 2 | 1 |
| blood-thinning | 2 | 2 |
| bloody | 1 | 1 |
| Blount | 1 | 1 |
| blow | 1 | 1 |
| Blunt | 6 | 3 |
| BMI | 29 | 7 |
| board | 6 | 4 |
| boards | 1 | 1 |
| bodies | 1 | 1 |
| body | 38 | 25 |
| bodyweight | 1 | 1 |
| bolster | 1 | 1 |
| bolted | 1 | 1 |
| bolts | 3 | 1 |
| bone | 464 | 42 |
| bone-grafted | 1 | 1 |
| bone-implant | 1 | 1 |
| bone-on-bone | 1 | 1 |
| bone-related | 1 | 1 |
| bone/femur | 1 | 1 |
| bones | 72 | 19 |
| Bonin | 1 | 1 |
| Bonnin | 1 | 1 |
| bony | 14 | 8 |
| book | 1 | 1 |
| boot | 3 | 1 |
| boots | 3 | 1 |
| border | 10 | 7 |
| borders | 1 | 1 |
| born | 1 | 1 |
| borne | 1 | 1 |
| borrow | 1 | 1 |
| boss | 1 | 1 |
| BOSU | 1 | 1 |
| Both | 81 | 18 |
| bothers | 1 | 1 |
| bothersome | 1 | 1 |
| bottle | 1 | 1 |
| bottom | 4 | 2 |
| bought | 5 | 2 |
| boundaries | 1 | 1 |
| boundary | 3 | 2 |
| bow | 6 | 4 |
| bow-leg | 1 | 1 |
| bow-legged | 13 | 2 |
| bow-legs | 1 | 1 |
| bowed | 7 | 6 |
| Bowel | 4 | 2 |
| bowels | 1 | 1 |
| bowing | 3 | 3 |
| bowlegged | 8 | 5 |
| box | 3 | 2 |
| BPOWHTO | 2 | 1 |
| brace | 71 | 26 |
| braces | 3 | 2 |
| Bracing | 7 | 1 |
| bran | 1 | 1 |
| breach | 1 | 1 |
| break | 8 | 3 |
| break-away | 1 | 1 |
| breakage | 7 | 4 |
| breakdown | 1 | 1 |
| breaking | 2 | 2 |
| breakout | 1 | 1 |
| breaks | 1 | 1 |
| breakthrough | 1 | 1 |
| breathe | 1 | 1 |
| breathing | 3 | 3 |
| bridge | 4 | 2 |
| bridging | 1 | 1 |
| brief | 4 | 3 |
| Brigham | 1 | 1 |
| bright | 1 | 1 |
| brilliant | 1 | 1 |
| bring | 10 | 9 |
| bringing | 1 | 1 |
| brings | 4 | 4 |
| Brisbane | 4 | 1 |
| briskly | 1 | 1 |
| broaden | 1 | 1 |
| broke | 2 | 1 |
| broken | 7 | 4 |
| brought | 9 | 6 |
| Brouwer | 3 | 3 |
| bruising | 4 | 3 |
| buckling | 1 | 1 |
| build | 6 | 4 |
| building | 4 | 1 |
| builds | 1 | 1 |
| buildup | 1 | 1 |
| bulk | 1 | 1 |
| bump | 3 | 2 |
| bumps | 1 | 1 |
| bundle | 4 | 2 |
| bungee | 1 | 1 |
| bupivaine | 1 | 1 |
| burden | 1 | 1 |
| Burtsev | 1 | 1 |
| businessman | 1 | 1 |
| but | 164 | 33 |
| buttocks | 2 | 2 |
| buttress | 1 | 1 |
| buy | 6 | 4 |
| buying | 2 | 2 |
| BW | 4 | 1 |
| by | 368 | 44 |
| C | 2 | 1 |
| C-arm | 6 | 3 |
| C-arms | 1 | 1 |
| C-reactive | 1 | 1 |
| C-scans | 1 | 1 |
| cable | 3 | 2 |
| CAD | 1 | 1 |
| cadaver | 2 | 2 |
| cadaveric | 3 | 3 |
| cadavers | 1 | 1 |
| calcium | 3 | 1 |
| calcium-phosphate | 1 | 1 |
| calculate | 7 | 5 |
| calculated | 7 | 3 |
| calculates | 1 | 1 |
| calculation | 4 | 2 |
| calculations | 2 | 2 |
| calf | 5 | 3 |
| calibrated | 5 | 4 |
| call | 8 | 6 |
| called | 37 | 16 |
| Callus | 8 | 4 |
| calm | 1 | 1 |
| calming | 1 | 1 |
| calves | 5 | 2 |
| came | 3 | 1 |
| camera | 2 | 2 |
| can | 448 | 41 |
| can't | 6 | 2 |
| canal | 1 | 1 |
| cancelled | 1 | 1 |
| cancellous | 3 | 3 |
| cancer | 1 | 1 |
| candidate | 12 | 9 |
| candidates | 18 | 10 |
| cane | 3 | 3 |
| canes | 1 | 1 |
| canned | 1 | 1 |
| cannot | 6 | 6 |
| cap | 9 | 5 |
| capacity | 3 | 1 |
| capsule | 2 | 2 |
| capsuloligamentous | 2 | 1 |
| capture | 2 | 2 |
| captured | 1 | 1 |
| captures | 1 | 1 |
| car | 3 | 2 |
| carbon | 1 | 1 |
| Cardiovascular | 4 | 2 |
| care | 18 | 12 |
| career | 1 | 1 |
| careful | 5 | 3 |
| carefully | 8 | 6 |
| caregiver | 1 | 1 |
| carer | 1 | 1 |
| carried | 27 | 11 |
| carries | 3 | 2 |
| carry | 16 | 6 |
| carrying | 6 | 3 |
| Carticel | 1 | 1 |
| cartilage | 210 | 33 |
| cartilaginous | 8 | 3 |
| cas-es | 1 | 1 |
| case | 44 | 15 |
| cases | 114 | 22 |
| cast | 16 | 11 |
| casting | 3 | 2 |
| casts | 2 | 1 |
| catching | 4 | 3 |
| categorical | 1 | 1 |
| categories | 1 | 1 |
| category | 4 | 4 |
| catheter | 5 | 2 |
| Caton-Deschamps | 2 | 2 |
| caught | 1 | 1 |
| cause | 31 | 17 |
| caused | 17 | 11 |
| causes | 21 | 12 |
| causing | 10 | 7 |
| cautery | 5 | 2 |
| caution | 3 | 3 |
| cautioned | 1 | 1 |
| cautious | 1 | 1 |
| CE-marked | 1 | 1 |
| cease | 1 | 1 |
| Cedara | 1 | 1 |
| ceiling | 3 | 1 |
| Celebrex | 1 | 1 |
| cell | 7 | 2 |
| cells | 8 | 3 |
| cellulitis | 1 | 1 |
| cement | 9 | 4 |
| cemented | 1 | 1 |
| center | 39 | 12 |
| centered | 2 | 2 |
| centers | 1 | 1 |
| centimetre | 4 | 2 |
| centimetres | 1 | 1 |
| central | 1 | 1 |
| centrally | 2 | 1 |
| centre | 10 | 3 |
| centres | 1 | 1 |
| Centricity | 1 | 1 |
| century | 3 | 2 |
| ceramic | 3 | 2 |
| certain | 15 | 11 |
| certainly | 4 | 3 |
| certified | 1 | 1 |
| cessation | 1 | 1 |
| Chae | 1 | 1 |
| chain | 1 | 1 |
| chair | 2 | 2 |
| chal-lenge | 1 | 1 |
| challenge | 2 | 1 |
| challenges | 1 | 1 |
| challenging | 6 | 6 |
| Chalmers | 3 | 1 |
| Chambat | 1 | 1 |
| Chan | 2 | 1 |
| chance | 20 | 8 |
| chances | 5 | 3 |
| change | 36 | 13 |
| changed | 10 | 8 |
| changes | 51 | 12 |
| changing | 8 | 7 |
| chapter | 1 | 1 |
| character | 1 | 1 |
| characteristics | 1 | 1 |
| characterize | 1 | 1 |
| characterized | 3 | 2 |
| charge | 1 | 1 |
| chart | 3 | 3 |
| chatting | 2 | 2 |
| check | 14 | 11 |
| check-up | 1 | 1 |
| check-ups | 1 | 1 |
| checked | 8 | 6 |
| checking | 4 | 4 |
| Chen | 1 | 1 |
| chest | 2 | 2 |
| chevron | 3 | 1 |
| chi-square | 1 | 1 |
| Chicago | 1 | 1 |
| chiefly | 1 | 1 |
| child | 2 | 1 |
| children | 8 | 1 |
| chips | 2 | 2 |
| chisel | 6 | 2 |
| chisels | 11 | 4 |
| choice | 11 | 9 |
| choices | 4 | 4 |
| chondral | 23 | 10 |
| chondral/cartilage | 1 | 1 |
| chondrocyte | 5 | 3 |
| chondrocytes | 1 | 1 |
| chondrogenesis | 1 | 1 |
| chondropathy | 1 | 1 |
| chondroprotectors | 1 | 1 |
| choose | 2 | 2 |
| chop | 1 | 1 |
| chose | 1 | 1 |
| chosen | 4 | 2 |
| chronic | 20 | 9 |
| chronOS | 1 | 1 |
| CI | 7 | 1 |
| cigarette | 1 | 1 |
| Cincinnati | 2 | 2 |
| circles | 2 | 1 |
| circulating | 1 | 1 |
| circulation | 3 | 2 |
| circumference | 1 | 1 |
| circumstances | 1 | 1 |
| CIs | 1 | 1 |
| cited | 1 | 1 |
| clarify | 1 | 1 |
| classic | 1 | 1 |
| classification | 11 | 6 |
| classified | 1 | 1 |
| classifying | 1 | 1 |
| clean | 1 | 1 |
| Cleanse | 1 | 1 |
| cleanser | 1 | 1 |
| clear | 6 | 6 |
| clear-up | 1 | 1 |
| clearance | 2 | 2 |
| cleared | 1 | 1 |
| clearly | 3 | 2 |
| cleft | 1 | 1 |
| clever | 2 | 1 |
| clexane | 1 | 1 |
| clicking | 3 | 2 |
| clicks | 1 | 1 |
| climbing | 1 | 1 |
| clinic | 12 | 7 |
| Clinical | 77 | 16 |
| Clinically | 4 | 2 |
| clinicians | 1 | 1 |
| clinics | 3 | 1 |
| clips | 3 | 2 |
| clock | 1 | 1 |
| close | 13 | 8 |
| close-wedge | 3 | 1 |
| closed | 28 | 18 |
| closed-chain | 1 | 1 |
| closed-wedge | 11 | 5 |
| closedwedge | 1 | 1 |
| closely | 7 | 7 |
| closer | 5 | 5 |
| closes | 2 | 2 |
| closing | 90 | 22 |
| closing-wedge | 5 | 2 |
| closing/opening | 1 | 1 |
| closure | 6 | 4 |
| clot | 14 | 7 |
| cloth | 1 | 1 |
| clothing | 1 | 1 |
| clots | 13 | 9 |
| cm | 39 | 13 |
| cm-incision | 1 | 1 |
| cm-sized | 1 | 1 |
| cm. | 1 | 1 |
| cm2 | 4 | 1 |
| cms | 1 | 1 |
| co-amoxicillin | 4 | 1 |
| Co. | 1 | 1 |
| Cochrane | 2 | 2 |
| code | 6 | 1 |
| coded | 1 | 1 |
| codes | 1 | 1 |
| coefficient | 4 | 1 |
| cohort | 2 | 2 |
| cohorts | 1 | 1 |
| cold | 4 | 2 |
| collapse | 5 | 2 |
| collapses | 1 | 1 |
| collateral | 18 | 10 |
| collection | 2 | 1 |
| collide | 1 | 1 |
| color | 1 | 1 |
| colour | 2 | 1 |
| com-partment | 1 | 1 |
| com-plained | 1 | 1 |
| combination | 18 | 12 |
| combined | 29 | 14 |
| combining | 2 | 2 |
| come | 12 | 3 |
| comes | 2 | 1 |
| comfortable | 9 | 4 |
| comfortably | 5 | 3 |
| coming | 8 | 3 |
| commenced | 1 | 1 |
| commences | 1 | 1 |
| commercial | 1 | 1 |
| commercially | 1 | 1 |
| commit | 4 | 2 |
| commitment | 1 | 1 |
| Committee | 4 | 4 |
| committing | 1 | 1 |
| common | 57 | 25 |
| commonly | 44 | 20 |
| communicates | 1 | 1 |
| communication | 1 | 1 |
| Communications | 1 | 1 |
| community | 1 | 1 |
| comorbid | 1 | 1 |
| comorbidities | 1 | 1 |
| comorbidity | 2 | 2 |
| company | 4 | 4 |
| comparable | 9 | 6 |
| comparative | 4 | 3 |
| comparatively | 1 | 1 |
| compare | 8 | 3 |
| compared | 44 | 17 |
| comparing | 7 | 5 |
| comparison | 4 | 4 |
| compartment | 261 | 30 |
| compartmental | 2 | 2 |
| compartments | 19 | 11 |
| compensate | 2 | 2 |
| competition | 1 | 1 |
| competitive | 3 | 1 |
| complain | 2 | 2 |
| complained | 4 | 3 |
| complaining | 2 | 1 |
| complete | 31 | 17 |
| completed | 13 | 9 |
| completely | 22 | 9 |
| completeness | 1 | 1 |
| completing | 1 | 1 |
| complex | 22 | 5 |
| complexity | 1 | 1 |
| compliance | 4 | 4 |
| compliant | 2 | 2 |
| complicate | 2 | 2 |
| complicated | 5 | 3 |
| complicates | 1 | 1 |
| complication | 30 | 10 |
| complications | 85 | 26 |
| component | 4 | 3 |
| components | 2 | 1 |
| composed | 3 | 2 |
| composites | 1 | 1 |
| comprehensive | 3 | 2 |
| comprehensively | 2 | 1 |
| compress | 2 | 1 |
| compresses | 1 | 1 |
| compression | 35 | 13 |
| compressive | 8 | 5 |
| comprise | 1 | 1 |
| comprised | 4 | 3 |
| compromise | 2 | 2 |
| compromising | 1 | 1 |
| Computer | 15 | 6 |
| computer-assisted | 6 | 3 |
| concave | 2 | 1 |
| concentrate | 5 | 4 |
| concentration | 1 | 1 |
| concept | 2 | 2 |
| conceptual | 1 | 1 |
| concern | 2 | 2 |
| concerned | 3 | 2 |
| Concerning | 5 | 2 |
| concerns | 3 | 2 |
| concierge | 3 | 1 |
| conclude | 1 | 1 |
| concluded | 16 | 9 |
| conclusion | 2 | 2 |
| conclusions | 2 | 1 |
| conclusive | 1 | 1 |
| concomitant | 34 | 9 |
| concurrent | 5 | 2 |
| concurrently | 2 | 2 |
| condition | 17 | 9 |
| conditions | 17 | 13 |
| conducted | 1 | 1 |
| condylar | 1 | 1 |
| condyle | 9 | 6 |
| condyles | 5 | 2 |
| conference | 1 | 1 |
| confidence | 2 | 2 |
| confident | 3 | 3 |
| confidently | 1 | 1 |
| configuration | 1 | 1 |
| confining | 1 | 1 |
| confirm | 11 | 7 |
| confirmation | 1 | 1 |
| confirmed | 12 | 8 |
| confirming | 2 | 2 |
| conflicting | 1 | 1 |
| conflicts | 1 | 1 |
| Conforming | 1 | 1 |
| confounding | 1 | 1 |
| confusing | 1 | 1 |
| congenital | 6 | 1 |
| Congress | 2 | 1 |
| congruence | 1 | 1 |
| congruency | 1 | 1 |
| conjunction | 7 | 6 |
| connect | 1 | 1 |
| connected | 1 | 1 |
| connecting | 1 | 1 |
| connective | 1 | 1 |
| connects | 3 | 2 |
| consecutive | 2 | 2 |
| consensus | 5 | 4 |
| consent | 7 | 6 |
| consequently | 4 | 3 |
| conservative | 15 | 8 |
| conservatively | 3 | 3 |
| Conserving | 1 | 1 |
| consider | 12 | 10 |
| considerably | 3 | 2 |
| consideration | 7 | 6 |
| considerations | 1 | 1 |
| considered | 49 | 18 |
| considering | 6 | 5 |
| considers | 1 | 1 |
| consist | 1 | 1 |
| consistant | 1 | 1 |
| consisted | 1 | 1 |
| consistent | 1 | 1 |
| consistently | 1 | 1 |
| consisting | 1 | 1 |
| consists | 1 | 1 |
| consolidation | 8 | 7 |
| consolidations | 1 | 1 |
| constant | 1 | 1 |
| constipation | 2 | 1 |
| constitute | 1 | 1 |
| constituted | 1 | 1 |
| constitutes | 2 | 1 |
| constitutional | 2 | 2 |
| constricting | 2 | 2 |
| construct | 4 | 3 |
| construction | 2 | 2 |
| constructs | 3 | 1 |
| consultant | 6 | 4 |
| consultant’s | 1 | 1 |
| consultants | 1 | 1 |
| consultation | 1 | 1 |
| consultations | 2 | 1 |
| consulted | 1 | 1 |
| consumption | 1 | 1 |
| contact | 31 | 11 |
| containing | 2 | 2 |
| contains | 3 | 3 |
| contemplate | 1 | 1 |
| contemplating | 1 | 1 |
| context | 2 | 1 |
| continuance | 1 | 1 |
| continuation | 1 | 1 |
| continue | 18 | 11 |
| continued | 2 | 2 |
| continues | 5 | 5 |
| continuing | 2 | 2 |
| Continuous | 10 | 5 |
| continuously | 1 | 1 |
| contour | 6 | 3 |
| contoured | 2 | 2 |
| contraceptive | 3 | 2 |
| contract | 1 | 1 |
| contracting | 1 | 1 |
| contracture | 10 | 5 |
| contractures | 1 | 1 |
| contrain-dication | 1 | 1 |
| contraindicated | 4 | 3 |
| contraindication | 10 | 6 |
| Contraindications | 7 | 4 |
| contralateral | 3 | 2 |
| contrary | 3 | 3 |
| contrast | 5 | 5 |
| contribute | 6 | 6 |
| contributed | 3 | 3 |
| contributes | 1 | 1 |
| contributing | 2 | 2 |
| contribution | 1 | 1 |
| control | 32 | 15 |
| controlled | 14 | 10 |
| Controlling | 1 | 1 |
| controversial | 6 | 4 |
| controversies | 1 | 1 |
| controversy | 1 | 1 |
| convenient | 2 | 2 |
| conventional | 16 | 6 |
| Conventry | 3 | 2 |
| converging | 1 | 1 |
| converse | 2 | 2 |
| Conversely | 1 | 1 |
| conversion | 26 | 8 |
| conversions | 2 | 1 |
| converted | 8 | 3 |
| converting | 1 | 1 |
| cool | 1 | 1 |
| coolness | 1 | 1 |
| cools | 1 | 1 |
| coordinate | 1 | 1 |
| coordinator | 1 | 1 |
| COP | 1 | 1 |
| cope | 5 | 4 |
| cor-rection | 1 | 1 |
| cord | 4 | 3 |
| cords | 2 | 2 |
| corner | 6 | 4 |
| coronal | 33 | 10 |
| correct | 74 | 33 |
| correctable | 2 | 2 |
| corrected | 9 | 6 |
| Correcting | 11 | 8 |
| correction | 217 | 22 |
| corrections | 10 | 5 |
| corrective | 10 | 3 |
| correctly | 4 | 4 |
| corrects | 2 | 2 |
| correlated | 2 | 2 |
| correlation | 7 | 3 |
| correspond | 1 | 1 |
| corresponded | 1 | 1 |
| corresponding | 3 | 3 |
| corresponds | 1 | 1 |
| cortex | 57 | 12 |
| cortex-preserving | 1 | 1 |
| cortical | 12 | 7 |
| cortices | 1 | 1 |
| corticocancellous | 1 | 1 |
| cosmetic | 4 | 2 |
| cost | 3 | 3 |
| cotton | 1 | 1 |
| couch | 2 | 1 |
| cough | 2 | 2 |
| coughing | 1 | 1 |
| could | 43 | 14 |
| counter | 1 | 1 |
| CounterLock | 2 | 1 |
| countries | 5 | 4 |
| couple | 5 | 4 |
| coupled | 2 | 2 |
| course | 20 | 9 |
| Coventry | 13 | 9 |
| cover | 6 | 5 |
| coverage | 1 | 1 |
| covered | 4 | 4 |
| covering | 4 | 4 |
| covers | 1 | 1 |
| Cox | 2 | 1 |
| CPM | 7 | 3 |
| CPMM | 1 | 1 |
| crank | 1 | 1 |
| create | 5 | 4 |
| created | 8 | 7 |
| creates | 6 | 5 |
| creating | 6 | 5 |
| creation | 1 | 1 |
| creators | 1 | 1 |
| crescent | 2 | 1 |
| crest | 8 | 7 |
| criteria | 8 | 6 |
| criterion | 1 | 1 |
| critical | 5 | 3 |
| criticised | 1 | 1 |
| criticism | 1 | 1 |
| cross-country | 1 | 1 |
| Cross-training | 1 | 1 |
| CRP | 1 | 1 |
| CRPS | 1 | 1 |
| CRR | 5 | 1 |
| crucial | 5 | 3 |
| cruciate | 35 | 13 |
| crude | 2 | 1 |
| crudely | 1 | 1 |
| crutch | 3 | 2 |
| crutches | 60 | 24 |
| Cryo | 1 | 1 |
| Cryo-cuff | 2 | 2 |
| cryocuff | 9 | 4 |
| Cryotherapy | 2 | 2 |
| CT | 8 | 6 |
| cuff | 1 | 1 |
| culminate | 1 | 1 |
| Cumulative | 1 | 1 |
| cupboards | 1 | 1 |
| cups | 1 | 1 |
| cupula | 1 | 1 |
| curative | 1 | 1 |
| cure | 2 | 2 |
| cured | 1 | 1 |
| curettage | 1 | 1 |
| curette | 1 | 1 |
| curls | 1 | 1 |
| current | 12 | 8 |
| currently | 8 | 5 |
| curve | 2 | 1 |
| curved | 3 | 3 |
| curves | 1 | 1 |
| cushion | 1 | 1 |
| customized | 2 | 2 |
| cut | 93 | 26 |
| cuts | 8 | 7 |
| cutting | 15 | 12 |
| CW | 1 | 1 |
| CWHTO | 5 | 2 |
| CWO | 1 | 1 |
| cycle | 5 | 5 |
| cycles | 1 | 1 |
| cyclic | 1 | 1 |
| cyclical | 1 | 1 |
| cycling | 2 | 2 |
| cysts | 2 | 2 |
| daily | 9 | 8 |
| Dalteparin | 1 | 1 |
| damage | 56 | 26 |
| damaged | 52 | 21 |
| damages | 3 | 3 |
| damaging | 3 | 3 |
| damp | 1 | 1 |
| dampened | 3 | 2 |
| danger | 1 | 1 |
| dangerous | 1 | 1 |
| dangers | 1 | 1 |
| Danish | 1 | 1 |
| Darees | 1 | 1 |
| dark | 3 | 1 |
| DAS | 2 | 1 |
| Data | 26 | 10 |
| database | 4 | 3 |
| databases | 2 | 1 |
| date | 5 | 2 |
| Dawson | 1 | 1 |
| day | 54 | 17 |
| days | 46 | 22 |
| days/cm. | 1 | 1 |
| DBM | 1 | 1 |
| DBX | 1 | 1 |
| de-creasing | 1 | 1 |
| de-functioned | 1 | 1 |
| deadly | 2 | 2 |
| deal | 6 | 3 |
| dealing | 7 | 1 |
| dealt | 2 | 1 |
| death | 1 | 1 |
| debatable | 2 | 1 |
| debate | 2 | 2 |
| Debeyre | 1 | 1 |
| debilitating | 1 | 1 |
| debride | 1 | 1 |
| debridement | 8 | 2 |
| debridements | 2 | 2 |
| decade | 2 | 2 |
| decades | 2 | 2 |
| December | 1 | 1 |
| decide | 5 | 4 |
| decided | 5 | 3 |
| deciding | 2 | 2 |
| decision | 3 | 2 |
| Declaration | 2 | 2 |
| declare | 1 | 1 |
| decline | 1 | 1 |
| declined | 3 | 3 |
| declining | 1 | 1 |
| decompression | 3 | 3 |
| Decrease | 31 | 16 |
| decreased | 27 | 14 |
| decreases | 14 | 11 |
| decreasing | 11 | 7 |
| dedicated | 1 | 1 |
| deep | 30 | 19 |
| deeper | 1 | 1 |
| deeply | 1 | 1 |
| defect | 28 | 9 |
| defects | 20 | 8 |
| defend | 1 | 1 |
| deficiencies | 3 | 3 |
| deficiency | 33 | 7 |
| deficient | 15 | 3 |
| define | 2 | 2 |
| defined | 7 | 6 |
| definite | 1 | 1 |
| definitely | 1 | 1 |
| definition | 3 | 3 |
| definitive | 3 | 2 |
| deform | 1 | 1 |
| deformation | 11 | 6 |
| deformed | 1 | 1 |
| deformities | 32 | 6 |
| deformity | 110 | 27 |
| degenerate | 1 | 1 |
| degenerated | 2 | 2 |
| degeneration | 21 | 9 |
| degenerative | 42 | 12 |
| degradation | 1 | 1 |
| degree | 30 | 16 |
| degrees | 13 | 9 |
| Deie | 1 | 1 |
| Dejour | 1 | 1 |
| delay | 26 | 15 |
| delayed | 27 | 13 |
| delaying | 4 | 4 |
| delays | 7 | 6 |
| delighted | 1 | 1 |
| demand | 2 | 1 |
| demanding | 8 | 5 |
| demands | 2 | 2 |
| demineralized | 1 | 1 |
| demonstrate | 3 | 3 |
| demonstrated | 12 | 5 |
| demonstrates | 2 | 1 |
| density | 1 | 1 |
| department | 3 | 3 |
| depend | 8 | 7 |
| dependable | 1 | 1 |
| depended | 2 | 1 |
| dependency | 1 | 1 |
| dependent | 4 | 2 |
| depending | 31 | 22 |
| Depends | 8 | 6 |
| depressed | 1 | 1 |
| depth | 1 | 1 |
| DePuy | 2 | 1 |
| DePuySynthes | 7 | 1 |
| dermatomes | 1 | 1 |
| derotate | 3 | 1 |
| descending | 1 | 1 |
| describe | 2 | 2 |
| described | 24 | 11 |
| describes | 1 | 1 |
| description | 1 | 1 |
| design | 13 | 6 |
| designed | 15 | 8 |
| designs | 1 | 1 |
| desirable | 2 | 2 |
| desired | 15 | 12 |
| desk | 1 | 1 |
| desk-based | 2 | 2 |
| Despite | 11 | 9 |
| dessicans | 1 | 1 |
| destabilize | 1 | 1 |
| destroyed | 1 | 1 |
| destruction | 7 | 5 |
| detach | 1 | 1 |
| detached | 2 | 2 |
| detachment | 2 | 2 |
| detail | 3 | 3 |
| detailing | 1 | 1 |
| detect | 1 | 1 |
| detected | 3 | 1 |
| detecting | 1 | 1 |
| deteriorate | 1 | 1 |
| deteriorating | 1 | 1 |
| deterioration | 7 | 6 |
| determinants | 1 | 1 |
| determine | 18 | 13 |
| determined | 17 | 10 |
| determines | 4 | 3 |
| Determining | 3 | 3 |
| detrimental | 2 | 2 |
| Devas | 1 | 1 |
| develop | 17 | 15 |
| developed | 19 | 7 |
| developing | 9 | 7 |
| development | 10 | 7 |
| developmental | 2 | 2 |
| develops | 2 | 1 |
| deviates | 1 | 1 |
| deviation | 6 | 4 |
| device | 16 | 6 |
| devices | 16 | 10 |
| devised | 1 | 1 |
| DFO | 4 | 1 |
| diabetes | 5 | 5 |
| diabetic | 1 | 1 |
| diagnose | 1 | 1 |
| diagnosed | 6 | 4 |
| diagnosis | 10 | 4 |
| diagnostic | 10 | 6 |
| diagram | 1 | 1 |
| dial | 4 | 2 |
| diathermy | 1 | 1 |
| Diclofenac | 1 | 1 |
| did | 21 | 9 |
| didn't | 4 | 1 |
| diet | 4 | 3 |
| differ | 2 | 2 |
| differ-ence | 1 | 1 |
| difference | 24 | 14 |
| Differences | 14 | 6 |
| different | 47 | 15 |
| differentiate | 1 | 1 |
| differently | 2 | 2 |
| differs | 2 | 2 |
| difficult | 30 | 17 |
| difficulties | 6 | 5 |
| difficulty | 6 | 6 |
| Diffo | 1 | 1 |
| diffusion | 3 | 1 |
| Digital | 7 | 4 |
| dilaudid | 2 | 2 |
| dilute | 2 | 1 |
| dimension | 1 | 1 |
| dimensional | 1 | 1 |
| diminish | 1 | 1 |
| dinners | 1 | 1 |
| direct | 4 | 4 |
| directed | 9 | 6 |
| directing | 1 | 1 |
| direction | 7 | 6 |
| directly | 3 | 3 |
| dirty | 1 | 1 |
| disability | 5 | 5 |
| disabling | 1 | 1 |
| disadvantage | 3 | 2 |
| disadvantageous | 1 | 1 |
| disadvantages | 16 | 10 |
| disagreement | 1 | 1 |
| disaster | 1 | 1 |
| discharge | 9 | 7 |
| discharged | 8 | 7 |
| discharges | 1 | 1 |
| disclosure | 1 | 1 |
| discoid | 3 | 1 |
| discomfort | 15 | 11 |
| discourage | 1 | 1 |
| discovered | 3 | 2 |
| discrepancies | 3 | 2 |
| discrepancy | 7 | 4 |
| discretion | 3 | 2 |
| discuss | 14 | 10 |
| discussable | 1 | 1 |
| discussed | 10 | 7 |
| discusses | 1 | 1 |
| discussing | 1 | 1 |
| discussion | 3 | 2 |
| disease | 32 | 15 |
| disease-modifying | 1 | 1 |
| diseased | 3 | 3 |
| diseases | 6 | 4 |
| dislocations | 1 | 1 |
| disorder | 2 | 2 |
| disorders | 1 | 1 |
| displaced | 1 | 1 |
| disposal | 1 | 1 |
| dispute | 1 | 1 |
| disrupt | 2 | 2 |
| disrupted | 1 | 1 |
| disrupting | 1 | 1 |
| disruption | 2 | 2 |
| dissatisfaction | 2 | 2 |
| dissecans | 4 | 4 |
| dissection | 5 | 5 |
| dissections | 1 | 1 |
| distal | 47 | 15 |
| distally | 1 | 1 |
| distance | 9 | 9 |
| distances | 1 | 1 |
| distinct | 1 | 1 |
| distorted | 1 | 1 |
| distortion | 2 | 1 |
| distraction | 9 | 4 |
| Distractions | 1 | 1 |
| distributed | 3 | 3 |
| distributes | 1 | 1 |
| distribution | 10 | 3 |
| distributions | 2 | 1 |
| disturbance | 2 | 2 |
| diverging | 1 | 1 |
| diverse | 1 | 1 |
| divided | 6 | 3 |
| DMARDs | 4 | 1 |
| do | 104 | 15 |
| doctor | 31 | 12 |
| doctor’s | 1 | 1 |
| doctors | 4 | 1 |
| document | 1 | 1 |
| documentation | 4 | 3 |
| documented | 4 | 2 |
| does | 43 | 22 |
| doesn’t | 8 | 2 |
| doing | 26 | 6 |
| dome | 14 | 6 |
| dome-osteotomy | 4 | 1 |
| dome-shaped | 1 | 1 |
| domestic | 1 | 1 |
| don’t | 29 | 1 |
| done | 81 | 23 |
| donned | 3 | 1 |
| donor | 4 | 4 |
| door | 4 | 1 |
| dorsiflexion | 1 | 1 |
| dosages | 1 | 1 |
| dose | 1 | 1 |
| dose-dependent | 1 | 1 |
| dots | 1 | 1 |
| double | 9 | 4 |
| double-check | 1 | 1 |
| double-level | 1 | 1 |
| double-limb | 1 | 1 |
| doubled | 2 | 1 |
| doubles | 1 | 1 |
| down | 39 | 14 |
| downslope | 1 | 1 |
| Dr | 6 | 1 |
| Dr. | 1 | 1 |
| drain | 5 | 4 |
| drainage | 2 | 2 |
| drains | 1 | 1 |
| dramatic | 1 | 1 |
| dramatically | 1 | 1 |
| draped | 1 | 1 |
| draping | 2 | 1 |
| drastic | 1 | 1 |
| draw | 4 | 1 |
| drawbacks | 2 | 2 |
| drawing | 2 | 2 |
| drawn | 11 | 6 |
| dressing | 15 | 7 |
| dressings | 10 | 4 |
| drew | 1 | 1 |
| drift | 2 | 2 |
| drill | 9 | 3 |
| drilled | 3 | 3 |
| drilling | 4 | 2 |
| drink | 6 | 5 |
| drinking | 2 | 1 |
| drinks | 1 | 1 |
| drip | 1 | 1 |
| drive | 9 | 4 |
| Driving | 3 | 2 |
| drops | 1 | 1 |
| drug | 1 | 1 |
| drugs | 5 | 3 |
| drugs-such | 1 | 1 |
| dry | 4 | 3 |
| Dual | 5 | 4 |
| Due | 53 | 17 |
| Dugdale | 8 | 6 |
| Duivenvoorden | 1 | 1 |
| dull | 1 | 1 |
| durable | 1 | 1 |
| duration | 2 | 2 |
| during | 134 | 36 |
| Dutch | 1 | 1 |
| duties | 2 | 2 |
| DVT | 3 | 2 |
| DVTs | 1 | 1 |
| Dynadisc | 1 | 1 |
| dynamic | 7 | 4 |
| dynamics | 8 | 2 |
| dysgalactiae | 1 | 1 |
| dysplasia | 1 | 1 |
| dystrophy | 2 | 2 |
| e.g. | 8 | 4 |
| each | 37 | 19 |
| earlier | 4 | 4 |
| earliest | 3 | 3 |
| early | 61 | 17 |
| early-stage | 4 | 4 |
| ease | 2 | 2 |
| eases | 1 | 1 |
| easier | 17 | 11 |
| easily | 8 | 6 |
| easing | 2 | 2 |
| Eastern | 1 | 1 |
| easy | 7 | 5 |
| easy-to-treat | 1 | 1 |
| eat | 6 | 6 |
| Eating | 3 | 1 |
| Eccentric | 3 | 2 |
| eccentrically | 1 | 1 |
| economic | 1 | 1 |
| edema | 2 | 2 |
| edge | 10 | 7 |
| edges | 3 | 3 |
| education | 4 | 3 |
| effect | 21 | 9 |
| effective | 36 | 14 |
| effectively | 3 | 3 |
| effectiveness | 8 | 5 |
| effects | 12 | 7 |
| efficacious | 1 | 1 |
| efficacy | 2 | 1 |
| efficiency | 1 | 1 |
| efficient | 3 | 2 |
| effort | 3 | 3 |
| efforts | 1 | 1 |
| effusion | 3 | 2 |
| effusions | 1 | 1 |
| eight | 13 | 8 |
| eighty-seven | 1 | 1 |
| Eighty-six | 2 | 2 |
| either | 57 | 25 |
| Ekhtiari | 1 | 1 |
| El-Galaly | 2 | 1 |
| elastic | 6 | 1 |
| elastically | 1 | 1 |
| elbow | 2 | 1 |
| elderly | 2 | 2 |
| elect | 1 | 1 |
| electing | 1 | 1 |
| elective | 1 | 1 |
| electric | 3 | 2 |
| electrical | 1 | 1 |
| electrocautery | 1 | 1 |
| element | 4 | 3 |
| elements | 1 | 1 |
| elevate | 1 | 1 |
| elevated | 17 | 12 |
| elevates | 1 | 1 |
| elevating | 3 | 2 |
| elevation | 4 | 4 |
| elevator | 1 | 1 |
| elicit | 2 | 1 |
| eliminate | 5 | 2 |
| eliminated | 1 | 1 |
| eliminating | 1 | 1 |
| elliptical | 1 | 1 |
| Elmslie-Trillat | 1 | 1 |
| else | 2 | 1 |
| else’s | 1 | 1 |
| EM | 2 | 1 |
| embolism | 4 | 3 |
| emergency | 3 | 2 |
| emerging | 3 | 1 |
| eminence | 1 | 1 |
| Emphasis | 1 | 1 |
| empiric | 1 | 1 |
| employ | 1 | 1 |
| employed | 1 | 1 |
| employment | 1 | 1 |
| employs | 1 | 1 |
| empty | 3 | 2 |
| enable | 3 | 3 |
| enabled | 1 | 1 |
| enables | 2 | 2 |
| enabling | 1 | 1 |
| encountered | 1 | 1 |
| encourage | 3 | 2 |
| encouraged | 2 | 2 |
| encourages | 1 | 1 |
| encouraging | 2 | 2 |
| end | 33 | 15 |
| end-stage | 2 | 2 |
| endeavors | 1 | 1 |
| ended | 2 | 2 |
| endoprosthetic | 1 | 1 |
| endpoints | 1 | 1 |
| ends | 11 | 8 |
| Endurance | 4 | 3 |
| engagement | 1 | 1 |
| engineered | 1 | 1 |
| English-language | 1 | 1 |
| enhance | 3 | 3 |
| enhanced | 3 | 3 |
| enhancement | 1 | 1 |
| enhances | 1 | 1 |
| enhancing | 2 | 2 |
| enjoy | 2 | 2 |
| enough | 15 | 8 |
| enriched | 1 | 1 |
| ensure | 18 | 11 |
| ensured | 2 | 2 |
| ensures | 1 | 1 |
| entails | 1 | 1 |
| entered | 2 | 1 |
| enthusiasm | 1 | 1 |
| entire | 3 | 3 |
| entirely | 1 | 1 |
| environment | 14 | 10 |
| epidural | 5 | 2 |
| epidurals | 2 | 1 |
| epiphisis | 1 | 1 |
| epiphyseal | 1 | 1 |
| epiphysis | 3 | 3 |
| episodes | 1 | 1 |
| eponymous | 1 | 1 |
| equal | 9 | 7 |
| equally | 2 | 2 |
| equation | 1 | 1 |
| equines | 1 | 1 |
| equipment | 1 | 1 |
| equivalent | 2 | 2 |
| eradicate | 4 | 1 |
| eradicating | 1 | 1 |
| eradication | 1 | 1 |
| erosion | 1 | 1 |
| errands | 1 | 1 |
| error | 1 | 1 |
| errors | 2 | 2 |
| especially | 17 | 9 |
| essence | 2 | 2 |
| essential | 9 | 7 |
| established | 3 | 3 |
| estimate | 2 | 2 |
| estimated | 5 | 5 |
| estimates | 2 | 1 |
| estimating | 1 | 1 |
| et | 130 | 14 |
| etc. | 2 | 2 |
| ethical | 1 | 1 |
| Ethics | 2 | 2 |
| etiologies | 1 | 1 |
| Europe | 4 | 4 |
| European | 3 | 2 |
| evaluate | 8 | 6 |
| evaluated | 15 | 5 |
| evaluating | 4 | 3 |
| evaluation | 15 | 6 |
| evaluations | 1 | 1 |
| even | 56 | 19 |
| evening | 2 | 2 |
| evenly | 6 | 6 |
| event | 1 | 1 |
| events | 1 | 1 |
| eventually | 5 | 4 |
| ever | 2 | 1 |
| Every | 21 | 10 |
| everyday | 1 | 1 |
| everyone | 2 | 2 |
| everything | 2 | 1 |
| evidence | 14 | 8 |
| evidenced | 3 | 2 |
| evidences | 5 | 3 |
| evident | 3 | 3 |
| evolution | 4 | 1 |
| evolved | 1 | 1 |
| exacerbated | 1 | 1 |
| exact | 9 | 5 |
| Exactly | 5 | 3 |
| exam | 5 | 3 |
| examination | 10 | 8 |
| examine | 3 | 3 |
| examined | 1 | 1 |
| example | 18 | 12 |
| examples | 1 | 1 |
| exceed | 2 | 1 |
| exceeded | 1 | 1 |
| exceeding | 4 | 2 |
| Excel | 1 | 1 |
| excellent | 20 | 12 |
| except | 4 | 3 |
| excess | 1 | 1 |
| excessive | 17 | 11 |
| exchange | 1 | 1 |
| exchanged | 1 | 1 |
| exchanging | 1 | 1 |
| excision | 1 | 1 |
| exclude | 6 | 4 |
| excluded | 4 | 3 |
| exclusion | 2 | 2 |
| exclusively | 1 | 1 |
| exercise | 27 | 9 |
| exercises | 62 | 26 |
| exerted | 3 | 3 |
| exhausted | 1 | 1 |
| exist | 5 | 3 |
| existing | 2 | 2 |
| exists | 7 | 6 |
| exit | 1 | 1 |
| exited | 1 | 1 |
| expand | 1 | 1 |
| expanded | 1 | 1 |
| expanding | 1 | 1 |
| expect | 15 | 7 |
| expectation | 4 | 3 |
| expectations | 2 | 2 |
| expected | 6 | 6 |
| experience | 20 | 12 |
| experienced | 6 | 5 |
| experiences | 1 | 1 |
| experiment | 1 | 1 |
| experimental | 3 | 2 |
| explain | 6 | 5 |
| explained | 1 | 1 |
| explaining | 2 | 2 |
| explains | 1 | 1 |
| explanation | 1 | 1 |
| exploring | 1 | 1 |
| expose | 6 | 4 |
| exposed | 5 | 5 |
| exposure | 6 | 5 |
| expressed | 2 | 2 |
| extend | 2 | 2 |
| extended | 6 | 6 |
| extending | 3 | 3 |
| extends | 1 | 1 |
| extension | 38 | 17 |
| Extensive | 1 | 1 |
| extensor | 3 | 3 |
| extent | 8 | 7 |
| external | 30 | 11 |
| extra | 10 | 6 |
| extra-articular | 2 | 1 |
| extraarticular | 1 | 1 |
| extreme | 3 | 2 |
| extremely | 5 | 2 |
| extremes | 1 | 1 |
| extremities | 8 | 4 |
| extremity | 20 | 8 |
| eye-balling | 1 | 1 |
| face | 2 | 2 |
| facilitate | 1 | 1 |
| facilitates | 2 | 2 |
| facility | 1 | 1 |
| facing | 2 | 1 |
| fact | 11 | 5 |
| factor | 18 | 9 |
| factors | 38 | 17 |
| Faculty | 1 | 1 |
| fade | 3 | 3 |
| fades | 1 | 1 |
| fail | 4 | 3 |
| failed | 12 | 7 |
| failing | 1 | 1 |
| fails | 2 | 2 |
| failure | 37 | 15 |
| failures | 1 | 1 |
| fair | 1 | 1 |
| fairly | 2 | 2 |
| fall | 4 | 4 |
| falling | 1 | 1 |
| falls | 1 | 1 |
| familiar | 2 | 2 |
| familiarity | 1 | 1 |
| family | 4 | 4 |
| fantastic | 1 | 1 |
| far | 7 | 4 |
| farm | 1 | 1 |
| farmer | 2 | 1 |
| fascia | 6 | 4 |
| Fascial | 1 | 1 |
| fashion | 6 | 5 |
| fashioned | 2 | 1 |
| fastened | 3 | 2 |
| faster | 4 | 3 |
| fatigue | 7 | 1 |
| fatter | 1 | 1 |
| favorable | 4 | 4 |
| favorably | 1 | 1 |
| favorite | 2 | 2 |
| favour | 3 | 2 |
| favourable | 5 | 2 |
| favouring | 1 | 1 |
| favours | 1 | 1 |
| FE | 2 | 1 |
| FEA | 8 | 1 |
| fear | 1 | 1 |
| feasible | 1 | 1 |
| feather | 1 | 1 |
| feel | 20 | 11 |
| feeling | 1 | 1 |
| feels | 1 | 1 |
| feet | 2 | 2 |
| fell | 1 | 1 |
| fellowship | 2 | 1 |
| felt | 2 | 2 |
| female | 4 | 4 |
| females | 1 | 1 |
| femoral | 52 | 17 |
| femoris | 3 | 1 |
| femoropatellar | 1 | 1 |
| femorotibial | 8 | 4 |
| femur | 77 | 22 |
| femur’s | 2 | 1 |
| fever | 6 | 6 |
| few | 31 | 13 |
| fewer | 6 | 6 |
| fibre | 3 | 3 |
| fibrin-ACI | 2 | 1 |
| fibrocartilage | 1 | 1 |
| Fibrosis | 1 | 1 |
| fibrous | 1 | 1 |
| fibula | 13 | 4 |
| fibular | 28 | 9 |
| field | 5 | 4 |
| fifteen | 4 | 1 |
| fifth | 1 | 1 |
| fifty-eight | 1 | 1 |
| fighting | 1 | 1 |
| figure | 9 | 5 |
| files | 1 | 1 |
| fill | 22 | 14 |
| filled | 7 | 6 |
| filler | 1 | 1 |
| filling | 5 | 4 |
| fills | 5 | 5 |
| film | 2 | 1 |
| films | 5 | 3 |
| final | 18 | 10 |
| Finally | 11 | 9 |
| find | 15 | 11 |
| finding | 4 | 4 |
| findings | 11 | 5 |
| fine | 3 | 2 |
| fine-tuned | 1 | 1 |
| finely | 1 | 1 |
| finish | 1 | 1 |
| finished | 2 | 2 |
| finite | 2 | 1 |
| fire | 1 | 1 |
| firm | 1 | 1 |
| firmly | 2 | 1 |
| first | 98 | 26 |
| Firstly | 2 | 2 |
| fit | 5 | 3 |
| Fitness | 4 | 2 |
| fitted | 1 | 1 |
| fitting | 3 | 3 |
| five | 13 | 9 |
| five-inch | 1 | 1 |
| fix | 5 | 2 |
| fixation | 114 | 17 |
| fixator | 22 | 8 |
| fixators | 2 | 1 |
| fixed | 23 | 13 |
| fixed-bearing | 1 | 1 |
| fixes | 1 | 1 |
| fixing | 4 | 2 |
| fixture | 1 | 1 |
| flap | 5 | 3 |
| flaps | 1 | 1 |
| flare | 4 | 2 |
| flat | 8 | 5 |
| flatten | 3 | 1 |
| flattened | 1 | 1 |
| flattening | 3 | 1 |
| flattens | 1 | 1 |
| flatter | 1 | 1 |
| flex | 3 | 1 |
| flexed | 1 | 1 |
| flexibility | 4 | 2 |
| flexible | 3 | 2 |
| flexing | 3 | 2 |
| flexion | 42 | 14 |
| flexion/extension | 2 | 1 |
| flight | 2 | 1 |
| Floerkemeier | 2 | 2 |
| floor | 4 | 3 |
| flow | 3 | 3 |
| flu | 1 | 1 |
| fluid | 2 | 2 |
| fluids | 3 | 1 |
| fluoroscope | 1 | 1 |
| fluoroscopes | 1 | 1 |
| fluoroscopic | 4 | 3 |
| fluoroscopy | 11 | 6 |
| flush | 1 | 1 |
| fly | 1 | 1 |
| focal | 4 | 2 |
| focus | 8 | 6 |
| focused | 3 | 2 |
| focuses | 2 | 2 |
| fold | 1 | 1 |
| folic | 1 | 1 |
| follow | 16 | 10 |
| follow-ing | 1 | 1 |
| follow-up | 58 | 20 |
| followed | 17 | 12 |
| followed-up | 1 | 1 |
| following | 73 | 26 |
| followings | 1 | 1 |
| follows | 2 | 2 |
| food | 3 | 3 |
| foods | 1 | 1 |
| foot | 16 | 8 |
| football | 1 | 1 |
| footdrop | 1 | 1 |
| footwear | 1 | 1 |
| for | 851 | 45 |
| force | 20 | 7 |
| forced | 1 | 1 |
| forces | 27 | 12 |
| forcing | 1 | 1 |
| forearm | 2 | 2 |
| foreign | 2 | 1 |
| forever | 1 | 1 |
| form | 18 | 11 |
| formation | 7 | 7 |
| formed | 3 | 3 |
| former | 3 | 3 |
| Formerly | 1 | 1 |
| forming | 2 | 2 |
| forms | 3 | 3 |
| forties | 1 | 1 |
| Fortunately | 1 | 1 |
| Forty | 1 | 1 |
| forward | 6 | 2 |
| forward/backward | 2 | 1 |
| forwards | 1 | 1 |
| fossa | 1 | 1 |
| found | 59 | 13 |
| Foundation | 2 | 2 |
| four | 13 | 9 |
| four-to | 1 | 1 |
| fourteen | 1 | 1 |
| fourth | 3 | 3 |
| fourth-generation | 1 | 1 |
| Fowler | 1 | 1 |
| fracture | 46 | 13 |
| fracture-protecting | 1 | 1 |
| fractured | 1 | 1 |
| fractures | 19 | 9 |
| fragility | 1 | 1 |
| fragment | 6 | 4 |
| fragments | 3 | 2 |
| frame | 14 | 6 |
| frames | 3 | 3 |
| France | 1 | 1 |
| free | 11 | 6 |
| freedom | 1 | 1 |
| freely | 1 | 1 |
| frees | 1 | 1 |
| freeze | 1 | 1 |
| freezer | 1 | 1 |
| freezing | 1 | 1 |
| frequency | 4 | 3 |
| frequent | 5 | 2 |
| frequently | 11 | 8 |
| fresh | 1 | 1 |
| friction | 2 | 1 |
| friend | 1 | 1 |
| friends | 1 | 1 |
| Frolov | 1 | 1 |
| from | 314 | 45 |
| front | 30 | 13 |
| frontal | 9 | 3 |
| frontward | 1 | 1 |
| frozen | 6 | 4 |
| fruits | 1 | 1 |
| Fujisawa | 9 | 6 |
| Fujisawa-Region | 1 | 1 |
| fulfil | 1 | 1 |
| fulfills | 1 | 1 |
| Fulkerson | 1 | 1 |
| full | 62 | 31 |
| full-leg | 2 | 1 |
| full-length | 1 | 1 |
| full-time | 1 | 1 |
| full-weight | 1 | 1 |
| fully | 19 | 11 |
| function | 34 | 18 |
| functional | 42 | 14 |
| functioning | 5 | 2 |
| fundamental | 1 | 1 |
| funded | 1 | 1 |
| furniture | 1 | 1 |
| further | 44 | 21 |
| Furthermore | 18 | 8 |
| furthest | 1 | 1 |
| fusion | 3 | 2 |
| future | 14 | 11 |
| G | 1 | 1 |
| GA | 1 | 1 |
| Gaasbeek | 1 | 1 |
| gabapentin | 1 | 1 |
| gage | 1 | 1 |
| gain | 1 | 1 |
| gained | 5 | 5 |
| gait | 46 | 9 |
| gaits | 1 | 1 |
| Game | 1 | 1 |
| gamma-ray | 1 | 1 |
| Gandhi | 1 | 1 |
| gap | 65 | 15 |
| gaps | 2 | 2 |
| gastroc/soleus | 1 | 1 |
| gastrocnemius | 3 | 2 |
| gathered | 2 | 2 |
| gauge | 1 | 1 |
| gauze | 1 | 1 |
| gender | 2 | 2 |
| general | 38 | 23 |
| generally | 29 | 19 |
| generate | 2 | 2 |
| generating | 2 | 1 |
| generation | 1 | 1 |
| genesis | 1 | 1 |
| Genetic | 2 | 2 |
| genetically | 2 | 2 |
| genovarum | 1 | 1 |
| gentamicin | 1 | 1 |
| gentamicin-impregnated | 1 | 1 |
| gentamicin/vancomycin-impregnated | 1 | 1 |
| gentle | 3 | 3 |
| gentleman | 3 | 1 |
| Gently | 8 | 7 |
| genu | 4 | 2 |
| GenuTrain | 1 | 1 |
| geometry | 4 | 2 |
| germ | 3 | 2 |
| German | 1 | 1 |
| Germans | 1 | 1 |
| Germany | 1 | 1 |
| get | 48 | 11 |
| gets | 6 | 3 |
| getting | 14 | 5 |
| Giancarlo | 1 | 1 |
| GIBP | 3 | 1 |
| Giuseffi | 1 | 1 |
| give | 21 | 11 |
| given | 21 | 12 |
| gives | 8 | 5 |
| giving | 5 | 4 |
| glad | 3 | 1 |
| Glashow | 1 | 1 |
| glide | 4 | 3 |
| glides | 2 | 1 |
| gliding | 5 | 4 |
| glucosamine | 1 | 1 |
| Gluteal | 1 | 1 |
| gluteus | 1 | 1 |
| go | 50 | 13 |
| go-narthrosis | 1 | 1 |
| goal | 15 | 13 |
| goals | 7 | 7 |
| goes | 6 | 2 |
| going | 32 | 4 |
| gold | 3 | 2 |
| golden | 4 | 3 |
| golf | 1 | 1 |
| gonarthritis | 6 | 1 |
| gonarthrosis | 9 | 5 |
| gone | 6 | 3 |
| Good | 97 | 19 |
| got | 18 | 1 |
| gown | 1 | 1 |
| Goyang | 1 | 1 |
| GP | 3 | 3 |
| grab | 1 | 1 |
| gracilis | 1 | 1 |
| grade | 23 | 12 |
| grades | 1 | 1 |
| gradual | 6 | 4 |
| gradual-ly | 1 | 1 |
| gradually | 18 | 11 |
| graduate | 1 | 1 |
| graft | 55 | 25 |
| grafted | 2 | 2 |
| grafting | 9 | 6 |
| grafts | 6 | 4 |
| grains | 1 | 1 |
| gravity | 1 | 1 |
| great | 13 | 7 |
| greater | 21 | 10 |
| greatest | 1 | 1 |
| greatly | 3 | 3 |
| Greenwood | 1 | 1 |
| greyhounds | 1 | 1 |
| grinding | 2 | 2 |
| groove | 4 | 2 |
| grooves | 3 | 1 |
| gross | 4 | 1 |
| ground | 3 | 3 |
| group | 59 | 11 |
| groups | 20 | 8 |
| grow | 4 | 4 |
| growing | 6 | 4 |
| growth | 9 | 4 |
| Gstöttner | 1 | 1 |
| guarantee | 3 | 3 |
| guidance | 4 | 3 |
| guide | 34 | 13 |
| guide-pin | 1 | 1 |
| guided | 6 | 3 |
| guideline | 5 | 4 |
| guidelines | 11 | 5 |
| guider | 1 | 1 |
| guiders | 1 | 1 |
| guides | 1 | 1 |
| Guy’s | 1 | 1 |
| Gwinner | 1 | 1 |
| gym | 1 | 1 |
| H.T.O. | 2 | 1 |
| had | 130 | 19 |
| haematoma | 2 | 2 |
| half | 7 | 7 |
| half-day | 1 | 1 |
| half-millimetre | 1 | 1 |
| half-moon | 1 | 1 |
| hallmarks | 1 | 1 |
| halt | 2 | 2 |
| halting | 1 | 1 |
| hamstring | 2 | 1 |
| hamstring/calf | 1 | 1 |
| hamstrings | 6 | 3 |
| Hamstrings/Gluteals | 2 | 1 |
| Han | 1 | 1 |
| Hand | 9 | 7 |
| hand-in-hand | 1 | 1 |
| handful | 1 | 1 |
| handled | 1 | 1 |
| hands | 4 | 4 |
| hangs | 1 | 1 |
| HAp | 2 | 2 |
| haphazard | 1 | 1 |
| happen | 4 | 4 |
| happens | 4 | 2 |
| happy | 6 | 2 |
| hard | 2 | 2 |
| hardware | 18 | 9 |
| harvest | 2 | 2 |
| harvested | 1 | 1 |
| has | 253 | 38 |
| hasn’t | 3 | 1 |
| hassle | 1 | 1 |
| have | 352 | 40 |
| haven’t | 5 | 2 |
| Having | 27 | 12 |
| hazardous | 1 | 1 |
| He | 43 | 11 |
| head | 29 | 10 |
| heads | 1 | 1 |
| heal | 32 | 18 |
| healed | 18 | 11 |
| healing | 66 | 21 |
| heals | 17 | 13 |
| health | 6 | 4 |
| healthcare | 2 | 2 |
| healthier | 7 | 7 |
| healthy | 33 | 15 |
| heart | 7 | 4 |
| heavy | 11 | 8 |
| heavy-smoking | 1 | 1 |
| Hee-S | 1 | 1 |
| heel | 7 | 3 |
| heels | 2 | 1 |
| Heerwaarden | 1 | 1 |
| height | 34 | 13 |
| heightens | 1 | 1 |
| held | 6 | 5 |
| help | 79 | 23 |
| helped | 2 | 2 |
| helpful | 5 | 5 |
| helping | 2 | 2 |
| helps | 29 | 15 |
| Helsinki | 2 | 2 |
| Hence | 5 | 3 |
| HEP | 3 | 1 |
| heparin | 1 | 1 |
| her | 4 | 3 |
| Here | 12 | 3 |
| Hernigou | 4 | 4 |
| herpes | 1 | 1 |
| heterogeneity | 2 | 2 |
| heterogeneous | 1 | 1 |
| hexapod | 1 | 1 |
| Hg | 1 | 1 |
| Hibiclens | 1 | 1 |
| high | 207 | 39 |
| high-demand | 3 | 3 |
| high-impact | 3 | 3 |
| high-level | 3 | 2 |
| high-volume | 1 | 1 |
| higher | 41 | 18 |
| highest | 5 | 4 |
| highlight | 1 | 1 |
| highlighted | 3 | 2 |
| highly | 14 | 6 |
| Hight | 1 | 1 |
| hiking | 2 | 1 |
| hill | 1 | 1 |
| Hills | 1 | 1 |
| him | 6 | 1 |
| hindfoot | 2 | 1 |
| hinge | 38 | 12 |
| hinged | 12 | 7 |
| hinges | 2 | 1 |
| hip | 31 | 14 |
| hip’s | 2 | 1 |
| hip-knee-ankle | 1 | 1 |
| hip-to-ankle | 3 | 1 |
| hips | 1 | 1 |
| his | 25 | 4 |
| historic | 1 | 1 |
| historical | 1 | 1 |
| Historically | 6 | 6 |
| history | 18 | 12 |
| HKA | 5 | 1 |
| ho-rizontally | 1 | 1 |
| Hoell | 2 | 1 |
| Hofmann | 1 | 1 |
| Hohmann | 3 | 2 |
| Hold | 17 | 11 |
| holding | 2 | 2 |
| holds | 4 | 1 |
| hole | 13 | 6 |
| holes | 6 | 4 |
| Holland | 1 | 1 |
| home | 40 | 13 |
| honest | 1 | 1 |
| honour | 1 | 1 |
| hop | 1 | 1 |
| hope | 2 | 2 |
| hopefully | 1 | 1 |
| hoping | 1 | 1 |
| horizontal | 9 | 5 |
| horizontally | 1 | 1 |
| hormonal | 2 | 1 |
| horn | 5 | 2 |
| horse | 1 | 1 |
| hospital | 59 | 24 |
| hospital's | 1 | 1 |
| hospitalization | 2 | 2 |
| hospitalized | 1 | 1 |
| Hospitals | 6 | 2 |
| hot | 3 | 3 |
| hour | 5 | 2 |
| hours | 24 | 15 |
| hours/day | 1 | 1 |
| house | 1 | 1 |
| Household | 1 | 1 |
| housework | 1 | 1 |
| how | 40 | 16 |
| However | 97 | 27 |
| hrs | 1 | 1 |
| HTO | 513 | 24 |
| HTO’s | 1 | 1 |
| HTO-ACL | 1 | 1 |
| HTO-combined | 1 | 1 |
| HTOs | 28 | 7 |
| Hu | 1 | 1 |
| human | 6 | 4 |
| humans | 1 | 1 |
| hundred | 2 | 1 |
| hurt | 1 | 1 |
| hurts | 1 | 1 |
| hyaluronic | 1 | 1 |
| hydrant | 1 | 1 |
| hydromorphone | 1 | 1 |
| hydroxyapatite | 3 | 3 |
| hygiene | 1 | 1 |
| hypercorrection | 2 | 2 |
| hyperextension | 1 | 1 |
| hypothesis | 1 | 1 |
| hypothesized | 1 | 1 |
| I | 65 | 6 |
| I’ve | 1 | 1 |
| I-shaped | 2 | 1 |
| I. | 1 | 1 |
| i.e | 1 | 1 |
| i.e. | 12 | 6 |
| iatrogenic | 1 | 1 |
| iBalance | 9 | 3 |
| IBM | 1 | 1 |
| ibuprofen | 4 | 4 |
| ICD-10 | 2 | 1 |
| ice | 17 | 9 |
| icing | 3 | 3 |
| ICRS | 5 | 2 |
| idea | 9 | 3 |
| ideal | 25 | 10 |
| Ideally | 3 | 2 |
| identical | 2 | 2 |
| identification | 4 | 4 |
| identified | 16 | 8 |
| identifies | 1 | 1 |
| identify | 8 | 5 |
| Identifying | 5 | 4 |
| ie | 8 | 1 |
| If | 249 | 38 |
| ignore | 1 | 1 |
| ignored | 1 | 1 |
| II | 13 | 5 |
| II-III | 1 | 1 |
| III | 11 | 10 |
| IKDC | 2 | 1 |
| IL | 1 | 1 |
| iliac | 13 | 7 |
| iliopsoas | 1 | 1 |
| Ilizarov | 2 | 1 |
| illiac | 1 | 1 |
| Illinois | 2 | 1 |
| illnesses | 1 | 1 |
| illustrates | 1 | 1 |
| Ilstrup | 2 | 1 |
| im-pairment | 1 | 1 |
| image | 15 | 5 |
| images | 4 | 4 |
| imaginary | 1 | 1 |
| imaginative | 1 | 1 |
| imagine | 2 | 2 |
| imaging | 10 | 7 |
| imbalance | 5 | 3 |
| immediate | 4 | 4 |
| immediately | 13 | 7 |
| imminent | 1 | 1 |
| immobilisation | 1 | 1 |
| immobilization | 5 | 4 |
| Immobilize | 1 | 1 |
| immobilized | 2 | 2 |
| immobilizer | 2 | 2 |
| immune | 3 | 3 |
| impact | 8 | 3 |
| impaction | 1 | 1 |
| impair | 2 | 2 |
| Impaired | 2 | 2 |
| impairment | 2 | 2 |
| impairments | 2 | 1 |
| impede | 1 | 1 |
| imperative | 2 | 2 |
| imperceptibly | 1 | 1 |
| imperfecta | 4 | 1 |
| impingement | 2 | 1 |
| implant | 23 | 8 |
| implantation | 17 | 5 |
| implanted | 7 | 2 |
| implants | 17 | 7 |
| implementation | 1 | 1 |
| implications | 2 | 2 |
| importance | 7 | 6 |
| important | 73 | 24 |
| imposed | 2 | 2 |
| impossibility | 1 | 1 |
| impressing | 1 | 1 |
| improper | 1 | 1 |
| improve | 36 | 21 |
| improved | 39 | 17 |
| improvement | 23 | 12 |
| improvements | 9 | 8 |
| improves | 4 | 4 |
| improving | 10 | 7 |
| in | 1742 | 44 |
| in-clude | 1 | 1 |
| in-cluding | 1 | 1 |
| in-hospital | 1 | 1 |
| in-patient | 1 | 1 |
| in-serted | 1 | 1 |
| in-ternal | 1 | 1 |
| inability | 1 | 1 |
| inasmuch | 1 | 1 |
| inbetween | 1 | 1 |
| Inc. | 1 | 1 |
| inch | 1 | 1 |
| incidence | 14 | 11 |
| incised | 1 | 1 |
| incision | 47 | 24 |
| incisions | 6 | 6 |
| incitements | 1 | 1 |
| inclination | 7 | 5 |
| incline | 1 | 1 |
| include | 41 | 22 |
| included | 11 | 6 |
| includes | 11 | 7 |
| including | 50 | 24 |
| Inclusion | 2 | 2 |
| inclusion/exclusion | 1 | 1 |
| incomplete | 3 | 3 |
| inconsistent | 4 | 2 |
| inconvenience | 1 | 1 |
| incorporated | 2 | 2 |
| incorrect | 1 | 1 |
| incorrectly | 1 | 1 |
| increase | 51 | 20 |
| increased | 62 | 19 |
| increases | 22 | 15 |
| increasing | 22 | 14 |
| increasingly | 2 | 2 |
| incredibly | 1 | 1 |
| indeed | 3 | 2 |
| independent | 11 | 4 |
| index | 9 | 5 |
| indicate | 2 | 1 |
| indicated | 28 | 10 |
| indicates | 2 | 2 |
| indicating | 4 | 3 |
| indication | 21 | 9 |
| indications | 23 | 9 |
| indicator | 2 | 2 |
| indicators | 2 | 1 |
| indices | 2 | 1 |
| indispensable | 1 | 1 |
| indisputable | 1 | 1 |
| individual | 6 | 5 |
| individual’s | 1 | 1 |
| individualized | 3 | 3 |
| individually | 3 | 2 |
| individuals | 6 | 5 |
| Indocid | 1 | 1 |
| induce | 4 | 3 |
| induced | 2 | 1 |
| induces | 2 | 2 |
| industrialization | 1 | 1 |
| ineffective | 2 | 2 |
| inevitable | 2 | 2 |
| inexcusable | 1 | 1 |
| inexperienced | 1 | 1 |
| infected | 20 | 3 |
| infection | 95 | 23 |
| infections | 9 | 7 |
| infectious | 2 | 1 |
| infera | 6 | 3 |
| inferable | 1 | 1 |
| inferior | 8 | 4 |
| inferiorly | 1 | 1 |
| inferomedial | 1 | 1 |
| infiltration | 2 | 1 |
| inflammation | 2 | 2 |
| inflammatory | 9 | 6 |
| influence | 14 | 5 |
| influenced | 1 | 1 |
| influences | 3 | 3 |
| inform | 3 | 2 |
| information | 17 | 9 |
| informed | 3 | 3 |
| infra | 1 | 1 |
| inherent | 2 | 2 |
| inhibition | 1 | 1 |
| initial | 17 | 8 |
| initially | 14 | 8 |
| initials | 1 | 1 |
| initiate | 1 | 1 |
| initiated | 2 | 2 |
| inject | 1 | 1 |
| injectable | 1 | 1 |
| injecting | 2 | 1 |
| injection | 4 | 3 |
| injections | 4 | 4 |
| injure | 1 | 1 |
| injuries | 24 | 9 |
| Injury | 46 | 21 |
| inner | 24 | 11 |
| inner/medial | 1 | 1 |
| innovative | 2 | 1 |
| inpatient | 4 | 3 |
| input | 1 | 1 |
| inquiries | 1 | 1 |
| Insall-Salvati | 3 | 3 |
| insert | 7 | 4 |
| inserted | 39 | 12 |
| inserting | 6 | 5 |
| insertion | 12 | 8 |
| inserts | 2 | 2 |
| inside | 19 | 11 |
| insight | 1 | 1 |
| insights | 3 | 1 |
| insist | 1 | 1 |
| insoles | 1 | 1 |
| inspect | 1 | 1 |
| instability | 91 | 21 |
| Install | 2 | 1 |
| instance | 7 | 3 |
| Instead | 13 | 8 |
| Institute | 1 | 1 |
| institution | 1 | 1 |
| institutional | 2 | 2 |
| institutions | 1 | 1 |
| instruct | 1 | 1 |
| instructed | 7 | 4 |
| instruction | 6 | 2 |
| instructions | 13 | 9 |
| instrument | 1 | 1 |
| instrumentation | 5 | 4 |
| instruments | 4 | 3 |
| insufficiency | 12 | 5 |
| insufficient | 8 | 4 |
| insurance | 4 | 3 |
| intact | 22 | 14 |
| intake | 2 | 2 |
| integrate | 2 | 2 |
| integrated | 1 | 1 |
| Integration | 1 | 1 |
| integrity | 2 | 2 |
| intended | 7 | 5 |
| intense | 1 | 1 |
| intensifier | 1 | 1 |
| intensity | 4 | 3 |
| intensive | 1 | 1 |
| intent | 1 | 1 |
| intentionally | 1 | 1 |
| interaction | 2 | 2 |
| interactions | 1 | 1 |
| interest | 9 | 6 |
| interested | 1 | 1 |
| Interestingly | 1 | 1 |
| interfere | 2 | 2 |
| interference | 1 | 1 |
| Interferential | 1 | 1 |
| interferes | 1 | 1 |
| interfragmentary | 1 | 1 |
| interindividual | 1 | 1 |
| intermedia | 2 | 1 |
| intermediate | 1 | 1 |
| intermediate-term | 2 | 1 |
| intermittently | 2 | 2 |
| internal | 16 | 9 |
| international | 9 | 6 |
| interquartile | 1 | 1 |
| intersect | 1 | 1 |
| interval | 6 | 2 |
| intervals | 4 | 3 |
| intervention | 6 | 4 |
| interventions | 1 | 1 |
| into | 78 | 21 |
| intra-articular | 8 | 6 |
| intra-operative | 1 | 1 |
| intra-operatively | 1 | 1 |
| intraarticular | 5 | 3 |
| intramedullary | 4 | 1 |
| intraoperative | 3 | 2 |
| Intraoperatively | 4 | 4 |
| intraosseous | 1 | 1 |
| intravenous | 8 | 3 |
| intravenously | 2 | 2 |
| intrinsic | 1 | 1 |
| introduce | 2 | 2 |
| introduced | 11 | 8 |
| introducing | 1 | 1 |
| introduction | 3 | 1 |
| invasiveness | 1 | 1 |
| invented | 2 | 1 |
| inventors | 1 | 1 |
| inverse | 5 | 3 |
| inversely | 1 | 1 |
| Investigate | 8 | 2 |
| investigated | 10 | 6 |
| investigating | 1 | 1 |
| investigation | 2 | 2 |
| investigations | 1 | 1 |
| inveterate | 1 | 1 |
| invisible | 1 | 1 |
| invited | 1 | 1 |
| involve | 6 | 6 |
| involved | 12 | 8 |
| involvement | 1 | 1 |
| involves | 14 | 8 |
| involving | 7 | 6 |
| inward | 3 | 2 |
| ipsilateral | 2 | 2 |
| irreversible | 1 | 1 |
| irritation | 6 | 5 |
| is | 1629 | 45 |
| ISAKOS | 4 | 3 |
| Island | 4 | 1 |
| isn’t | 2 | 1 |
| isolated | 29 | 14 |
| isolation | 1 | 1 |
| isometric | 1 | 1 |
| isometrics | 1 | 1 |
| Israel | 1 | 1 |
| issue | 7 | 4 |
| issued | 1 | 1 |
| issues | 8 | 4 |
| It | 461 | 40 |
| it’s | 5 | 2 |
| Italian | 1 | 1 |
| Italy | 1 | 1 |
| items | 1 | 1 |
| its | 46 | 18 |
| itself | 8 | 4 |
| IV | 3 | 3 |
| ivy | 2 | 2 |
| Jackson | 4 | 4 |
| Jacob | 1 | 1 |
| Jakob | 2 | 2 |
| January | 1 | 1 |
| Japan | 1 | 1 |
| jeopardize | 3 | 2 |
| jewelry | 1 | 1 |
| jig | 2 | 1 |
| job | 11 | 5 |
| jobs | 2 | 2 |
| jogging | 3 | 2 |
| joint | 356 | 40 |
| joint-line | 1 | 1 |
| joint-preserving | 2 | 2 |
| joints | 16 | 8 |
| Jonathan | 1 | 1 |
| journey | 1 | 1 |
| JP | 1 | 1 |
| judgement | 1 | 1 |
| judgment | 1 | 1 |
| juice | 1 | 1 |
| July | 1 | 1 |
| jumping | 4 | 4 |
| junction | 1 | 1 |
| June | 2 | 2 |
| just | 64 | 18 |
| K-wire | 1 | 1 |
| K-wires | 5 | 3 |
| Kachooei | 1 | 1 |
| Kahlenberg | 1 | 1 |
| KAM | 8 | 1 |
| Karatosun | 1 | 1 |
| Kazakhstan | 1 | 1 |
| Kaze | 1 | 1 |
| KCF | 5 | 1 |
| KCI | 1 | 1 |
| keels | 1 | 1 |
| keen | 1 | 1 |
| Keenan | 2 | 2 |
| Keep | 17 | 9 |
| keeping | 9 | 5 |
| keeps | 1 | 1 |
| Kellgren | 1 | 1 |
| Kellgren-Lawrence | 6 | 4 |
| KellgrenLawrence | 1 | 1 |
| kept | 4 | 4 |
| key | 12 | 10 |
| key-hole | 2 | 1 |
| KFM | 1 | 1 |
| kg | 1 | 1 |
| kg/m | 1 | 1 |
| kg/m2 | 1 | 1 |
| killers | 1 | 1 |
| Kim | 2 | 2 |
| kind | 6 | 5 |
| kinematic | 1 | 1 |
| kinematical | 2 | 1 |
| kinematics | 13 | 4 |
| kinetics | 1 | 1 |
| Kirgizstan | 1 | 1 |
| Kissing | 1 | 1 |
| kitchen | 2 | 1 |
| knee | 1229 | 44 |
| knee's | 9 | 3 |
| knee-cap | 1 | 1 |
| knee.2 | 1 | 1 |
| kneecap | 16 | 9 |
| kneed | 1 | 1 |
| kneed/valgus | 1 | 1 |
| kneeling | 2 | 2 |
| knees | 76 | 19 |
| knock | 5 | 3 |
| knock-kneed | 19 | 8 |
| knock-knees | 1 | 1 |
| knocked | 1 | 1 |
| know | 9 | 4 |
| knowledge | 7 | 2 |
| known | 25 | 13 |
| Koenig | 1 | 1 |
| KOOS | 2 | 1 |
| Korea | 2 | 2 |
| Koshimo | 1 | 1 |
| Koshino | 2 | 1 |
| KSS | 6 | 1 |
| Kwon | 1 | 1 |
| L | 3 | 2 |
| L-shape | 1 | 1 |
| L-shaped | 2 | 2 |
| lab | 1 | 1 |
| labeled | 1 | 1 |
| laboratory | 2 | 2 |
| labourer | 1 | 1 |
| Lachman | 1 | 1 |
| lack | 5 | 4 |
| lacking | 2 | 2 |
| lady | 2 | 1 |
| lag | 3 | 3 |
| laid | 1 | 1 |
| laminar | 5 | 3 |
| landmarks | 2 | 2 |
| Lane | 1 | 1 |
| Laprade | 1 | 1 |
| large | 24 | 12 |
| largely | 5 | 2 |
| larger | 6 | 3 |
| largest | 1 | 1 |
| Lash | 1 | 1 |
| last | 22 | 9 |
| lasted | 1 | 1 |
| lasting | 1 | 1 |
| Lastly | 7 | 3 |
| lasts | 6 | 5 |
| latae | 1 | 1 |
| late | 6 | 6 |
| Lately | 1 | 1 |
| latent | 1 | 1 |
| later | 26 | 13 |
| lateral | 288 | 29 |
| lateral-hinge | 1 | 1 |
| lateral-most | 1 | 1 |
| lateral/postero-lateral | 1 | 1 |
| laterally | 13 | 7 |
| latest | 1 | 1 |
| latter | 5 | 3 |
| Latterman | 1 | 1 |
| launch | 1 | 1 |
| Lawrence | 1 | 1 |
| laxative | 1 | 1 |
| laxity | 13 | 7 |
| layer | 3 | 3 |
| layers | 1 | 1 |
| layout | 1 | 1 |
| LCP | 1 | 1 |
| LCW | 25 | 2 |
| lead | 19 | 12 |
| leading | 6 | 6 |
| leads | 12 | 10 |
| leaflet | 2 | 2 |
| leaking | 1 | 1 |
| learn | 1 | 1 |
| learning | 3 | 2 |
| least | 46 | 19 |
| leave | 7 | 4 |
| leaves | 2 | 2 |
| leaving | 5 | 5 |
| led | 4 | 4 |
| Lee | 1 | 1 |
| left | 29 | 12 |
| leg | 176 | 35 |
| leg-straightening | 1 | 1 |
| legged | 2 | 2 |
| legs | 24 | 9 |
| Leitch | 1 | 1 |
| length | 24 | 11 |
| lengthening | 9 | 2 |
| lengthens | 1 | 1 |
| lengths | 1 | 1 |
| lengthy | 1 | 1 |
| Lerner | 1 | 1 |
| lesion | 11 | 5 |
| lesions | 15 | 7 |
| less | 67 | 21 |
| less-invasive | 1 | 1 |
| lessen | 1 | 1 |
| lesson | 1 | 1 |
| let | 2 | 2 |
| Let’s | 1 | 1 |
| level | 40 | 17 |
| levels | 8 | 5 |
| levered | 1 | 1 |
| levering | 1 | 1 |
| levofloxacin | 1 | 1 |
| lies | 4 | 3 |
| life | 14 | 11 |
| lifestyle | 11 | 8 |
| lift | 2 | 2 |
| lifting | 2 | 2 |
| lifts | 1 | 1 |
| ligament | 101 | 17 |
| ligamentitis | 1 | 1 |
| ligamentous | 31 | 10 |
| ligaments | 23 | 12 |
| light | 12 | 8 |
| lighter | 2 | 2 |
| like | 42 | 8 |
| liked | 1 | 1 |
| likelihood | 4 | 3 |
| likely | 26 | 16 |
| Likewise | 1 | 1 |
| limb | 77 | 15 |
| limbs | 6 | 4 |
| limit | 9 | 6 |
| limitation | 5 | 4 |
| limitations | 6 | 5 |
| limited | 13 | 11 |
| limiting | 5 | 3 |
| limits | 2 | 2 |
| line | 138 | 21 |
| linear | 1 | 1 |
| Lines | 10 | 6 |
| linked | 1 | 1 |
| liquid | 1 | 1 |
| LISS | 1 | 1 |
| list | 1 | 1 |
| listed | 3 | 3 |
| literally | 4 | 4 |
| literature | 18 | 8 |
| little | 9 | 6 |
| live | 3 | 2 |
| living | 2 | 1 |
| LMR | 1 | 1 |
| load | 39 | 16 |
| load-bearing | 3 | 3 |
| load-to-failure | 2 | 1 |
| loaded | 1 | 1 |
| loading | 23 | 7 |
| loads | 5 | 4 |
| loads/tension | 1 | 1 |
| Lobenhoffer | 1 | 1 |
| local | 16 | 5 |
| localised | 5 | 1 |
| localized | 3 | 3 |
| located | 7 | 6 |
| locates | 1 | 1 |
| locating | 1 | 1 |
| location | 8 | 7 |
| lock | 7 | 3 |
| locked | 15 | 7 |
| locking | 46 | 17 |
| locking/unlocking | 1 | 1 |
| locks | 1 | 1 |
| logical | 1 | 1 |
| Logically | 1 | 1 |
| long | 64 | 22 |
| long-acting | 1 | 1 |
| long-bone | 1 | 1 |
| long-leg | 3 | 1 |
| long-or | 1 | 1 |
| long-stem | 1 | 1 |
| long-term | 31 | 13 |
| longer | 23 | 14 |
| longer-term | 1 | 1 |
| longer.3 | 1 | 1 |
| longevity | 1 | 1 |
| longitudinal | 5 | 4 |
| longterm | 1 | 1 |
| look | 17 | 5 |
| looked | 4 | 2 |
| looking | 13 | 3 |
| looks | 5 | 1 |
| loose | 5 | 5 |
| loosen | 2 | 2 |
| loosening | 8 | 5 |
| Los | 1 | 1 |
| lose | 5 | 3 |
| loses | 1 | 1 |
| loss | 64 | 21 |
| lost | 7 | 3 |
| lot | 19 | 3 |
| lots | 2 | 2 |
| loved | 1 | 1 |
| lovely | 1 | 1 |
| low | 23 | 12 |
| low-dose | 1 | 1 |
| low-grade | 4 | 1 |
| low-impact | 2 | 1 |
| low-molecular | 1 | 1 |
| low-to-extreme | 1 | 1 |
| lower | 95 | 24 |
| lower-limb | 1 | 1 |
| lowered | 4 | 4 |
| lowering | 1 | 1 |
| lowest | 2 | 1 |
| LS | 1 | 1 |
| Ltd. | 2 | 2 |
| lucky | 1 | 1 |
| Luites | 2 | 2 |
| lumbar | 2 | 1 |
| lumber | 1 | 1 |
| lumpy | 1 | 1 |
| Lund | 1 | 1 |
| lung | 1 | 1 |
| Lunge | 2 | 1 |
| lungs | 1 | 1 |
| Luxembourg | 1 | 1 |
| luxury | 1 | 1 |
| Lying | 5 | 2 |
| Lyrica | 1 | 1 |
| lyshlom | 1 | 1 |
| Lysholm | 2 | 2 |
| lysis | 1 | 1 |
| M | 1 | 1 |
| M. | 1 | 1 |
| M17 | 2 | 1 |
| Machine | 9 | 4 |
| machines | 3 | 2 |
| macroscopic | 3 | 2 |
| MAD | 7 | 1 |
| made | 59 | 22 |
| Magnetic | 4 | 4 |
| magnitudes | 1 | 1 |
| main | 19 | 7 |
| mainly | 9 | 6 |
| Maintain | 21 | 15 |
| maintained | 11 | 8 |
| maintaining | 2 | 2 |
| maintains | 2 | 1 |
| maintenance | 4 | 3 |
| major | 17 | 7 |
| majority | 13 | 6 |
| make | 45 | 19 |
| makes | 14 | 7 |
| Making | 15 | 8 |
| mal-alignment | 4 | 1 |
| mal-union | 1 | 1 |
| mala-lignment | 1 | 1 |
| malaligned | 6 | 3 |
| malalignment | 68 | 19 |
| male | 4 | 2 |
| males | 2 | 2 |
| malnutrition | 1 | 1 |
| malposition | 1 | 1 |
| maltracking | 1 | 1 |
| man | 1 | 1 |
| manage | 11 | 6 |
| manageable | 2 | 2 |
| managed | 5 | 5 |
| management | 8 | 7 |
| managing | 2 | 2 |
| mandatory | 6 | 6 |
| manifestations | 1 | 1 |
| manipulate | 1 | 1 |
| manipulated | 1 | 1 |
| manipulation | 2 | 2 |
| Mann-Whitney | 1 | 1 |
| manner | 3 | 3 |
| manoeuvres | 1 | 1 |
| manual | 2 | 2 |
| manufactured | 1 | 1 |
| manufactures | 1 | 1 |
| Many | 49 | 23 |
| map | 1 | 1 |
| Marcaine | 1 | 1 |
| March | 1 | 1 |
| margin | 5 | 4 |
| margins | 1 | 1 |
| mark | 5 | 3 |
| marked | 9 | 7 |
| markedly | 2 | 2 |
| marker-based | 2 | 1 |
| markers | 1 | 1 |
| market | 1 | 1 |
| marking | 1 | 1 |
| Marriott | 1 | 1 |
| marrow | 3 | 2 |
| Martay | 1 | 1 |
| Martin | 1 | 1 |
| mask | 1 | 1 |
| mass | 5 | 4 |
| massive | 1 | 1 |
| massively | 1 | 1 |
| Masterclass | 1 | 1 |
| match | 2 | 1 |
| matches | 3 | 3 |
| matching | 2 | 2 |
| material | 6 | 6 |
| Materialise | 2 | 1 |
| materials | 3 | 3 |
| matrix | 1 | 1 |
| matrix-induced | 1 | 1 |
| mats | 1 | 1 |
| matter | 2 | 2 |
| matters | 1 | 1 |
| Matthews | 1 | 1 |
| maxillofacial | 1 | 1 |
| maximal | 2 | 2 |
| maximize | 1 | 1 |
| maximizing | 1 | 1 |
| maximum | 4 | 4 |
| maximus | 1 | 1 |
| may | 289 | 42 |
| MB | 2 | 1 |
| McGill | 4 | 1 |
| McHale | 1 | 1 |
| MCL | 7 | 3 |
| McMiniman | 1 | 1 |
| McNamara | 1 | 1 |
| MD | 2 | 1 |
| me | 3 | 1 |
| meal | 1 | 1 |
| meals | 4 | 2 |
| mean | 31 | 10 |
| meaning | 7 | 5 |
| means | 15 | 10 |
| meant | 4 | 3 |
| measure | 4 | 3 |
| measured | 25 | 11 |
| measurement | 2 | 2 |
| Measurements | 6 | 4 |
| measures | 7 | 6 |
| measuring | 2 | 2 |
| mechanical | 109 | 20 |
| mechanics | 7 | 1 |
| mechanism | 1 | 1 |
| mechanotherapy | 1 | 1 |
| medial | 400 | 32 |
| medial-based | 2 | 1 |
| medial-lateral | 7 | 1 |
| medially | 6 | 5 |
| median | 4 | 4 |
| medical | 44 | 16 |
| medication | 17 | 9 |
| medications | 29 | 14 |
| medicinal | 2 | 1 |
| Medicine | 16 | 8 |
| medium | 1 | 1 |
| medium-and | 1 | 1 |
| medium-to | 1 | 1 |
| medium-to-large-sized | 2 | 1 |
| medullary | 1 | 1 |
| meet | 6 | 3 |
| meeting | 1 | 1 |
| meets | 1 | 1 |
| meloxicam | 1 | 1 |
| member | 1 | 1 |
| membrane | 1 | 1 |
| men | 6 | 4 |
| Meng | 1 | 1 |
| meniscal | 32 | 10 |
| meniscectomies | 1 | 1 |
| meniscectomized | 1 | 1 |
| meniscectomy | 7 | 6 |
| menisci | 9 | 6 |
| meniscus | 35 | 15 |
| menisectomy | 1 | 1 |
| mention | 3 | 2 |
| mentioned | 9 | 8 |
| mepore | 4 | 1 |
| merges | 1 | 1 |
| mesenchymal | 2 | 1 |
| met | 3 | 3 |
| meta-analysis | 2 | 1 |
| metal | 27 | 13 |
| metallosis | 1 | 1 |
| metalwork | 1 | 1 |
| metaphyseal | 10 | 7 |
| metaphysis | 6 | 5 |
| method | 69 | 15 |
| methodologies | 1 | 1 |
| methodology | 1 | 1 |
| methods | 21 | 10 |
| methotrexate | 1 | 1 |
| methylprednisolone | 1 | 1 |
| meticulous | 1 | 1 |
| metric | 1 | 1 |
| metrics | 2 | 1 |
| mFTA | 1 | 1 |
| mg | 1 | 1 |
| mg/day | 1 | 1 |
| mg/week | 1 | 1 |
| micro-alignment | 1 | 1 |
| micro-motion | 1 | 1 |
| microbial | 1 | 1 |
| microbiological | 1 | 1 |
| microfracture | 18 | 8 |
| microfractures | 1 | 1 |
| microscopic | 1 | 1 |
| Microsoft | 1 | 1 |
| mid-30s | 1 | 1 |
| mid-and | 1 | 1 |
| mid-diaphysis | 1 | 1 |
| mid-term | 1 | 1 |
| middle | 9 | 7 |
| middle-aged | 4 | 3 |
| midfoot | 2 | 1 |
| midline | 3 | 2 |
| midnight | 2 | 2 |
| midpoint | 4 | 3 |
| midway | 3 | 3 |
| might | 13 | 8 |
| Migration | 2 | 1 |
| Mikulicz-Radecki | 1 | 1 |
| mild | 6 | 5 |
| miles | 2 | 1 |
| millimeters | 3 | 2 |
| millimetre | 3 | 1 |
| millimetres | 2 | 1 |
| millions | 2 | 2 |
| Mimics | 5 | 5 |
| min. | 1 | 1 |
| mind | 3 | 3 |
| mini-open | 1 | 1 |
| mini-tramp | 1 | 1 |
| Miniacci | 1 | 1 |
| Miniaci | 4 | 4 |
| minimal | 6 | 6 |
| minimal/limited | 1 | 1 |
| minimise | 3 | 3 |
| minimised | 1 | 1 |
| minimize | 4 | 3 |
| minimized | 1 | 1 |
| minimum | 9 | 4 |
| minor | 8 | 6 |
| minus | 1 | 1 |
| minutes | 14 | 9 |
| mirror | 1 | 1 |
| misalignment | 2 | 1 |
| misclassified | 1 | 1 |
| misdiagnosed | 1 | 1 |
| misinterpreted | 1 | 1 |
| misnomer | 1 | 1 |
| miss | 1 | 1 |
| missed | 2 | 2 |
| missing | 4 | 2 |
| mistake | 1 | 1 |
| mistakenly | 1 | 1 |
| mistargeted | 1 | 1 |
| mixed | 3 | 3 |
| mLDFA | 2 | 1 |
| mm | 48 | 13 |
| mm. | 3 | 3 |
| mMPTA | 2 | 1 |
| mms | 1 | 1 |
| mobile | 3 | 3 |
| mobile-bearing | 1 | 1 |
| mobilisation | 1 | 1 |
| mobilise | 3 | 3 |
| mobilised | 1 | 1 |
| mobilising | 2 | 1 |
| mobility | 17 | 9 |
| mobility/transfers | 1 | 1 |
| mobilization | 6 | 4 |
| mobilizations | 1 | 1 |
| mobilized | 1 | 1 |
| modalities | 1 | 1 |
| modality | 1 | 1 |
| model | 8 | 4 |
| modeling | 1 | 1 |
| modelling | 7 | 1 |
| models | 5 | 3 |
| moderate | 12 | 9 |
| moderate-to-severe | 1 | 1 |
| moderately | 3 | 3 |
| modern | 8 | 5 |
| modification | 4 | 3 |
| modifications | 6 | 4 |
| modified | 7 | 6 |
| modify | 4 | 3 |
| modifying | 2 | 2 |
| molecular | 1 | 1 |
| moment | 15 | 6 |
| moments | 4 | 2 |
| Monday | 1 | 1 |
| monitor | 5 | 4 |
| monitored | 6 | 4 |
| monitoring | 2 | 2 |
| monocompartmental | 1 | 1 |
| month | 3 | 3 |
| monthly | 1 | 1 |
| months | 87 | 29 |
| moon | 2 | 1 |
| Morbidity | 3 | 2 |
| more | 227 | 34 |
| more,1-3 | 1 | 1 |
| Moreover | 11 | 6 |
| morning | 2 | 2 |
| morphological | 2 | 2 |
| morphotype | 1 | 1 |
| mosaicplasty | 1 | 1 |
| Moscow | 1 | 1 |
| most | 172 | 38 |
| mostly | 3 | 3 |
| motion | 69 | 26 |
| motion-capture | 1 | 1 |
| motions | 1 | 1 |
| motivated | 4 | 3 |
| motivation | 5 | 3 |
| Motor | 6 | 2 |
| move | 31 | 9 |
| moved | 9 | 4 |
| movement | 18 | 6 |
| movements | 2 | 2 |
| movers | 1 | 1 |
| moves | 3 | 3 |
| moving | 8 | 5 |
| MOW | 28 | 2 |
| MOWHTO | 15 | 2 |
| MPTA | 1 | 1 |
| Mr | 1 | 1 |
| Mr. | 3 | 1 |
| MRI | 24 | 12 |
| MT | 3 | 1 |
| MTF | 1 | 1 |
| much | 68 | 15 |
| Mukhanov | 1 | 1 |
| multi-vitamins | 1 | 1 |
| multiaxial | 1 | 1 |
| multibody | 2 | 1 |
| multicenter | 1 | 1 |
| multidirectional | 1 | 1 |
| multidisciplinary | 1 | 1 |
| multilevel | 3 | 1 |
| multiligamentous | 1 | 1 |
| Multiobjective | 1 | 1 |
| multiplanar | 1 | 1 |
| multiplane | 1 | 1 |
| multiple | 12 | 7 |
| multiply | 1 | 1 |
| multiresistance | 1 | 1 |
| Munich | 1 | 1 |
| muscle | 26 | 12 |
| muscles | 24 | 11 |
| muscular | 1 | 1 |
| Musculoskeletal | 15 | 3 |
| must | 46 | 17 |
| mutual | 1 | 1 |
| my | 14 | 1 |
| Myers | 3 | 1 |
| myotomes | 1 | 1 |
| Müller | 1 | 1 |
| N | 12 | 3 |
| N. | 1 | 1 |
| nail | 2 | 1 |
| nailing | 1 | 1 |
| nails | 4 | 1 |
| Nakamura | 1 | 1 |
| Nakayama | 1 | 1 |
| named | 2 | 1 |
| names | 1 | 1 |
| Naprosyn | 1 | 1 |
| Naproxen | 1 | 1 |
| narcotic | 7 | 3 |
| narcotics | 1 | 1 |
| narrow | 3 | 3 |
| narrower | 2 | 1 |
| narrowing | 10 | 6 |
| narrows | 2 | 2 |
| Nasonova | 1 | 1 |
| nation | 2 | 1 |
| national | 5 | 1 |
| native | 2 | 2 |
| natural | 8 | 7 |
| naturally | 3 | 2 |
| nature | 3 | 3 |
| Naudie | 3 | 3 |
| Nausea | 2 | 2 |
| navigate | 1 | 1 |
| navigation | 12 | 6 |
| near | 8 | 7 |
| near-normal | 2 | 2 |
| nearby | 1 | 1 |
| nearest | 8 | 2 |
| nearing | 1 | 1 |
| Nearly | 2 | 2 |
| necessarily | 1 | 1 |
| necessary | 25 | 22 |
| necessitate | 5 | 3 |
| necessitates | 3 | 3 |
| neck | 1 | 1 |
| necrosis | 6 | 5 |
| necrotic | 2 | 1 |
| need | 102 | 33 |
| needed | 24 | 12 |
| needlessly | 1 | 1 |
| needs | 32 | 11 |
| negative | 11 | 8 |
| negatively | 3 | 3 |
| neighbours | 1 | 1 |
| neither | 2 | 2 |
| Nelissen | 2 | 1 |
| nerve | 36 | 17 |
| nerves | 14 | 7 |
| nervous | 1 | 1 |
| network | 1 | 1 |
| neurologic | 1 | 1 |
| neurological | 1 | 1 |
| neuromuscular | 1 | 1 |
| Neurontin | 1 | 1 |
| neuropathy | 1 | 1 |
| neurovascular | 14 | 6 |
| neutral | 11 | 4 |
| never | 3 | 2 |
| Nevertheless | 11 | 6 |
| new | 42 | 16 |
| newly | 5 | 4 |
| NexGen | 1 | 1 |
| next | 21 | 10 |
| NGK59 | 2 | 1 |
| nicotine | 4 | 4 |
| Niemeyer | 2 | 2 |
| niggle | 1 | 1 |
| night | 6 | 5 |
| nights | 3 | 2 |
| Nijnmegen | 1 | 1 |
| nine | 5 | 3 |
| Ninety-one | 1 | 1 |
| No | 117 | 24 |
| no-hole | 1 | 1 |
| no-one | 1 | 1 |
| Non | 6 | 4 |
| non-bony | 1 | 1 |
| Non-contact | 1 | 1 |
| non-locking | 6 | 2 |
| non-operated | 3 | 2 |
| non-operative | 8 | 4 |
| Non-parametric | 1 | 1 |
| non-randomized | 1 | 1 |
| Non-slip | 1 | 1 |
| non-smoker | 3 | 3 |
| Non-steroidal | 1 | 1 |
| non-surgical | 1 | 1 |
| non-traumatic | 2 | 1 |
| non-union | 20 | 11 |
| non-unions | 1 | 1 |
| non-weight | 5 | 5 |
| non-weight-bearing | 5 | 4 |
| non-weightbearing | 2 | 1 |
| nonarticulating | 3 | 1 |
| noncomparative | 1 | 1 |
| none | 5 | 3 |
| none-weight | 1 | 1 |
| Nonetheless | 3 | 2 |
| noninvasive | 2 | 1 |
| nonlocking | 1 | 1 |
| nonnegligible | 1 | 1 |
| nonoperated | 1 | 1 |
| nonoperative | 2 | 2 |
| nonparametric | 1 | 1 |
| nonsteroidal | 4 | 4 |
| nonsurgical | 1 | 1 |
| nontender | 1 | 1 |
| nonunion | 41 | 8 |
| nonunions | 1 | 1 |
| nor | 3 | 3 |
| normal | 62 | 21 |
| normality | 1 | 1 |
| normalize | 1 | 1 |
| Normalized | 3 | 3 |
| normally | 9 | 6 |
| not | 272 | 32 |
| notable | 1 | 1 |
| note | 10 | 7 |
| noted | 18 | 8 |
| noteworthy | 1 | 1 |
| nothing | 7 | 2 |
| notice | 3 | 3 |
| noticeable | 3 | 3 |
| noticeably | 1 | 1 |
| noticed | 2 | 2 |
| notify | 2 | 2 |
| nourishment | 1 | 1 |
| novel | 5 | 3 |
| now | 36 | 10 |
| Nowadays | 10 | 6 |
| Noyes | 3 | 2 |
| NSAIDs | 3 | 3 |
| nuisance | 1 | 1 |
| numb | 4 | 4 |
| numbed | 1 | 1 |
| number | 25 | 11 |
| numbers | 3 | 2 |
| numbness | 8 | 5 |
| numbs | 1 | 1 |
| Numeric | 2 | 1 |
| numerical | 1 | 1 |
| numerous | 3 | 2 |
| Nurofen | 1 | 1 |
| nurse | 8 | 5 |
| nurses | 3 | 3 |
| nursing | 2 | 1 |
| NYC | 1 | 1 |
| O-legs | 1 | 1 |
| OA | 112 | 7 |
| obese | 7 | 5 |
| obesity | 6 | 5 |
| objective | 11 | 3 |
| objectives | 1 | 1 |
| obligatory | 1 | 1 |
| oblique | 6 | 3 |
| obliquely | 5 | 3 |
| obliquity | 9 | 4 |
| Observation | 4 | 4 |
| observational | 1 | 1 |
| observations | 2 | 2 |
| observed | 22 | 11 |
| observing | 1 | 1 |
| obsolete | 1 | 1 |
| obtain | 11 | 8 |
| obtained | 16 | 12 |
| obtaining | 1 | 1 |
| obtains | 1 | 1 |
| obvious | 1 | 1 |
| obviously | 4 | 1 |
| occasional | 1 | 1 |
| Occasionally | 9 | 7 |
| occupational | 1 | 1 |
| occupied | 1 | 1 |
| occur | 26 | 14 |
| occurred | 14 | 9 |
| occurrence | 2 | 1 |
| occurrences | 1 | 1 |
| occurring | 1 | 1 |
| occurs | 12 | 7 |
| odd | 1 | 1 |
| ODSCP | 2 | 2 |
| of | 3202 | 45 |
| off | 54 | 19 |
| off-load | 1 | 1 |
| offer | 9 | 4 |
| offering | 2 | 2 |
| offers | 4 | 3 |
| office | 9 | 8 |
| offload | 4 | 1 |
| offloaded | 2 | 1 |
| offloader | 1 | 1 |
| offloading | 3 | 2 |
| offset | 2 | 2 |
| often | 45 | 20 |
| oftentimes | 1 | 1 |
| Oh | 1 | 1 |
| OhtoFix | 1 | 1 |
| Ohtomedical | 1 | 1 |
| OK | 1 | 1 |
| old | 18 | 11 |
| older | 18 | 12 |
| Ollier | 1 | 1 |
| omitted | 1 | 1 |
| on | 612 | 44 |
| once | 42 | 13 |
| one | 161 | 39 |
| one’s | 1 | 1 |
| one-leg | 1 | 1 |
| One-off | 1 | 1 |
| one-or | 1 | 1 |
| one-sided | 4 | 1 |
| one-stage | 1 | 1 |
| one-third | 2 | 2 |
| ones | 3 | 2 |
| ongoing | 8 | 4 |
| only | 82 | 24 |
| Onodera | 2 | 2 |
| onset | 2 | 2 |
| onto | 13 | 8 |
| onwards | 2 | 2 |
| oozing | 1 | 1 |
| op | 1 | 1 |
| op. | 2 | 1 |
| open | 57 | 19 |
| open-wedge | 25 | 4 |
| opened | 17 | 10 |
| opening | 109 | 25 |
| opening-wedge | 17 | 5 |
| opens | 4 | 3 |
| OpenSim | 1 | 1 |
| openwedge | 2 | 1 |
| operaion | 1 | 1 |
| operate | 1 | 1 |
| operated | 33 | 18 |
| operating | 16 | 10 |
| operation | 120 | 23 |
| operations | 12 | 4 |
| operative | 16 | 7 |
| operatively | 1 | 1 |
| opinion | 2 | 2 |
| opinions | 2 | 1 |
| opioids | 3 | 1 |
| opposed | 4 | 3 |
| opposite | 15 | 8 |
| opt | 1 | 1 |
| optimal | 15 | 9 |
| optimally | 1 | 1 |
| optimization | 2 | 2 |
| optimize | 2 | 2 |
| optimized | 1 | 1 |
| optimizes | 2 | 2 |
| option | 28 | 14 |
| Optional | 2 | 2 |
| options | 18 | 9 |
| or | 680 | 44 |
| oral | 12 | 7 |
| orally | 1 | 1 |
| order | 33 | 21 |
| ordered | 1 | 1 |
| orders | 1 | 1 |
| ordinary | 1 | 1 |
| organise | 1 | 1 |
| organised | 1 | 1 |
| organisms | 1 | 1 |
| orientation | 1 | 1 |
| origin | 8 | 1 |
| original | 3 | 2 |
| originally | 5 | 4 |
| Orme | 4 | 1 |
| Ortho-SUV | 4 | 1 |
| orthopaedic | 11 | 7 |
| Orthopaedics | 2 | 1 |
| orthopedic | 25 | 12 |
| orthopedist | 3 | 1 |
| orthopedists | 1 | 1 |
| orthoses | 2 | 1 |
| orthosis | 2 | 2 |
| Orthotics | 4 | 1 |
| os-teotomy | 1 | 1 |
| oscillating | 10 | 10 |
| oseoconductivity | 2 | 2 |
| OSF | 1 | 1 |
| ossification | 3 | 3 |
| osteoarthritic | 6 | 4 |
| osteoarthritis | 152 | 35 |
| osteochondral | 15 | 4 |
| osteochondritis | 5 | 5 |
| osteoconductive | 4 | 2 |
| osteogenesis | 5 | 1 |
| osteogenic | 1 | 1 |
| osteoinductive | 2 | 2 |
| Osteomed | 1 | 1 |
| osteonecrosis | 7 | 6 |
| osteophytes | 2 | 2 |
| osteoporosis | 1 | 1 |
| osteosynthesic | 1 | 1 |
| osteosynthesis | 3 | 1 |
| osteotemy | 1 | 1 |
| osteoto-mies | 1 | 1 |
| osteoto-my | 1 | 1 |
| osteotome | 11 | 6 |
| osteotomes | 5 | 4 |
| osteotomical | 1 | 1 |
| osteotomies | 84 | 19 |
| osteotomized | 1 | 1 |
| osteotomy | 882 | 44 |
| ostetomy | 1 | 1 |
| ostetotomy | 1 | 1 |
| ostotomy | 1 | 1 |
| other | 128 | 30 |
| others | 11 | 7 |
| otherwise | 2 | 2 |
| ottoman | 1 | 1 |
| our | 67 | 11 |
| Oussedik | 1 | 1 |
| out | 95 | 25 |
| Out-patient | 1 | 1 |
| outcome | 36 | 17 |
| outcomes | 46 | 13 |
| outdoor | 1 | 1 |
| outer | 34 | 14 |
| outer/lateral | 1 | 1 |
| Outerbridge | 3 | 3 |
| outermost | 1 | 1 |
| outlined | 2 | 2 |
| outlines | 1 | 1 |
| outpatient | 14 | 7 |
| outpatients | 1 | 1 |
| outside | 20 | 12 |
| outward | 2 | 2 |
| outwards | 1 | 1 |
| over | 81 | 27 |
| over-correct | 1 | 1 |
| over-corrected | 1 | 1 |
| over-correction | 3 | 3 |
| over-or | 1 | 1 |
| over-stress | 1 | 1 |
| overall | 11 | 8 |
| overcome | 3 | 3 |
| overcorrect | 1 | 1 |
| overcorrected | 4 | 2 |
| overcorrection | 23 | 10 |
| overlaid | 1 | 1 |
| overlap | 1 | 1 |
| overlapped | 1 | 1 |
| overlapping | 1 | 1 |
| overload | 8 | 5 |
| overloading | 3 | 2 |
| overlying | 1 | 1 |
| overnight | 1 | 1 |
| overseeing | 1 | 1 |
| overshoot | 1 | 1 |
| overstated | 1 | 1 |
| overview | 2 | 1 |
| overweight | 7 | 6 |
| overweighted | 1 | 1 |
| OW | 1 | 1 |
| OWHTO | 17 | 2 |
| owing | 2 | 1 |
| own | 13 | 9 |
| Oxford | 1 | 1 |
| oxycodone | 1 | 1 |
| oxygen | 2 | 1 |
| oxygenation | 1 | 1 |
| p | 11 | 3 |
| p-values | 1 | 1 |
| pa-tients | 3 | 1 |
| pack | 3 | 2 |
| package | 3 | 3 |
| packs | 4 | 3 |
| PACS | 2 | 2 |
| padded | 1 | 1 |
| paediatric | 1 | 1 |
| pain | 311 | 38 |
| pain-free | 4 | 4 |
| pain-relieving | 1 | 1 |
| pain/edema | 2 | 1 |
| painful | 20 | 13 |
| painkiller | 1 | 1 |
| painkillers | 2 | 1 |
| painless | 1 | 1 |
| painlessly | 1 | 1 |
| pair | 1 | 1 |
| paired | 1 | 1 |
| pale | 1 | 1 |
| paler | 1 | 1 |
| palliative | 2 | 2 |
| palsy | 4 | 2 |
| Pape | 1 | 1 |
| paper | 4 | 4 |
| papers | 8 | 4 |
| paperwork | 2 | 2 |
| paracetamol | 1 | 1 |
| parallel | 17 | 9 |
| parameter | 1 | 1 |
| parameters | 10 | 6 |
| pars | 2 | 1 |
| part | 51 | 25 |
| partial | 45 | 21 |
| partial-and | 1 | 1 |
| partially | 5 | 4 |
| participate | 4 | 4 |
| participated | 1 | 1 |
| participating | 1 | 1 |
| participation | 2 | 2 |
| particular | 10 | 7 |
| particularly | 15 | 8 |
| parts | 3 | 1 |
| PAS | 8 | 1 |
| pass | 16 | 6 |
| passed | 7 | 6 |
| passes | 13 | 7 |
| passing | 7 | 5 |
| passionately | 1 | 1 |
| passive | 15 | 8 |
| passively | 1 | 1 |
| past | 16 | 10 |
| patch | 2 | 2 |
| patella | 53 | 16 |
| patellar | 69 | 14 |
| patellectomy | 1 | 1 |
| patello-femoral | 2 | 2 |
| patellofemoral | 25 | 14 |
| pathologic | 2 | 2 |
| pathological | 5 | 4 |
| pathologically | 1 | 1 |
| pathologies | 12 | 5 |
| pathology | 3 | 3 |
| Patient | 264 | 31 |
| patient’s | 40 | 17 |
| patient-controlled | 1 | 1 |
| patient-reported | 2 | 2 |
| patient-specific | 6 | 2 |
| patient`s | 1 | 1 |
| patients | 641 | 42 |
| Patte | 1 | 1 |
| pattern | 9 | 6 |
| patterns | 3 | 3 |
| payment | 1 | 1 |
| PCA | 1 | 1 |
| PCL | 18 | 5 |
| PCR | 2 | 1 |
| peak | 5 | 2 |
| pear | 1 | 1 |
| Pearson | 2 | 1 |
| Pearson's | 2 | 1 |
| peas | 2 | 2 |
| pediatric | 2 | 1 |
| PEEK | 2 | 1 |
| PEEK-Power | 1 | 1 |
| PeekMed | 1 | 1 |
| PEEKPower | 3 | 2 |
| peel | 1 | 1 |
| pelvic | 4 | 4 |
| pelvis | 3 | 3 |
| pendulum | 1 | 1 |
| pendulums | 1 | 1 |
| penicillin | 1 | 1 |
| people | 36 | 10 |
| per | 7 | 4 |
| percent | 9 | 5 |
| percentage | 7 | 7 |
| perception | 1 | 1 |
| perfect | 2 | 1 |
| perfectly | 2 | 1 |
| perforated | 2 | 2 |
| perform | 34 | 16 |
| performance | 3 | 3 |
| performed | 169 | 34 |
| performing | 19 | 11 |
| perhaps | 5 | 1 |
| periarticular | 2 | 2 |
| period | 43 | 21 |
| periods | 4 | 3 |
| periosteal | 2 | 2 |
| periosteum | 5 | 4 |
| peripheral | 2 | 2 |
| permanent | 3 | 3 |
| permanently | 1 | 1 |
| permission | 5 | 2 |
| permit | 1 | 1 |
| permits | 5 | 3 |
| permitted | 3 | 2 |
| peroneal | 20 | 11 |
| perpendicular | 4 | 4 |
| persist | 1 | 1 |
| persistence | 1 | 1 |
| persistent | 4 | 3 |
| persisting | 1 | 1 |
| person | 14 | 4 |
| person’s | 2 | 2 |
| personal | 3 | 3 |
| personalized | 2 | 1 |
| PEs | 13 | 4 |
| Peter | 4 | 1 |
| Peto | 1 | 1 |
| PF | 1 | 1 |
| PH | 2 | 1 |
| pharmaceutical | 1 | 1 |
| pharmacies | 1 | 1 |
| pharmacist | 1 | 1 |
| pharmacy | 1 | 1 |
| Phase | 12 | 5 |
| phases | 1 | 1 |
| phosphate | 8 | 4 |
| photo | 7 | 1 |
| photograph | 1 | 1 |
| photos | 1 | 1 |
| Photoshop | 1 | 1 |
| physical | 43 | 20 |
| physically | 12 | 8 |
| physician | 7 | 2 |
| physicians | 6 | 1 |
| physiological | 1 | 1 |
| physiologically | 1 | 1 |
| physiotherapist | 27 | 9 |
| physiotherapists | 2 | 2 |
| Physiotherapy | 12 | 4 |
| pick | 2 | 1 |
| picked | 1 | 1 |
| picking | 1 | 1 |
| picture | 4 | 4 |
| pictures | 5 | 2 |
| piece | 3 | 3 |
| Pieces | 1 | 1 |
| pill | 2 | 1 |
| pillow | 1 | 1 |
| pillows | 2 | 2 |
| Pills | 2 | 1 |
| pilot | 1 | 1 |
| pimples | 1 | 1 |
| pin | 16 | 5 |
| pin-hole | 1 | 1 |
| pin-tract | 2 | 1 |
| pins | 19 | 6 |
| pioneer | 1 | 1 |
| piperacillin/tazobactam | 1 | 1 |
| piriformis | 1 | 1 |
| pivot | 1 | 1 |
| pivoting | 1 | 1 |
| place | 31 | 17 |
| placed | 24 | 16 |
| placement | 1 | 1 |
| places | 1 | 1 |
| placing | 4 | 2 |
| plain | 2 | 2 |
| plan | 15 | 12 |
| plane | 51 | 10 |
| planes | 8 | 6 |
| planned | 16 | 9 |
| planning | 39 | 13 |
| plans | 1 | 1 |
| plasma | 3 | 3 |
| plaster | 7 | 1 |
| plastic | 4 | 4 |
| plate | 207 | 31 |
| plateau | 55 | 12 |
| plateaus | 2 | 2 |
| platelet | 1 | 1 |
| platelet-rich | 1 | 1 |
| platelets | 1 | 1 |
| plates | 47 | 14 |
| platform | 2 | 1 |
| plating | 5 | 2 |
| plausibility | 1 | 1 |
| play | 4 | 4 |
| playing | 2 | 2 |
| plays | 1 | 1 |
| PLC | 3 | 1 |
| please | 11 | 6 |
| plenty | 1 | 1 |
| plus | 4 | 3 |
| PMMA | 3 | 1 |
| pneumatic | 2 | 1 |
| pneumonia | 1 | 1 |
| point | 36 | 15 |
| point5 | 1 | 1 |
| points | 20 | 4 |
| poison | 2 | 2 |
| policies | 1 | 1 |
| polished | 1 | 1 |
| Pollard | 1 | 1 |
| polyaxial | 1 | 1 |
| polymerase | 1 | 1 |
| polymethylmethacrylate | 1 | 1 |
| pool | 1 | 1 |
| pooling | 1 | 1 |
| poor | 9 | 6 |
| poorer | 1 | 1 |
| poorly-controlled | 1 | 1 |
| popliteal | 3 | 1 |
| popping | 3 | 2 |
| popular | 13 | 7 |
| popularised | 1 | 1 |
| popularity | 2 | 2 |
| popularized | 2 | 2 |
| population | 17 | 8 |
| population-based | 4 | 2 |
| portals | 1 | 1 |
| portion | 17 | 11 |
| portions | 3 | 1 |
| pose | 2 | 1 |
| poses | 1 | 1 |
| position | 59 | 19 |
| positioned | 5 | 5 |
| positioning | 11 | 4 |
| positive | 7 | 5 |
| possess | 2 | 2 |
| possibilities | 1 | 1 |
| possibility | 4 | 3 |
| possible | 44 | 21 |
| possibly | 2 | 2 |
| post | 14 | 8 |
| post-op | 2 | 2 |
| post-operative | 11 | 8 |
| post-operatively | 9 | 4 |
| post-surgery | 2 | 2 |
| post-surgical | 7 | 2 |
| post-traumatic | 2 | 1 |
| postcorrection | 1 | 1 |
| posterior | 100 | 14 |
| posterior-posterolateral | 1 | 1 |
| Posterior-stabilized | 1 | 1 |
| posteriorlateral | 1 | 1 |
| posteriorly | 7 | 5 |
| postero-anterior | 1 | 1 |
| postero-medial | 1 | 1 |
| posteroanterior | 4 | 4 |
| posterolateral | 17 | 10 |
| posteromedial | 13 | 5 |
| postop | 1 | 1 |
| postopera-tively | 1 | 1 |
| postoperation | 2 | 1 |
| postoperative | 67 | 14 |
| postoperatively | 25 | 7 |
| postpone | 3 | 2 |
| postponement | 1 | 1 |
| postpones | 1 | 1 |
| Postponing | 2 | 2 |
| posttraumatic | 6 | 2 |
| potential | 21 | 14 |
| potentially | 5 | 5 |
| pottering | 1 | 1 |
| pounding | 1 | 1 |
| power | 1 | 1 |
| powerful | 1 | 1 |
| Powerplate | 1 | 1 |
| practice | 8 | 7 |
| practices | 1 | 1 |
| practicing | 1 | 1 |
| Practitioner | 1 | 1 |
| Prakash | 1 | 1 |
| pre | 1 | 1 |
| pre-admission | 1 | 1 |
| pre-assessment | 1 | 1 |
| pre-existing | 1 | 1 |
| pre-made | 1 | 1 |
| pre-operative | 13 | 6 |
| pre-operatively | 1 | 1 |
| Precautions | 3 | 2 |
| preceding | 2 | 2 |
| prechronic | 1 | 1 |
| precise | 14 | 7 |
| precisely | 3 | 3 |
| preciseness | 1 | 1 |
| precision | 3 | 3 |
| precontoured | 1 | 1 |
| predecessor | 1 | 1 |
| predecessors | 1 | 1 |
| predetermined | 1 | 1 |
| predict | 3 | 2 |
| predictable | 3 | 3 |
| predicted | 1 | 1 |
| predictive | 2 | 2 |
| predictor | 4 | 2 |
| predictors | 1 | 1 |
| predominant | 2 | 2 |
| predominantly | 5 | 4 |
| preexisting | 3 | 2 |
| prefer | 3 | 3 |
| preferable | 1 | 1 |
| preferably | 3 | 2 |
| preference | 1 | 1 |
| preferences | 1 | 1 |
| preferred | 10 | 6 |
| preformed | 1 | 1 |
| pregabalin | 1 | 1 |
| prehab | 1 | 1 |
| preliminary | 1 | 1 |
| premature | 1 | 1 |
| preopera-tively | 1 | 1 |
| preoperational | 1 | 1 |
| preoperative | 40 | 13 |
| preoperatively | 14 | 6 |
| preparation | 6 | 5 |
| preparations | 11 | 6 |
| prepared | 5 | 4 |
| prepares | 1 | 1 |
| Preparing | 2 | 2 |
| prerequisites | 1 | 1 |
| prescribe | 4 | 4 |
| prescribed | 16 | 6 |
| prescription | 1 | 1 |
| prescription-strength | 1 | 1 |
| presence | 11 | 6 |
| present | 18 | 13 |
| presentations | 1 | 1 |
| presented | 8 | 5 |
| presenting | 3 | 3 |
| Presently | 1 | 1 |
| presents | 4 | 2 |
| preservation | 11 | 7 |
| preserve | 5 | 5 |
| preserved | 5 | 5 |
| preserves | 5 | 4 |
| preserving | 5 | 5 |
| press-fit | 1 | 1 |
| pressed | 1 | 1 |
| pressing | 3 | 3 |
| pressure | 55 | 20 |
| pressure-induced | 1 | 1 |
| pressure/weight | 1 | 1 |
| pressures | 3 | 1 |
| pretensioned | 1 | 1 |
| pretty | 3 | 1 |
| prevailing | 1 | 1 |
| prevalence | 2 | 2 |
| prevent | 33 | 18 |
| prevented | 1 | 1 |
| preventing | 4 | 3 |
| prevention | 2 | 2 |
| prevents | 3 | 2 |
| previous | 23 | 14 |
| Previously | 3 | 2 |
| pried | 1 | 1 |
| primarily | 2 | 2 |
| primary | 28 | 13 |
| principal | 2 | 2 |
| principle | 3 | 3 |
| principles | 4 | 3 |
| printed | 6 | 2 |
| printer | 1 | 1 |
| printing | 2 | 2 |
| prior | 28 | 15 |
| priorities | 1 | 1 |
| priority | 1 | 1 |
| private | 1 | 1 |
| privately | 1 | 1 |
| PRN | 1 | 1 |
| proactive | 1 | 1 |
| probability | 2 | 2 |
| probable | 1 | 1 |
| probably | 8 | 5 |
| problem | 30 | 6 |
| problematic | 3 | 3 |
| problems | 38 | 15 |
| procedural | 1 | 1 |
| procedure | 208 | 40 |
| Procedure-specific | 1 | 1 |
| procedurees | 1 | 1 |
| procedures | 79 | 20 |
| proceed | 2 | 2 |
| process | 23 | 15 |
| processes | 3 | 2 |
| processing | 1 | 1 |
| procrastinating | 1 | 1 |
| procured | 1 | 1 |
| Prodromos | 1 | 1 |
| produce | 5 | 4 |
| produced | 4 | 4 |
| produces | 5 | 3 |
| producing | 1 | 1 |
| product | 3 | 2 |
| professional | 4 | 3 |
| Professor | 3 | 1 |
| proficient | 1 | 1 |
| profile | 10 | 4 |
| profound | 1 | 1 |
| prognosis | 2 | 2 |
| prognostic | 11 | 3 |
| program | 7 | 4 |
| programme | 3 | 1 |
| programs | 1 | 1 |
| progress | 17 | 6 |
| progresses | 4 | 4 |
| progressing | 4 | 4 |
| progression | 37 | 14 |
| Progressive | 18 | 10 |
| progressively | 6 | 4 |
| prohibit | 1 | 1 |
| projection | 1 | 1 |
| projections | 1 | 1 |
| proliferation | 1 | 1 |
| prolong | 5 | 4 |
| prolonged | 3 | 2 |
| prolonging | 2 | 2 |
| prolongs | 4 | 3 |
| prominence | 2 | 1 |
| prominent | 1 | 1 |
| promising | 1 | 1 |
| promote | 9 | 7 |
| promotes | 1 | 1 |
| promoting | 1 | 1 |
| Prone | 6 | 4 |
| pronounced | 2 | 2 |
| propagated | 1 | 1 |
| propagation | 1 | 1 |
| propensity | 2 | 1 |
| proper | 10 | 9 |
| properly | 13 | 11 |
| properties | 3 | 3 |
| prophylactic | 1 | 1 |
| proportion | 3 | 2 |
| propose | 1 | 1 |
| proposed | 9 | 4 |
| prospective | 8 | 4 |
| prospectively | 1 | 1 |
| prosthesis | 14 | 4 |
| prosthetic | 3 | 3 |
| protect | 16 | 13 |
| protected | 8 | 6 |
| protecting | 4 | 3 |
| protection | 3 | 3 |
| protective | 3 | 3 |
| protects | 3 | 3 |
| protein | 1 | 1 |
| protocol | 18 | 7 |
| protocols | 1 | 1 |
| protrusion | 1 | 1 |
| proved | 1 | 1 |
| proven | 8 | 7 |
| proves | 1 | 1 |
| provide | 27 | 17 |
| provided | 9 | 7 |
| providers | 1 | 1 |
| provides | 15 | 13 |
| providing | 1 | 1 |
| provocative | 1 | 1 |
| proximal | 99 | 17 |
| proximally | 5 | 5 |
| proximity | 1 | 1 |
| prudent | 1 | 1 |
| prunes | 2 | 2 |
| pseudarthrosis | 3 | 2 |
| pseudoarthrosis | 2 | 2 |
| pseudolaxity | 1 | 1 |
| PSI | 6 | 1 |
| psoriatic | 1 | 1 |
| PT | 5 | 1 |
| pts | 9 | 3 |
| PTSA | 1 | 1 |
| public | 1 | 1 |
| publications | 1 | 1 |
| published | 11 | 9 |
| PubMed | 2 | 1 |
| Puddu | 13 | 7 |
| Puddu’s | 1 | 1 |
| puffy | 2 | 2 |
| Pull | 7 | 3 |
| pulled | 2 | 1 |
| pulleys/bands | 1 | 1 |
| pullout | 1 | 1 |
| pulmonary | 4 | 3 |
| pump | 2 | 2 |
| pumping | 1 | 1 |
| puncture | 2 | 1 |
| purchased | 1 | 1 |
| pure | 2 | 2 |
| purely | 1 | 1 |
| purpose | 5 | 4 |
| purposes | 3 | 3 |
| pursuits | 1 | 1 |
| purulent | 1 | 1 |
| pus | 1 | 1 |
| push | 2 | 2 |
| pushed | 1 | 1 |
| pushes | 1 | 1 |
| pushing | 1 | 1 |
| put | 39 | 11 |
| puts | 1 | 1 |
| putting | 13 | 8 |
| pVAS | 4 | 1 |
| quadratic | 1 | 1 |
| quadriceps | 14 | 7 |
| Quadruped | 1 | 1 |
| quads | 2 | 2 |
| qualify | 1 | 1 |
| qualitative | 1 | 1 |
| quality | 6 | 5 |
| quantifying | 1 | 1 |
| quantitative | 2 | 2 |
| question | 2 | 2 |
| questionnaire | 3 | 2 |
| questions | 3 | 3 |
| quick | 1 | 1 |
| quickly | 3 | 3 |
| quintupled | 1 | 1 |
| quit | 1 | 1 |
| quite | 27 | 7 |
| quitting | 1 | 1 |
| quote | 1 | 1 |
| quoted | 1 | 1 |
| r | 1 | 1 |
| RA | 19 | 1 |
| Raaij | 1 | 1 |
| racquet | 1 | 1 |
| radial | 1 | 1 |
| radiation | 1 | 1 |
| radically | 1 | 1 |
| radiograph | 7 | 4 |
| radiographic | 14 | 8 |
| radiographically | 1 | 1 |
| radiographs | 25 | 12 |
| radiography | 8 | 6 |
| radiologic | 1 | 1 |
| radiological | 4 | 4 |
| radiologically | 1 | 1 |
| radiology | 3 | 3 |
| radiolucent | 2 | 2 |
| railings | 1 | 1 |
| rails | 1 | 1 |
| raise | 3 | 3 |
| raises | 2 | 1 |
| raising | 1 | 1 |
| randomised | 1 | 1 |
| randomized | 1 | 1 |
| range | 77 | 27 |
| range-of-motion | 1 | 1 |
| ranged | 1 | 1 |
| ranges | 2 | 2 |
| ranging | 2 | 2 |
| rapid | 6 | 6 |
| rapidly | 1 | 1 |
| rare | 10 | 4 |
| rarely | 8 | 6 |
| rarer | 2 | 2 |
| rate | 59 | 20 |
| rated | 1 | 1 |
| rates | 26 | 14 |
| rather | 15 | 9 |
| rating | 4 | 1 |
| ratio | 10 | 5 |
| rationale | 2 | 2 |
| rays | 2 | 1 |
| re | 1 | 1 |
| re-aligned | 1 | 1 |
| re-aligning | 2 | 1 |
| re-arranging | 1 | 1 |
| re-discovered | 1 | 1 |
| re-do | 2 | 1 |
| Re-evaluation | 1 | 1 |
| re-examined | 1 | 1 |
| re-implantation | 1 | 1 |
| re-introduced | 1 | 1 |
| re-osteotomies | 1 | 1 |
| re-osteotomy | 2 | 2 |
| re-sponded | 1 | 1 |
| re-tained | 1 | 1 |
| re-tear | 1 | 1 |
| re-tension | 1 | 1 |
| re-wrap | 1 | 1 |
| reach | 5 | 5 |
| reached | 2 | 2 |
| reaches | 1 | 1 |
| reaching | 2 | 2 |
| reaction | 4 | 4 |
| reading | 1 | 1 |
| readjust | 1 | 1 |
| Readjust-ment | 1 | 1 |
| readjusted | 2 | 2 |
| Ready | 4 | 3 |
| real | 5 | 2 |
| real-time | 1 | 1 |
| realign | 22 | 11 |
| realigned | 5 | 3 |
| realigning | 5 | 3 |
| realignment | 30 | 12 |
| realignments | 1 | 1 |
| realigns | 3 | 3 |
| realised | 1 | 1 |
| realistic | 2 | 2 |
| reality | 1 | 1 |
| really | 21 | 1 |
| reapply | 1 | 1 |
| reappreciated | 1 | 1 |
| reappreciation | 1 | 1 |
| rearranged | 1 | 1 |
| rearrangement | 1 | 1 |
| reason | 10 | 7 |
| reasonable | 6 | 5 |
| reasonably | 3 | 2 |
| reasons | 10 | 8 |
| RebalanceMD | 7 | 1 |
| receive | 3 | 2 |
| received | 13 | 4 |
| receives | 1 | 1 |
| receiving | 6 | 4 |
| Recent | 25 | 12 |
| recently | 22 | 9 |
| reciprocating | 1 | 1 |
| recision | 1 | 1 |
| recognized | 2 | 2 |
| recom-mendations | 1 | 1 |
| recommend | 12 | 5 |
| recommendation | 1 | 1 |
| Recommendations | 6 | 5 |
| recommended | 28 | 13 |
| recommends | 1 | 1 |
| reconfigure | 2 | 2 |
| reconstruct | 1 | 1 |
| reconstructed | 2 | 2 |
| reconstructing | 1 | 1 |
| reconstruction | 67 | 11 |
| reconstructions | 3 | 3 |
| reconstructive | 2 | 1 |
| record | 2 | 1 |
| recorded | 4 | 3 |
| records | 6 | 2 |
| recover | 10 | 8 |
| recovered | 2 | 2 |
| recovering | 4 | 4 |
| recovery | 49 | 20 |
| recreational | 7 | 5 |
| rectangular | 1 | 1 |
| rectus | 1 | 1 |
| recurred | 1 | 1 |
| recurrence | 5 | 4 |
| recurrent | 2 | 2 |
| recurring | 1 | 1 |
| recurvatum | 2 | 1 |
| red | 4 | 4 |
| red-red | 1 | 1 |
| redistribute | 1 | 1 |
| redistributes | 1 | 1 |
| redistributing | 1 | 1 |
| redness | 4 | 3 |
| reduce | 61 | 23 |
| reduced | 14 | 9 |
| reduces | 12 | 9 |
| reducing | 17 | 12 |
| reduction | 9 | 5 |
| redundancy | 1 | 1 |
| refer | 1 | 1 |
| reference | 1 | 1 |
| referral | 1 | 1 |
| referrals | 1 | 1 |
| referred | 1 | 1 |
| referring | 1 | 1 |
| refers | 1 | 1 |
| refined | 2 | 2 |
| reflect | 3 | 3 |
| reflected | 1 | 1 |
| Reflecting | 1 | 1 |
| reflects | 1 | 1 |
| reflex | 1 | 1 |
| reflexive | 1 | 1 |
| refrozen | 1 | 1 |
| refus-ing | 1 | 1 |
| refused | 1 | 1 |
| regain | 3 | 3 |
| regained | 1 | 1 |
| Regaining | 2 | 2 |
| regard | 5 | 5 |
| regarded | 1 | 1 |
| regarding | 17 | 7 |
| Regardless | 4 | 3 |
| regards | 7 | 6 |
| regenerate | 2 | 2 |
| regenerated | 2 | 2 |
| regeneration | 10 | 4 |
| regenerative | 4 | 2 |
| regime | 1 | 1 |
| regimen | 3 | 3 |
| regimens | 2 | 1 |
| region | 9 | 2 |
| regional | 4 | 3 |
| regions | 2 | 2 |
| register | 6 | 1 |
| registered | 3 | 1 |
| registering | 1 | 1 |
| Registers | 1 | 1 |
| registrar | 1 | 1 |
| registration | 2 | 1 |
| registrations | 1 | 1 |
| Registry | 1 | 1 |
| registry-based | 1 | 1 |
| regression | 4 | 3 |
| regular | 11 | 10 |
| regularly | 6 | 4 |
| regulate | 1 | 1 |
| rehab | 1 | 1 |
| Rehabilitation | 36 | 16 |
| rehabilitative | 1 | 1 |
| reimburse | 1 | 1 |
| reimplantation | 1 | 1 |
| reinforce | 1 | 1 |
| reinterventions | 1 | 1 |
| reintroduced | 1 | 1 |
| related | 29 | 10 |
| relation | 1 | 1 |
| relationship | 8 | 5 |
| relationships | 2 | 2 |
| relative | 9 | 6 |
| relatively | 29 | 7 |
| relax | 3 | 2 |
| relaxant | 1 | 1 |
| relaxation | 1 | 1 |
| release | 5 | 5 |
| released | 4 | 3 |
| relevant | 4 | 3 |
| reliable | 8 | 3 |
| relief | 35 | 19 |
| relies | 2 | 2 |
| relieve | 28 | 15 |
| relieved | 1 | 1 |
| relieves | 2 | 2 |
| relieving | 7 | 6 |
| rely | 1 | 1 |
| remain | 13 | 11 |
| remained | 4 | 1 |
| remaining | 8 | 8 |
| remains | 14 | 8 |
| Remember | 5 | 3 |
| remission | 2 | 1 |
| remnant | 2 | 2 |
| remodeled | 2 | 2 |
| remodeling | 1 | 1 |
| remodelling | 1 | 1 |
| removal | 28 | 11 |
| remove | 30 | 18 |
| removed | 56 | 26 |
| removes | 8 | 6 |
| removing | 11 | 7 |
| rendered | 1 | 1 |
| renders | 1 | 1 |
| reoperation | 1 | 1 |
| reosteosynthesis | 1 | 1 |
| repair | 28 | 12 |
| repaired | 1 | 1 |
| reparative | 2 | 2 |
| Repeat | 10 | 3 |
| repeated | 3 | 3 |
| repetitive | 3 | 3 |
| replaced | 5 | 4 |
| replacement | 140 | 30 |
| replacements | 13 | 5 |
| replaces | 2 | 1 |
| replacing | 1 | 1 |
| report | 16 | 11 |
| reported | 62 | 12 |
| reporting | 1 | 1 |
| Reports | 9 | 7 |
| repositioned | 2 | 2 |
| repositioning | 1 | 1 |
| represent | 2 | 2 |
| represented | 1 | 1 |
| represents | 1 | 1 |
| reproduced | 4 | 1 |
| reproducibility | 2 | 2 |
| reproducible | 1 | 1 |
| reputation | 1 | 1 |
| request | 3 | 3 |
| require | 30 | 17 |
| required | 51 | 24 |
| requirement | 2 | 2 |
| requirements | 1 | 1 |
| requires | 7 | 4 |
| requiring | 9 | 8 |
| rescheduled | 1 | 1 |
| research | 13 | 7 |
| Researchers | 2 | 2 |
| resected | 5 | 3 |
| resection | 5 | 3 |
| resectomy | 3 | 1 |
| reserve | 1 | 1 |
| reserved | 4 | 4 |
| reshape | 2 | 1 |
| reshaped | 3 | 3 |
| reshaping | 3 | 1 |
| residual | 4 | 4 |
| resin | 1 | 1 |
| resist | 2 | 2 |
| resistance | 8 | 5 |
| resolution | 1 | 1 |
| resolve | 2 | 2 |
| resolved | 1 | 1 |
| resonance | 4 | 4 |
| resorbable | 1 | 1 |
| resources | 1 | 1 |
| respectively | 11 | 8 |
| respiratory | 1 | 1 |
| respond | 3 | 3 |
| responds | 1 | 1 |
| response | 3 | 2 |
| Rest | 11 | 7 |
| restart | 1 | 1 |
| resting | 4 | 4 |
| restoration | 5 | 5 |
| restore | 14 | 10 |
| restored | 2 | 1 |
| restores | 1 | 1 |
| restoring | 5 | 4 |
| restricted | 3 | 3 |
| restriction | 3 | 2 |
| restrictions | 9 | 7 |
| restricts | 1 | 1 |
| result | 64 | 25 |
| resultant | 4 | 3 |
| resulted | 5 | 4 |
| resulting | 14 | 13 |
| results | 129 | 23 |
| resume | 4 | 4 |
| resumed | 2 | 2 |
| resuming | 1 | 1 |
| resurfacing | 4 | 3 |
| resurgence | 2 | 1 |
| retain | 1 | 1 |
| retained | 1 | 1 |
| retention | 1 | 1 |
| retracted | 4 | 2 |
| retracting | 1 | 1 |
| retraction | 2 | 2 |
| retractor | 10 | 5 |
| retractors | 2 | 2 |
| retraining | 1 | 1 |
| retrospective | 6 | 4 |
| retrospectively | 1 | 1 |
| return | 44 | 15 |
| returned | 7 | 3 |
| Returning | 2 | 2 |
| returns | 1 | 1 |
| reveal | 1 | 1 |
| revealed | 5 | 4 |
| reveals | 1 | 1 |
| reverse | 7 | 2 |
| reversed | 1 | 1 |
| reversion | 1 | 1 |
| review | 17 | 11 |
| reviewed | 5 | 4 |
| reviewing | 1 | 1 |
| revise | 1 | 1 |
| revised | 1 | 1 |
| revision | 30 | 9 |
| revisions | 2 | 1 |
| revolutionary | 6 | 1 |
| revolutionised | 2 | 1 |
| rheumatoid | 11 | 10 |
| rheumatologist | 3 | 1 |
| Rheumatology | 2 | 1 |
| rheumosurgery | 1 | 1 |
| RHK | 2 | 1 |
| rich | 1 | 1 |
| rid | 2 | 1 |
| ride | 1 | 1 |
| rides | 3 | 2 |
| right | 35 | 8 |
| right-angle | 1 | 1 |
| rightly | 1 | 1 |
| rigid | 12 | 6 |
| rigidity | 3 | 2 |
| rigidly | 1 | 1 |
| Ring | 2 | 2 |
| rings | 1 | 1 |
| Rinonapoli | 1 | 1 |
| rise | 1 | 1 |
| rising | 2 | 2 |
| risk | 87 | 27 |
| risks | 25 | 14 |
| RNW | 1 | 1 |
| robotic | 1 | 1 |
| robust | 2 | 1 |
| rock | 1 | 1 |
| rod | 2 | 1 |
| Rodriguez-Merchan | 1 | 1 |
| roengeur | 1 | 1 |
| role | 4 | 3 |
| roll | 3 | 2 |
| ROM | 26 | 5 |
| ROM/Mobility | 1 | 1 |
| room | 20 | 10 |
| root | 4 | 1 |
| Rosenberg | 7 | 5 |
| Rosenberg/weightbearing | 1 | 1 |
| rotated | 1 | 1 |
| rotating | 3 | 1 |
| rotation | 13 | 7 |
| rotational | 5 | 4 |
| rough | 2 | 2 |
| roughly | 1 | 1 |
| rounded | 1 | 1 |
| routine | 6 | 4 |
| routinely | 10 | 5 |
| RR | 5 | 1 |
| rub | 1 | 1 |
| rubbing | 1 | 1 |
| rugs | 2 | 2 |
| rule | 1 | 1 |
| ruler | 1 | 1 |
| rules | 1 | 1 |
| run | 6 | 4 |
| running | 11 | 8 |
| runs | 2 | 2 |
| rupture | 1 | 1 |
| ruptured | 3 | 1 |
| Russia | 3 | 1 |
| RW | 1 | 1 |
| s | 4 | 3 |
| Saarakkala | 1 | 1 |
| safe | 29 | 18 |
| safely | 9 | 4 |
| safety | 7 | 5 |
| sagital | 1 | 1 |
| sagittal | 31 | 9 |
| said | 4 | 2 |
| Saint-Petersburg | 1 | 1 |
| sake | 1 | 1 |
| salvage | 3 | 2 |
| same | 37 | 19 |
| sample | 2 | 2 |
| sample-size | 1 | 1 |
| samples | 1 | 1 |
| sampling | 1 | 1 |
| sandwich | 2 | 1 |
| Sartorial | 1 | 1 |
| Sartorius | 2 | 2 |
| satisfac-tion | 1 | 1 |
| satisfaction | 7 | 4 |
| satisfactory | 17 | 5 |
| satisfied | 10 | 5 |
| satisfying | 3 | 3 |
| save | 2 | 1 |
| saw | 22 | 15 |
| sawblade | 3 | 1 |
| Sawbone | 2 | 1 |
| say | 13 | 1 |
| says | 2 | 1 |
| Scale | 10 | 5 |
| Scale/ | 1 | 1 |
| scales | 3 | 2 |
| scan | 9 | 7 |
| Scanning | 1 | 1 |
| scanogram | 2 | 1 |
| scanograms | 3 | 1 |
| scans | 4 | 3 |
| scar | 8 | 4 |
| scarce | 1 | 1 |
| scenario | 6 | 4 |
| scenarios | 1 | 1 |
| Schallberger | 3 | 3 |
| schedule | 2 | 2 |
| scheduled | 1 | 1 |
| Schematic | 1 | 1 |
| science | 1 | 1 |
| Sciences | 1 | 1 |
| scientific | 3 | 2 |
| scientists | 1 | 1 |
| scintigraphy | 1 | 1 |
| sclerosis | 2 | 2 |
| scope | 1 | 1 |
| score | 33 | 8 |
| scores | 12 | 6 |
| scoring | 1 | 1 |
| scrape | 1 | 1 |
| scrapes | 2 | 2 |
| screen | 1 | 1 |
| screening | 1 | 1 |
| screw | 40 | 10 |
| screw-hole | 1 | 1 |
| screwed | 1 | 1 |
| screws | 69 | 28 |
| scrutinized | 1 | 1 |
| SD | 1 | 1 |
| search | 2 | 1 |
| seat | 6 | 2 |
| seated | 4 | 1 |
| second | 26 | 14 |
| second-generation | 1 | 1 |
| second-look | 5 | 1 |
| secondary | 24 | 13 |
| Secondly | 2 | 2 |
| seconds | 5 | 3 |
| section | 2 | 2 |
| sector | 1 | 1 |
| secure | 2 | 2 |
| secured | 5 | 4 |
| securely | 1 | 1 |
| securing | 1 | 1 |
| sedate | 1 | 1 |
| sedated | 1 | 1 |
| sedation | 1 | 1 |
| sedentary | 4 | 2 |
| see | 36 | 12 |
| seeing | 2 | 1 |
| seek | 2 | 2 |
| seeking | 1 | 1 |
| seem | 4 | 4 |
| seems | 5 | 5 |
| seen | 18 | 9 |
| segment | 8 | 4 |
| segmentation | 1 | 1 |
| segments | 1 | 1 |
| Seki | 1 | 1 |
| select | 6 | 5 |
| selected | 4 | 4 |
| Selection | 34 | 15 |
| selection.1 | 1 | 1 |
| selective | 3 | 1 |
| selects | 1 | 1 |
| sells | 1 | 1 |
| semiquantitative | 1 | 1 |
| senior | 2 | 2 |
| sensation | 4 | 3 |
| sense | 1 | 1 |
| sensible | 1 | 1 |
| sensitive | 2 | 2 |
| Sensory | 3 | 3 |
| sensory/motor | 1 | 1 |
| sent | 2 | 2 |
| separate | 5 | 4 |
| separated | 3 | 3 |
| separately | 1 | 1 |
| separating | 1 | 1 |
| separation | 1 | 1 |
| septic | 7 | 1 |
| sequence | 1 | 1 |
| sequential | 1 | 1 |
| serial | 1 | 1 |
| series | 16 | 5 |
| Serious | 11 | 5 |
| seriously | 2 | 1 |
| serve | 1 | 1 |
| served | 1 | 1 |
| serves | 3 | 3 |
| services | 2 | 1 |
| session | 1 | 1 |
| set | 9 | 5 |
| sets | 1 | 1 |
| setting | 7 | 4 |
| settings | 1 | 1 |
| settled | 1 | 1 |
| seven | 4 | 4 |
| seventy-three | 1 | 1 |
| several | 35 | 18 |
| severe | 36 | 17 |
| severely | 4 | 2 |
| severence | 1 | 1 |
| severity | 17 | 12 |
| sex | 11 | 5 |
| sexes | 1 | 1 |
| SFLA | 1 | 1 |
| shades | 1 | 1 |
| shaft | 5 | 4 |
| shape | 6 | 5 |
| shaped | 7 | 4 |
| shapes | 1 | 1 |
| Shapiro-Wilk | 1 | 1 |
| share | 1 | 1 |
| shared | 1 | 1 |
| Sharma | 1 | 1 |
| sharply | 1 | 1 |
| shaved | 1 | 1 |
| she | 17 | 8 |
| shear | 4 | 3 |
| sheep | 2 | 1 |
| shift | 29 | 19 |
| shift/sway | 1 | 1 |
| shifted | 11 | 7 |
| shifting | 5 | 5 |
| shifts | 2 | 2 |
| shin | 31 | 14 |
| shinbone | 25 | 10 |
| shock | 6 | 2 |
| shock-absorber | 1 | 1 |
| shoes | 1 | 1 |
| shopping | 2 | 1 |
| short | 22 | 13 |
| short-term | 9 | 4 |
| shortcomings | 3 | 1 |
| shortened | 2 | 2 |
| shortening | 13 | 7 |
| shorter | 4 | 4 |
| shorts | 1 | 1 |
| should | 187 | 28 |
| shoulders | 1 | 1 |
| shouldn’t | 3 | 1 |
| show | 20 | 10 |
| showed | 49 | 13 |
| shower | 10 | 4 |
| showering | 3 | 2 |
| showers | 1 | 1 |
| showing | 3 | 3 |
| shown | 29 | 15 |
| shows | 15 | 8 |
| shut | 1 | 1 |
| Shuttle | 1 | 1 |
| sick | 2 | 2 |
| side | 140 | 32 |
| side-on | 2 | 1 |
| side-to-side | 1 | 1 |
| side.3 | 1 | 1 |
| side/lateral | 1 | 1 |
| sides | 5 | 5 |
| sign | 4 | 4 |
| signal | 1 | 1 |
| signed-rank | 1 | 1 |
| significance | 3 | 3 |
| significant | 69 | 18 |
| significantly | 26 | 15 |
| signs | 8 | 5 |
| silly | 1 | 1 |
| similar | 17 | 12 |
| similarly | 3 | 3 |
| simple | 15 | 10 |
| simpler | 1 | 1 |
| simplest | 1 | 1 |
| simply | 3 | 3 |
| simulated | 1 | 1 |
| simulation | 2 | 2 |
| simultaneous | 13 | 4 |
| simultaneously | 1 | 1 |
| sin-gle | 1 | 1 |
| Since | 20 | 12 |
| Single | 20 | 11 |
| single-leg | 2 | 2 |
| single-limb | 1 | 1 |
| Single-stage | 1 | 1 |
| single-staged | 1 | 1 |
| Sit | 4 | 3 |
| site | 73 | 21 |
| sits | 4 | 1 |
| sitting | 7 | 4 |
| situated | 1 | 1 |
| situation | 16 | 5 |
| situations | 1 | 1 |
| six | 41 | 13 |
| Sixteen | 1 | 1 |
| Sixty-six | 1 | 1 |
| size | 34 | 16 |
| sizes | 2 | 2 |
| SKAR | 10 | 1 |
| skid | 1 | 1 |
| skiing | 2 | 2 |
| skills | 4 | 2 |
| skin | 29 | 17 |
| skinny | 1 | 1 |
| skyline | 5 | 5 |
| sleep | 7 | 6 |
| sleeping | 3 | 3 |
| Sleeve | 2 | 1 |
| slice | 1 | 1 |
| slices | 1 | 1 |
| Slick | 2 | 2 |
| slide | 4 | 2 |
| slider | 2 | 1 |
| slides | 3 | 1 |
| Sliding | 3 | 2 |
| slight | 12 | 7 |
| slightly | 41 | 8 |
| Slippage | 1 | 1 |
| slippery | 1 | 1 |
| slips | 1 | 1 |
| slope | 111 | 11 |
| slope-decreasing | 1 | 1 |
| slope-increasing | 1 | 1 |
| slope-reducing | 1 | 1 |
| slot | 1 | 1 |
| slow | 9 | 8 |
| slower | 1 | 1 |
| slowing | 6 | 4 |
| slowly | 10 | 10 |
| slows | 3 | 3 |
| SLR | 2 | 1 |
| small | 41 | 20 |
| small-sized | 1 | 1 |
| small-type | 1 | 1 |
| smaller | 9 | 8 |
| SMCL | 10 | 1 |
| SMCL-transection | 1 | 1 |
| Smith | 1 | 1 |
| smoke | 2 | 2 |
| smoker | 1 | 1 |
| smokers | 7 | 3 |
| smoking | 25 | 10 |
| smooth | 6 | 6 |
| smoothly | 6 | 5 |
| snip | 1 | 1 |
| snug | 2 | 1 |
| snuggly | 1 | 1 |
| so | 137 | 22 |
| soak | 1 | 1 |
| soaking | 1 | 1 |
| soap | 1 | 1 |
| social | 2 | 2 |
| Society | 12 | 7 |
| sock | 1 | 1 |
| socket | 1 | 1 |
| socks | 2 | 2 |
| soft | 21 | 12 |
| soft-tissue | 4 | 1 |
| softener | 1 | 1 |
| software | 12 | 5 |
| soiled | 1 | 1 |
| sold | 1 | 1 |
| soldiered | 2 | 1 |
| sole | 2 | 1 |
| solely | 2 | 2 |
| soleus | 2 | 1 |
| solicited | 1 | 1 |
| solid | 2 | 2 |
| Solomin | 1 | 1 |
| solution | 5 | 5 |
| solutions | 2 | 1 |
| solve | 1 | 1 |
| solved | 1 | 1 |
| solving | 1 | 1 |
| somatic | 1 | 1 |
| Some | 122 | 32 |
| somebody | 2 | 1 |
| someone | 18 | 3 |
| something | 17 | 1 |
| sometime | 1 | 1 |
| sometimes | 19 | 7 |
| somewhat | 2 | 2 |
| somewhere | 2 | 2 |
| Song | 3 | 3 |
| sonication | 2 | 1 |
| soon | 11 | 9 |
| sooner | 3 | 2 |
| sophisticated | 3 | 1 |
| sophistication | 1 | 1 |
| sore | 1 | 1 |
| sort | 4 | 1 |
| sorted | 1 | 1 |
| sorts | 1 | 1 |
| sound | 1 | 1 |
| sounds | 1 | 1 |
| source | 1 | 1 |
| South | 1 | 1 |
| space | 43 | 22 |
| Spacer | 15 | 4 |
| spacers | 4 | 3 |
| spacing | 2 | 1 |
| Spahn | 1 | 1 |
| span | 5 | 4 |
| spared | 1 | 1 |
| speak | 2 | 2 |
| speaking | 3 | 2 |
| Spearman | 2 | 1 |
| special | 7 | 5 |
| specialise | 1 | 1 |
| specialised | 2 | 2 |
| specialist | 5 | 3 |
| specialists | 2 | 2 |
| specialized | 4 | 4 |
| specially | 3 | 1 |
| specific | 19 | 13 |
| specifically | 5 | 4 |
| specifics | 1 | 1 |
| specimens | 2 | 1 |
| Specogna | 1 | 1 |
| spectrum | 1 | 1 |
| speculate | 1 | 1 |
| speed | 3 | 3 |
| speeds | 1 | 1 |
| spend | 10 | 5 |
| spinal | 17 | 6 |
| spine | 9 | 7 |
| spines | 1 | 1 |
| spite | 5 | 5 |
| splint | 3 | 3 |
| spongiosa | 1 | 1 |
| sport | 22 | 6 |
| sporting | 5 | 3 |
| sports | 25 | 13 |
| sporty | 2 | 1 |
| spouse | 1 | 1 |
| spread | 3 | 2 |
| spreader | 4 | 4 |
| spreaders | 6 | 1 |
| spreading | 5 | 3 |
| Spring | 3 | 1 |
| sprinting | 1 | 1 |
| SPSS | 2 | 2 |
| squat | 2 | 2 |
| squats | 1 | 1 |
| squats/calf | 1 | 1 |
| squatting | 1 | 1 |
| Squeeze | 1 | 1 |
| squeezes | 1 | 1 |
| St | 1 | 1 |
| sta-bility | 1 | 1 |
| stabilisation | 1 | 1 |
| stabilise | 3 | 1 |
| stabilised | 1 | 1 |
| stabilising | 3 | 2 |
| stability | 45 | 15 |
| stabilization | 3 | 3 |
| stabilize | 6 | 5 |
| stabilized | 3 | 2 |
| stabilizers | 1 | 1 |
| stabilizing | 2 | 2 |
| stable | 18 | 11 |
| stacked | 2 | 2 |
| staff | 3 | 2 |
| stage | 33 | 8 |
| staged | 7 | 2 |
| stages | 12 | 6 |
| stained | 1 | 1 |
| stainless | 1 | 1 |
| stair | 5 | 1 |
| Stairmaster | 1 | 1 |
| stairs | 11 | 5 |
| stale | 1 | 1 |
| stance | 13 | 6 |
| stand | 7 | 7 |
| standalone | 1 | 1 |
| standard | 25 | 12 |
| standardized | 1 | 1 |
| standards | 1 | 1 |
| standing | 30 | 13 |
| standing/sitting/lying | 1 | 1 |
| standout | 1 | 1 |
| stands | 1 | 1 |
| Stanmore | 1 | 1 |
| Staple | 3 | 2 |
| staples | 7 | 5 |
| Start | 16 | 9 |
| started | 12 | 7 |
| starting | 13 | 9 |
| starts | 7 | 5 |
| Stat | 1 | 1 |
| Stata | 1 | 1 |
| state | 5 | 3 |
| stated | 2 | 2 |
| States | 7 | 5 |
| static | 10 | 5 |
| stationary | 1 | 1 |
| Statistica | 1 | 1 |
| Statistical | 11 | 4 |
| statistically | 5 | 3 |
| Statistics | 1 | 1 |
| stature | 2 | 2 |
| status | 12 | 5 |
| Staubli | 11 | 5 |
| Staubli’s | 1 | 1 |
| stay | 14 | 10 |
| stays | 2 | 1 |
| steel | 1 | 1 |
| steep | 2 | 2 |
| steeper | 1 | 1 |
| stem | 13 | 4 |
| stemmed | 3 | 2 |
| step | 11 | 6 |
| step-by-step | 1 | 1 |
| steps | 4 | 3 |
| stepwise | 3 | 3 |
| Steri-Strips | 1 | 1 |
| Steri-Strips-small | 1 | 1 |
| sterile | 6 | 3 |
| sterilization | 2 | 1 |
| Steroids | 5 | 4 |
| stick | 2 | 1 |
| stiff | 3 | 2 |
| stiffened | 1 | 1 |
| stiffness | 22 | 12 |
| still | 35 | 23 |
| stimulate | 1 | 1 |
| stimulating | 1 | 1 |
| stimulation | 2 | 1 |
| stitch | 1 | 1 |
| stitches | 2 | 1 |
| Stock | 6 | 5 |
| stocking | 4 | 2 |
| stockings | 6 | 2 |
| Stoller | 1 | 1 |
| stool | 2 | 2 |
| stop | 13 | 8 |
| stopped | 4 | 4 |
| stopping | 2 | 2 |
| stops | 2 | 2 |
| store | 2 | 2 |
| stores | 5 | 1 |
| straight | 26 | 11 |
| straight-forward | 3 | 1 |
| straighten | 12 | 7 |
| straightened | 4 | 2 |
| straightening | 3 | 3 |
| straightens | 2 | 2 |
| straightforward | 8 | 1 |
| strain | 5 | 2 |
| strains | 3 | 2 |
| strategically | 1 | 1 |
| strategy | 4 | 3 |
| strength | 38 | 17 |
| strengthen | 4 | 4 |
| strengthening | 10 | 6 |
| strenuous | 2 | 2 |
| Streptococcus | 1 | 1 |
| stress | 38 | 15 |
| stressed | 1 | 1 |
| stresses | 11 | 3 |
| stressing | 1 | 1 |
| stretch | 7 | 5 |
| stretched | 2 | 1 |
| stretcher | 1 | 1 |
| stretches | 2 | 1 |
| stretching | 2 | 2 |
| strict | 3 | 3 |
| strictly | 1 | 1 |
| stride | 2 | 2 |
| strike | 1 | 1 |
| strokes | 1 | 1 |
| strong | 24 | 10 |
| strongly | 2 | 1 |
| structural | 1 | 1 |
| structure | 7 | 6 |
| structures | 24 | 11 |
| struggle | 1 | 1 |
| struggling | 1 | 1 |
| struts | 1 | 1 |
| stuck | 1 | 1 |
| studied | 8 | 4 |
| studies | 74 | 18 |
| study | 82 | 15 |
| studying | 1 | 1 |
| stuff | 1 | 1 |
| sturdy | 2 | 2 |
| Su | 1 | 1 |
| su-perficial | 1 | 1 |
| subarticular | 1 | 1 |
| subcategorised | 1 | 1 |
| subchondral | 11 | 5 |
| subcortical | 1 | 1 |
| subcutaneous | 1 | 1 |
| subject | 2 | 2 |
| subject’s | 2 | 1 |
| subject-specific | 2 | 1 |
| subjected | 2 | 2 |
| subjective | 2 | 2 |
| subjectively | 1 | 1 |
| subjects | 4 | 3 |
| subluxation | 3 | 2 |
| submit | 1 | 1 |
| subperiosteal | 3 | 3 |
| subsequent | 12 | 7 |
| Subsequently | 7 | 7 |
| subspecialty | 1 | 1 |
| substantial | 3 | 2 |
| substantially | 2 | 2 |
| substitute | 12 | 8 |
| substitutes | 4 | 3 |
| subtle | 3 | 1 |
| succeed | 1 | 1 |
| success | 23 | 9 |
| successful | 25 | 15 |
| successfully | 3 | 3 |
| successive | 3 | 2 |
| such | 92 | 27 |
| suddenly | 1 | 1 |
| suffering | 3 | 3 |
| suffers | 1 | 1 |
| sufficient | 10 | 8 |
| sufficiently | 4 | 3 |
| suggest | 15 | 10 |
| suggested | 23 | 9 |
| suggesting | 1 | 1 |
| suggestions | 2 | 2 |
| suggestive | 1 | 1 |
| suggests | 1 | 1 |
| suitability | 2 | 1 |
| suitable | 11 | 9 |
| suited | 7 | 6 |
| sum | 1 | 1 |
| summarized | 2 | 1 |
| summary | 1 | 1 |
| sunburn | 2 | 2 |
| Superficial | 11 | 8 |
| superior | 9 | 5 |
| supervised | 1 | 1 |
| supervision | 1 | 1 |
| supine | 11 | 5 |
| supplemented | 1 | 1 |
| supply | 8 | 3 |
| supplying | 2 | 1 |
| support | 17 | 12 |
| supported | 5 | 5 |
| supporting | 1 | 1 |
| supportive | 1 | 1 |
| supposed | 4 | 3 |
| supposedly | 1 | 1 |
| suppress | 5 | 5 |
| suppressed | 1 | 1 |
| suppressing | 2 | 2 |
| suppression | 1 | 1 |
| supracondylar | 1 | 1 |
| sure | 8 | 5 |
| surface | 36 | 16 |
| surfaces | 10 | 9 |
| surgeon | 125 | 28 |
| surgeon’s | 9 | 7 |
| Surgeons | 45 | 13 |
| surgeries | 13 | 8 |
| surgery | 455 | 43 |
| surgical | 133 | 38 |
| surgically | 5 | 3 |
| surprisingly | 1 | 1 |
| surrogate | 1 | 1 |
| surround | 2 | 2 |
| surrounding | 8 | 6 |
| surveillance | 1 | 1 |
| survey | 1 | 1 |
| Survival | 42 | 13 |
| survival-rates | 1 | 1 |
| survivals | 1 | 1 |
| survivorship | 8 | 5 |
| susceptibility | 1 | 1 |
| suspected | 3 | 1 |
| sustainable | 2 | 2 |
| sustained | 3 | 2 |
| sutured | 1 | 1 |
| sutures | 2 | 2 |
| sweatpants | 1 | 1 |
| sweats | 1 | 1 |
| Sweden | 7 | 1 |
| Swedish | 5 | 1 |
| swel-ling | 1 | 1 |
| swell | 2 | 2 |
| swelling | 54 | 16 |
| swells | 1 | 1 |
| swerve | 1 | 1 |
| swimming | 2 | 1 |
| swing | 2 | 2 |
| swings | 1 | 1 |
| swiss | 3 | 2 |
| switched | 4 | 1 |
| switches | 1 | 1 |
| Switzerland | 1 | 1 |
| swollen | 3 | 3 |
| sympathetic | 2 | 2 |
| symptom | 1 | 1 |
| symptomatic | 20 | 11 |
| symptoms | 31 | 15 |
| Syndrome | 15 | 9 |
| synergistic | 1 | 1 |
| synovectomy | 1 | 1 |
| synovial | 2 | 2 |
| Synthes | 9 | 2 |
| synthetic | 3 | 3 |
| system | 27 | 14 |
| systematic | 3 | 3 |
| systemic | 2 | 2 |
| systems | 4 | 4 |
| t | 1 | 1 |
| T-and | 2 | 1 |
| T-plate | 4 | 2 |
| T-shaped | 2 | 1 |
| table | 10 | 6 |
| tablet | 2 | 2 |
| tablets | 2 | 1 |
| tactic | 1 | 1 |
| tailored | 1 | 1 |
| take | 66 | 24 |
| taken | 55 | 16 |
| takes | 26 | 12 |
| TAKEUCHI | 1 | 1 |
| taking | 17 | 13 |
| talar | 1 | 1 |
| talk | 7 | 3 |
| talked | 1 | 1 |
| talking | 5 | 2 |
| talocrural | 1 | 1 |
| tangent | 1 | 1 |
| tap | 1 | 1 |
| tape | 1 | 1 |
| tapered | 2 | 2 |
| tapes | 1 | 1 |
| target | 8 | 3 |
| targeting | 1 | 1 |
| task | 1 | 1 |
| tasks | 2 | 2 |
| taught | 7 | 5 |
| TBVA | 12 | 2 |
| TCP | 6 | 2 |
| TDWB | 3 | 1 |
| tea | 1 | 1 |
| teach | 6 | 2 |
| teaches | 1 | 1 |
| team | 6 | 3 |
| tear | 7 | 5 |
| tears | 18 | 8 |
| technical | 10 | 6 |
| technically | 5 | 5 |
| technicians | 1 | 1 |
| technique | 110 | 23 |
| techniques | 48 | 19 |
| technology | 4 | 3 |
| TED | 3 | 2 |
| telephone | 1 | 1 |
| teleradiography | 1 | 1 |
| television | 1 | 1 |
| tell | 4 | 4 |
| temperature | 3 | 3 |
| template | 2 | 1 |
| templated | 1 | 1 |
| templating | 1 | 1 |
| temporary | 6 | 5 |
| tempting | 1 | 1 |
| ten | 18 | 7 |
| ten-derness | 1 | 1 |
| ten-year | 2 | 2 |
| tend | 26 | 3 |
| tendency | 8 | 6 |
| tenderness | 4 | 3 |
| tendinitis | 2 | 2 |
| tendon | 38 | 12 |
| tendons | 5 | 4 |
| tends | 7 | 3 |
| tennis | 1 | 1 |
| tenonectomy | 1 | 1 |
| tensile | 1 | 1 |
| tension | 8 | 7 |
| tensions | 1 | 1 |
| tensor | 10 | 2 |
| term | 8 | 5 |
| terminal | 3 | 1 |
| terms | 20 | 6 |
| terrain | 1 | 1 |
| terrible | 1 | 1 |
| terribly | 1 | 1 |
| territory | 1 | 1 |
| tertiary | 1 | 1 |
| Test | 12 | 5 |
| tested | 1 | 1 |
| testing | 4 | 3 |
| tests | 6 | 4 |
| text | 2 | 1 |
| TF | 2 | 1 |
| TFA | 2 | 1 |
| than | 167 | 33 |
| that | 659 | 43 |
| that’s | 7 | 2 |
| the | 6727 | 45 |
| theatre | 5 | 2 |
| theatres | 1 | 1 |
| their | 109 | 18 |
| them | 41 | 12 |
| themselves | 2 | 2 |
| then | 146 | 30 |
| theoretical | 2 | 2 |
| Theoretically | 1 | 1 |
| theorized | 1 | 1 |
| ther | 1 | 1 |
| therapeutic | 10 | 3 |
| therapies | 1 | 1 |
| therapist | 9 | 5 |
| therapy | 28 | 14 |
| There | 164 | 36 |
| there’s | 1 | 1 |
| thereby | 18 | 8 |
| therefore | 33 | 14 |
| thereof | 1 | 1 |
| These | 126 | 29 |
| They | 184 | 24 |
| they’re | 1 | 1 |
| thicker | 2 | 1 |
| thickness | 10 | 8 |
| thigh | 32 | 13 |
| thigh-bone | 1 | 1 |
| thighbone | 18 | 10 |
| thin | 7 | 6 |
| thing | 6 | 2 |
| things | 14 | 3 |
| think | 14 | 3 |
| thinks | 1 | 1 |
| thinner | 2 | 1 |
| thinners | 2 | 1 |
| thinning | 1 | 1 |
| third | 7 | 5 |
| thirdly | 1 | 1 |
| thirds | 2 | 2 |
| thirty | 2 | 2 |
| This | 502 | 42 |
| Thisgives | 1 | 1 |
| Thomas | 1 | 1 |
| thorough | 3 | 2 |
| Thoroughly | 3 | 3 |
| those | 31 | 11 |
| though | 17 | 8 |
| thought | 11 | 5 |
| thread | 1 | 1 |
| threatening | 1 | 1 |
| three | 59 | 21 |
| Three-dimensional | 2 | 2 |
| three-quarters | 1 | 1 |
| threshold | 3 | 3 |
| thromboembolism | 1 | 1 |
| thromboprophylaxis | 1 | 1 |
| thrombosis | 18 | 11 |
| through | 107 | 30 |
| throughout | 5 | 4 |
| Throw | 1 | 1 |
| throwing | 1 | 1 |
| thrust | 18 | 10 |
| thrusts | 1 | 1 |
| Thursday | 1 | 1 |
| Thus | 18 | 7 |
| thwart | 1 | 1 |
| ti-bio-fibuler | 1 | 1 |
| tibi-al | 1 | 1 |
| tibia | 204 | 37 |
| tibia’s | 2 | 1 |
| tibiae | 1 | 1 |
| tibial | 444 | 42 |
| tibialis | 2 | 2 |
| tibio-femoral | 1 | 1 |
| tibiofemoral | 24 | 8 |
| tibiofibular | 13 | 10 |
| tibiotalar | 2 | 2 |
| tickles | 1 | 1 |
| tidy | 1 | 1 |
| tight | 4 | 2 |
| tighten | 1 | 1 |
| tightening | 2 | 2 |
| till | 2 | 2 |
| tilting | 2 | 2 |
| time | 120 | 33 |
| time-consuming | 1 | 1 |
| time-dependent | 1 | 1 |
| time-distance | 1 | 1 |
| timeline | 2 | 1 |
| times | 26 | 17 |
| Timing | 2 | 2 |
| tingling | 1 | 1 |
| tiny | 1 | 1 |
| tip | 8 | 6 |
| tipped | 1 | 1 |
| tis-sue | 1 | 1 |
| tissue | 33 | 14 |
| tissue/ligament | 1 | 1 |
| tissues | 10 | 7 |
| titanium | 10 | 6 |
| titrate | 1 | 1 |
| TKA | 36 | 7 |
| TKR | 12 | 4 |
| to | 2495 | 45 |
| tobramycin | 1 | 1 |
| Today | 3 | 1 |
| toes | 7 | 3 |
| together | 43 | 23 |
| toilet | 3 | 2 |
| toiletry | 1 | 1 |
| toilets | 1 | 1 |
| told | 5 | 3 |
| tolerable | 1 | 1 |
| tolerance | 6 | 4 |
| tolerate | 1 | 1 |
| tolerated | 8 | 4 |
| TomoFix | 42 | 7 |
| tongue | 3 | 1 |
| too | 36 | 14 |
| took | 5 | 4 |
| tool | 5 | 4 |
| tools | 1 | 1 |
| top | 20 | 7 |
| topic | 1 | 1 |
| topped | 1 | 1 |
| torn | 3 | 3 |
| torque | 1 | 1 |
| total | 105 | 33 |
| totally | 1 | 1 |
| touch | 2 | 2 |
| touch-down | 1 | 1 |
| touching | 2 | 2 |
| tourniquet | 7 | 5 |
| toward | 10 | 5 |
| towards | 17 | 9 |
| towel | 5 | 3 |
| track | 1 | 1 |
| tracker/hinge | 1 | 1 |
| tracking | 3 | 1 |
| tract | 1 | 1 |
| Trad | 1 | 1 |
| traditional | 4 | 4 |
| traditionally | 5 | 4 |
| trained | 3 | 2 |
| training | 8 | 3 |
| Tramacet | 1 | 1 |
| transected | 1 | 1 |
| transection | 3 | 1 |
| transfer | 15 | 9 |
| transfered | 1 | 1 |
| transferred | 6 | 5 |
| transferring | 10 | 10 |
| transfers | 1 | 1 |
| transition | 3 | 2 |
| translation | 15 | 8 |
| translational | 2 | 2 |
| translucent | 1 | 1 |
| transmission | 1 | 1 |
| transmits | 1 | 1 |
| transmitted | 3 | 3 |
| transosseous | 1 | 1 |
| transplant | 3 | 3 |
| transplantation | 13 | 6 |
| transplantations | 1 | 1 |
| transplants | 1 | 1 |
| transport | 1 | 1 |
| transportation | 1 | 1 |
| transported | 1 | 1 |
| transversal | 1 | 1 |
| transverse | 3 | 2 |
| trauma | 9 | 4 |
| traumatic | 5 | 4 |
| Traumatology | 1 | 1 |
| travel | 4 | 3 |
| travelling | 1 | 1 |
| traverses | 1 | 1 |
| treat | 23 | 14 |
| treated | 40 | 16 |
| Treating | 8 | 7 |
| treatment | 110 | 26 |
| treatments | 16 | 8 |
| trend | 1 | 1 |
| trends | 1 | 1 |
| trial | 5 | 5 |
| trials | 1 | 1 |
| triangle | 1 | 1 |
| triangle’s | 1 | 1 |
| triangular | 8 | 6 |
| triangularly | 1 | 1 |
| tricalcium | 2 | 1 |
| trick | 1 | 1 |
| tricks | 1 | 1 |
| tricky | 1 | 1 |
| tricompartimental | 1 | 1 |
| tricompartmental | 5 | 5 |
| tricortical | 2 | 1 |
| tried | 5 | 1 |
| trigger | 1 | 1 |
| Trillat | 1 | 1 |
| trimmed | 3 | 2 |
| triple | 4 | 1 |
| tripped | 1 | 1 |
| trivial | 1 | 1 |
| trochlear | 1 | 1 |
| trochleoplasties | 1 | 1 |
| trochleoplasty | 3 | 1 |
| trouble | 2 | 2 |
| true | 8 | 4 |
| truly | 1 | 1 |
| Trumatch | 1 | 1 |
| trunk | 2 | 2 |
| try | 7 | 4 |
| trying | 4 | 3 |
| tub | 1 | 1 |
| tube | 1 | 1 |
| tubercle | 39 | 13 |
| tuberocity | 1 | 1 |
| tuberosity | 15 | 8 |
| tubing | 1 | 1 |
| tubing/pulley | 1 | 1 |
| tune | 1 | 1 |
| tuned | 1 | 1 |
| tuning | 1 | 1 |
| tunnel | 7 | 5 |
| tunnels | 1 | 1 |
| turn | 4 | 4 |
| turned | 2 | 2 |
| turnover | 1 | 1 |
| turns | 1 | 1 |
| twenties | 1 | 1 |
| Twenty-six | 2 | 1 |
| twice | 4 | 3 |
| twist | 1 | 1 |
| twisting | 3 | 3 |
| two | 133 | 33 |
| two-dimensional | 1 | 1 |
| two-leg | 1 | 1 |
| two-part | 1 | 1 |
| two-stage | 2 | 2 |
| two-staged | 2 | 1 |
| two-thirds | 6 | 3 |
| two-year | 1 | 1 |
| twofold | 1 | 1 |
| Tylenol | 2 | 1 |
| Tylenol# | 1 | 1 |
| type | 33 | 17 |
| type/degree | 1 | 1 |
| types | 21 | 15 |
| typical | 4 | 2 |
| typically | 30 | 14 |
| U | 1 | 1 |
| U-shaped | 1 | 1 |
| UK | 7 | 3 |
| UKA | 17 | 3 |
| UKAs | 1 | 1 |
| UKR | 4 | 1 |
| Ukraine | 1 | 1 |
| ulcers | 1 | 1 |
| ultimate | 1 | 1 |
| ultimately | 6 | 6 |
| ultrasound | 1 | 1 |
| umbilical | 2 | 1 |
| un | 1 | 1 |
| un-ion | 1 | 1 |
| un-operated | 1 | 1 |
| unable | 3 | 1 |
| unaffected | 1 | 1 |
| unaided | 3 | 3 |
| unavoidable | 4 | 3 |
| unaware | 1 | 1 |
| unbalanced | 3 | 2 |
| unchanged | 3 | 2 |
| unclear | 4 | 1 |
| uncomfortable | 1 | 1 |
| uncommon | 5 | 4 |
| uncommonly | 1 | 1 |
| undamaged | 3 | 3 |
| under | 69 | 27 |
| under-corrected | 1 | 1 |
| under-correction | 1 | 1 |
| under-or | 2 | 2 |
| Undercorrection | 6 | 4 |
| undergo | 6 | 6 |
| undergoes | 1 | 1 |
| undergoing | 14 | 11 |
| undergone | 5 | 4 |
| underlined | 1 | 1 |
| underlying | 3 | 3 |
| underneath | 4 | 4 |
| understand | 5 | 3 |
| understanding | 5 | 2 |
| undertake | 2 | 2 |
| undertaken | 2 | 2 |
| undertook | 1 | 1 |
| underwent | 15 | 5 |
| undo | 1 | 1 |
| uneven | 2 | 2 |
| unevenly | 2 | 2 |
| unexpected | 1 | 1 |
| unexplained | 2 | 1 |
| unfilled | 3 | 2 |
| unfortunately | 4 | 3 |
| unhappy | 2 | 2 |
| uni-compartmental | 1 | 1 |
| unicompartimental | 3 | 2 |
| Unicompartmental | 30 | 18 |
| unicondylar | 1 | 1 |
| Unicortical | 2 | 2 |
| uniform | 1 | 1 |
| unilateral | 4 | 3 |
| uninvolved | 1 | 1 |
| union | 40 | 12 |
| uniplanar | 2 | 1 |
| Uniplane | 6 | 1 |
| unique | 1 | 1 |
| unit | 9 | 5 |
| unite | 5 | 2 |
| United | 5 | 5 |
| units | 5 | 3 |
| universal | 1 | 1 |
| University | 1 | 1 |
| unknown | 7 | 4 |
| unless | 3 | 2 |
| unlike | 4 | 3 |
| unlikely | 5 | 5 |
| unlimited | 1 | 1 |
| unload | 4 | 3 |
| unloaded | 1 | 1 |
| unloader | 2 | 2 |
| unloading | 5 | 5 |
| unloads | 5 | 5 |
| unmet | 1 | 1 |
| unnecessarily | 2 | 2 |
| unnecessary | 3 | 2 |
| unsatisfactory | 8 | 2 |
| unsophisticated | 1 | 1 |
| unstable | 17 | 8 |
| until | 43 | 19 |
| untreated | 8 | 5 |
| unusual | 1 | 1 |
| unusually | 1 | 1 |
| unwanted | 2 | 2 |
| unwell | 2 | 2 |
| up | 134 | 23 |
| up-dated | 2 | 1 |
| up-side | 2 | 1 |
| upload | 1 | 1 |
| upon | 10 | 7 |
| upper | 31 | 18 |
| upsetting | 1 | 1 |
| upwards | 1 | 1 |
| urinary | 1 | 1 |
| urine | 4 | 2 |
| us | 15 | 5 |
| USA | 12 | 5 |
| use | 102 | 29 |
| used | 183 | 34 |
| used1 | 1 | 1 |
| useful | 11 | 8 |
| user | 1 | 1 |
| users | 1 | 1 |
| uses | 8 | 7 |
| using | 121 | 31 |
| usual | 5 | 4 |
| usually | 87 | 28 |
| utilization | 1 | 1 |
| Utilize | 2 | 1 |
| utilized | 3 | 3 |
| utilizing | 1 | 1 |
| V | 2 | 1 |
| V-shaped | 2 | 2 |
| V.A. | 1 | 1 |
| V.A.C. | 2 | 1 |
| vacuum-assisted | 1 | 1 |
| valg-ise | 3 | 1 |
| valg-ising | 5 | 1 |
| valgum | 1 | 1 |
| valgus | 122 | 19 |
| valgus-producing | 3 | 3 |
| valid | 3 | 2 |
| validate | 1 | 1 |
| value | 8 | 4 |
| values | 9 | 4 |
| van | 2 | 2 |
| vancomycin | 1 | 1 |
| vancomycin-impregnated | 1 | 1 |
| var-ise | 3 | 1 |
| var-ising | 3 | 1 |
| variable | 4 | 3 |
| variables | 3 | 3 |
| variant | 1 | 1 |
| variation | 1 | 1 |
| variations | 3 | 3 |
| varied | 4 | 1 |
| varies | 2 | 2 |
| variety | 9 | 8 |
| various | 12 | 7 |
| varum | 3 | 2 |
| varus | 184 | 29 |
| varus-aligned | 1 | 1 |
| varus/ | 1 | 1 |
| vary | 3 | 2 |
| VAS | 4 | 2 |
| vascular | 2 | 2 |
| vegetables | 1 | 1 |
| vein | 19 | 11 |
| veins | 1 | 1 |
| Velcro | 1 | 1 |
| velcroed | 1 | 1 |
| velcros | 1 | 1 |
| venous | 4 | 3 |
| ver | 1 | 1 |
| verification | 1 | 1 |
| verified | 2 | 1 |
| verify | 3 | 3 |
| verifying | 1 | 1 |
| version | 2 | 2 |
| versions | 1 | 1 |
| versus | 5 | 4 |
| vertical | 5 | 4 |
| very | 108 | 12 |
| vessel | 4 | 4 |
| vessels | 8 | 7 |
| veterinary | 1 | 1 |
| vi-sualization | 1 | 1 |
| via | 4 | 2 |
| viable | 3 | 3 |
| vice-versa | 1 | 1 |
| vicious | 1 | 1 |
| video | 2 | 2 |
| view | 19 | 8 |
| viewed | 3 | 2 |
| viewing | 1 | 1 |
| views | 28 | 8 |
| vigorous | 1 | 1 |
| Vilensky | 1 | 1 |
| violation | 1 | 1 |
| Vioreanu | 2 | 1 |
| Vioreanu’s | 1 | 1 |
| virtual | 2 | 1 |
| virtually | 3 | 1 |
| visible | 2 | 2 |
| vision | 3 | 2 |
| visit | 9 | 8 |
| visitors | 1 | 1 |
| visits | 1 | 1 |
| Visual | 7 | 5 |
| visualisation | 1 | 1 |
| visualising | 1 | 1 |
| visualization | 1 | 1 |
| Visualize | 2 | 2 |
| visualized | 1 | 1 |
| visualizes | 1 | 1 |
| visually | 1 | 1 |
| vital | 8 | 5 |
| vivo | 6 | 1 |
| vogue | 1 | 1 |
| Voltaren | 2 | 2 |
| Voltarol | 1 | 1 |
| volume | 2 | 2 |
| vomiting | 2 | 2 |
| vs | 4 | 2 |
| vs. | 1 | 1 |
| W-Dahl | 1 | 1 |
| waist | 4 | 4 |
| Wait | 1 | 1 |
| waiting | 2 | 2 |
| wake | 6 | 3 |
| waking | 1 | 1 |
| walk | 27 | 11 |
| walked | 1 | 1 |
| walkers | 1 | 1 |
| walking | 32 | 17 |
| walks | 1 | 1 |
| walkways | 1 | 1 |
| wall | 5 | 3 |
| Wallrichs | 2 | 1 |
| want | 33 | 6 |
| wanted | 3 | 2 |
| wants | 2 | 1 |
| ward | 6 | 3 |
| warm | 1 | 1 |
| warmth | 3 | 2 |
| warned | 1 | 1 |
| warns | 1 | 1 |
| warranted | 1 | 1 |
| wary | 1 | 1 |
| was | 367 | 24 |
| wash | 4 | 4 |
| washing | 1 | 1 |
| washout | 3 | 2 |
| wasn’t | 2 | 1 |
| waste | 1 | 1 |
| wasting | 2 | 2 |
| watch | 2 | 1 |
| watching | 1 | 1 |
| water | 7 | 6 |
| waterproof | 5 | 1 |
| Waugh | 2 | 2 |
| way | 30 | 11 |
| ways | 5 | 4 |
| WB | 1 | 1 |
| WBL | 9 | 3 |
| We | 228 | 18 |
| We’ll | 2 | 1 |
| we’ve | 2 | 1 |
| weak | 3 | 1 |
| weakened | 1 | 1 |
| weaker | 1 | 1 |
| weakness | 2 | 2 |
| wealth | 1 | 1 |
| wear | 39 | 16 |
| wear-and-tear | 2 | 1 |
| wearing | 7 | 6 |
| wears | 7 | 4 |
| website | 1 | 1 |
| wedge | 299 | 38 |
| wedge-shaped | 10 | 7 |
| wedged | 3 | 2 |
| wedged-open | 1 | 1 |
| wedges | 4 | 3 |
| wedging | 1 | 1 |
| week | 22 | 10 |
| weekend | 2 | 1 |
| weekly | 1 | 1 |
| weeks | 144 | 31 |
| weigh | 1 | 1 |
| weighbearing | 1 | 1 |
| weighs | 1 | 1 |
| weight | 150 | 36 |
| weight-bear | 4 | 3 |
| weight-bearing | 80 | 15 |
| Weightbearing | 13 | 6 |
| weights | 1 | 1 |
| Welfare | 2 | 1 |
| well | 78 | 26 |
| well-balanced | 1 | 1 |
| well-established | 3 | 3 |
| well-maintained | 2 | 2 |
| well-positioned | 1 | 1 |
| well-tolerated | 2 | 2 |
| went | 6 | 1 |
| were | 201 | 18 |
| wet | 2 | 1 |
| what | 41 | 11 |
| whatever | 4 | 2 |
| wheelchair | 1 | 1 |
| when | 191 | 39 |
| whenever | 1 | 1 |
| where | 80 | 25 |
| whereas | 17 | 8 |
| whereby | 1 | 1 |
| whether | 25 | 16 |
| which | 212 | 35 |
| While | 58 | 23 |
| whilst | 8 | 4 |
| white | 4 | 3 |
| who | 131 | 27 |
| whole | 14 | 7 |
| whom | 3 | 3 |
| whose | 3 | 2 |
| why | 8 | 5 |
| wide | 4 | 4 |
| widely | 12 | 10 |
| widen | 2 | 2 |
| wider | 4 | 3 |
| widespread | 1 | 1 |
| widest | 1 | 1 |
| width | 26 | 10 |
| Wilcoxon | 1 | 1 |
| will | 391 | 33 |
| willing | 1 | 1 |
| Wilson | 1 | 1 |
| Windows | 1 | 1 |
| windsurfing | 1 | 1 |
| windswept | 1 | 1 |
| wing | 1 | 1 |
| wire | 2 | 2 |
| wires | 16 | 9 |
| wise | 2 | 2 |
| wish | 3 | 3 |
| wishes | 1 | 1 |
| wishing | 1 | 1 |
| with | 1073 | 45 |
| with/without | 1 | 1 |
| within | 33 | 13 |
| without | 83 | 28 |
| withstand | 1 | 1 |
| WLR | 3 | 1 |
| wobble | 1 | 1 |
| woman | 1 | 1 |
| Women | 5 | 4 |
| Women’s | 1 | 1 |
| won’t | 3 | 1 |
| wont | 1 | 1 |
| Woodacre | 2 | 2 |
| woozy | 1 | 1 |
| word | 1 | 1 |
| words | 1 | 1 |
| work | 51 | 14 |
| work/sports | 1 | 1 |
| worked | 6 | 1 |
| workers | 2 | 1 |
| working | 7 | 2 |
| workload | 1 | 1 |
| workouts | 1 | 1 |
| works | 5 | 2 |
| world | 5 | 3 |
| world-renowned | 2 | 2 |
| worldwide | 4 | 3 |
| worn | 16 | 12 |
| worried | 1 | 1 |
| worry | 3 | 1 |
| worse | 12 | 7 |
| worsened | 3 | 3 |
| worsening | 2 | 2 |
| worsens | 5 | 3 |
| worst | 1 | 1 |
| worst-case | 2 | 1 |
| worth | 1 | 1 |
| worthy | 2 | 2 |
| would | 51 | 16 |
| wouldn’t | 1 | 1 |
| wound | 59 | 16 |
| wounds | 2 | 2 |
| wrap | 2 | 1 |
| wrapped | 1 | 1 |
| wrapping | 1 | 1 |
| Write | 1 | 1 |
| written | 2 | 2 |
| wrong | 3 | 2 |
| wrote | 1 | 1 |
| X | 2 | 1 |
| x-ray | 30 | 10 |
| x-rays | 37 | 12 |
| Xor | 1 | 1 |
| year | 42 | 17 |
| years | 180 | 30 |
| years,4-6 | 1 | 1 |
| Yes | 2 | 2 |
| yet | 3 | 2 |
| yielded | 1 | 1 |
| Yoshino | 1 | 1 |
| you | 610 | 19 |
| you'll | 4 | 1 |
| you’re | 1 | 1 |
| you’ve | 10 | 1 |
| young | 45 | 19 |
| younger | 42 | 18 |
| youngest | 1 | 1 |
| your | 507 | 22 |
| yours | 1 | 1 |
| yourself | 7 | 4 |
| youthful | 1 | 1 |
| Zheng | 2 | 1 |
| Zimmer | 1 | 1 |
| zone | 9 | 3 |
| zones | 1 | 1 |

**Sentences Breakdown**

**Long Sentences**

| Website No. | Overly-long Sentences | Longest Sentence Length | Longest Sentence |
| --- | --- | --- | --- |
| 1 | 9 | 33 | But in certain patients, a high tibial osteotomy can realign the knee to take pressure off the damaged side by wedging open the upper portion of the tibia to reconfigure the knee joint. |
| 2 | 14 | 29 | In some cases, rather than "closing" the bones, the wedge of bone is "opened" and a bone graft is added to fill the space and help the osteotomy heal. |
| 3 | 74 | 69 | Jakob and Jacob suggested that correction of the mechanical axis depends on the thickness of the cartilage in the medial compartment: if one third of the medial cartilage is lost, the mechanical axis should pass 10-15% lateral from the center of the tibial plateau; if two thirds of the cartilage is lost, the axis should pass 20-25% lateral; and if all is lost, the axis should pass 30-35% lateral. |
| 5 | 57 | 59 | The most common type of knee osteotomy is a high tibial medial opening wedge osteotomy, a surgery that adds a wedge of bone graft or substitute to the inner shin bone, just below the knee.2 It is typically done for bowlegged patients, relieving pressure on the inside of the knee and transferring it to the outside of the knee. |
| 6 | 10 | 39 | Removing or adding a wedge of bone in your upper shinbone or lower thighbone can help straighten the bowing, shift your weight to the undamaged part of your knee joint and prolong the life span of the knee joint. |
| 7 | 79 | 75 | According to Jakob et al., they also suggested that correction of the mechanical axis should depend on the thickness of the remaining medial compartment cartilage: if 1/3 of medial cartilage is lost, the mechanical axis should pass 10– 15% laterally from the center of the tibial plateau; if 2/3 of the cartilage is lost, the axis should pass 20– 25% laterally, and if almost all the cartilage is lost, it should pass 30– 35% laterally. |
| 8 | 74 | 59 | Lerner et al. found that each 1° of TF alignment deviation altered the first peak medial KCF by 51  N, whilst each 1  mm of medial-lateral translation of the compartment contact point position altered the first peak medial KCF by 41  N. KCF can be used to identify early knee OA development prior to the onset of radiographic evidences. |
| 9 | 39 | 41 | Removing just a small wedge of bone can “swing” your knee open, pressing the healthy tissue together as space opens up between the thighbone and shinbone on the damaged side so that the arthritic surfaces do not rub against each other. |
| 10 | 36 | 52 | In our hospital, if a 2-stage exchange is used, we do not do the free interval without antibiotics before reimplantation of prosthesis and we consider the alternatives of short and long intervals of time, depending on the soft tissue situation, the bacteria found on the microbial work up, and their resistance patterns. |
| 11 | 9 | 38 | You must contact our office before you go into hospital if there is any evidence of pimples, ulcers or broken skin around the area to be operated on OR if you have a cold, cough or infection evident. |
| 12 | 8 | 49 | If, at 1 year after the operation, the valgus angulation of the anatomic axis (femorotibial angle) is less than 8 degrees or if the patient’s weight is more than 1.32 times the ideal body weight, then survivorship decreases to 38% at 5 years and to 19% at 10 years. |
| 13 | 21 | 48 | To reduce swelling: elevate the leg on pillows to avoid keeping the foot down for the first few days after surgery or wrap your leg with a snug tensor bandage, starting at the toes and working your way up above the knee to the middle of the thigh. |
| 14 | 12 | 47 | Lines and drains -When you return to your ward you may still need oxygen, will have IV lines for fluid and pain medication and you may have a catheter to help keep your bladder empty until you are able to manage getting up to go the toilet. |
| 15 | 4 | 44 | The advantages over the conventional techniques are reducing the chance of accidental intra-articular extension of the osteotomy, eliminating the potential risk of unwanted translation or rotation of the bone, ability to readjust the alignment after surgery without the need to remove the bone screws. |
| 16 | 18 | 40 | After the wedge has been created, a piece of bone graft is either harvested from the illiac crest of the pelvis, or a bone graft substitute is added to the gap to hold open the space and realign the knee. |
| 17 | 146 | 71 | Particularly osteotomies around the knee such as supracondylar femoral and high tibial osteotomies (HTO) are appreciated as treatment choices for younger patients with unilateral OA, allowing for long-term preservation of the knee joint that may reduce the need for total knee replacement (TKR), as European Arthroplasty Registers show that up to 20% of all patients with TKR are not satisfied with their knee function, mainly because of residual stiffness and pain. |
| 18 | 9 | 38 | It is usually performed in arthritic conditions affecting only one side of your knee and the aim is to take pressure off the damaged area and shift it to the other side of your knee with healthy cartilage. |
| 19 | 89 | 57 | Correction of the coronal plane In the past, correction of the coronal plane was made to 8o of the anatomic valgus, as suggested by Coventry et al., and 6 of the mechanical valgus, as suggested by Hernigou et al. More recently, guidelines have changed and correction of the coronal plane is made according to the weight-bearing axis. |
| 20 | 15 | 68 | Complete recovery after HTO, defined as pain-free return to full activity, including unlimited exercise can take up to 6 months or longer.3 Data on limb alignment, rate of bone union, time to return to weight-bearing, walking speed, stride length, kinematics, dynamic knee joint load and patient-reported measures of pain, function, and quality of life at six, 12, 18 and 24 months postoperative HTO (and longer) has been published. |
| 21 | 31 | 51 | This, together with advances made in knee replacement procedures, has meant that HTO has been progressively abandoned, which could also be the motivation for finding new indications for HTO procedures, as studies began to look to HTO not for the treatment of advanced arthritis, but for treatment at the initial stages. |
| 22 | 14 | 38 | It is usually performed in arthritic conditions affecting only one side of your knee and the aim is to take pressure off the damaged area and shift it to the other side of your knee with healthy cartilage. |
| 23 | 18 | 59 | Differences in the clinical outcome between total knee arthroplasty following high tibial osteotomy and total knee arthroplasty without prior high tibial osteotomy are unknown according to El-Galaly et al. After adjustment for male sex and younger age, total knee arthroplasty after high tibial osteotomy for medial compartment osteoarthritis had a survival similar to that of primary total knee arthroplasty. |
| 24 | 78 | 87 | HTO is usually contraindicated in patients older than 65 years or with severe OA of the medial compartment (Ahlback grade III or higher), tricompartmental OA, relevant patellofemoral OA, ROM <120° (flexion less than 90° and a flexion contracture greater than 10°), joint instability and ≥1 cm lateral tibial thrust, ≥20° of malalignment; other contraindications are axial deformity correctable by physical examination and assessed on stress radiographs, diagnosed inflammatory arthritis, large area of exposed bone on tibial and femoral articular surface (>15 cm ×15 mm) and heavy smokers. |
| 25 | 6 | 38 | It is usually performed in arthritic conditions affecting only one side of your knee and the aim is to take pressure off the damaged area and shift it to the other side of your knee with healthy cartilage. |
| 26 | 17 | 55 | The preoperative 3-D simulation allows the surgeon to plan the osteotomy strictly at the bony level by using the medial proximal tibial angle instead of traditionally used planning angles, such as the medial femorotibial angle and weight bearing line percentage that span the entire leg and eventually can be misinterpreted due to soft tissue laxity. |
| 27 | 8 | 44 | Dr. Jonathan Glashow, a NYC board certified orthopedic surgeon, will adjust the alignment of your lower leg (tibia) and knee so your body weight is shifted toward a healthier part of the cartilage of your knee alleviating your pain or to correct a deformity. |
| 28 | 11 | 34 | One of the simply things to test if the patient is a good fit for an osteotomy, test if he is able to fully straighten the knee and bend it at least 90 degrees. |
| 29 | 7 | 26 | Instead of closing the wedge, the wedge of bone is “opened” and a bone graft is added to fill the space and help the osteotomy heal. |
| 30 | 8 | 41 | In this procedure the surgeon makes an incision in front of the knee just below the knee cap to remove a small wedge of bone from the upper part of the tibia or shin bone on the medial or inner side. |
| 31 | 39 | 43 | High tibial osteotomy is best utilized when the patient is a non-smoker, has body weight less than 1.32 times normal, ROM in the affected knee is greater or equal to 90 degrees, and when the arthritic changes are limited to the medial compartment. |
| 32 | 46 | 57 | Compared to the youngest age group (30– 39 years), the risk of revision increased in the older age groups, with RR =2 (CI: 1.3– 3.3) in the 40-to 49-year age group, RR =2.7 (CI: 1.8– 4.1) in the 50-to 59-year age group, and RR =2.5 (CI: 1.6– 4) in patients who were 60 years old or more. |
| 33 | 11 | 38 | The Beverly Hills orthopedic surgeon corrects this by realigning the angle made between the bones of the leg, so the patient can shift his or her body weight, allowing the healthy side to assume the majority of stress. |
| 34 | 46 | 78 | Nakamura et al., compared 28 closing wedge osteotomies versus 40 dome osteotomies, with average varus alignment for each was 5.67 ̊and 1.45 ̊,respectively recorded subjective satisfac-tion scores where 42.9% patients were unhappy with the closing wedge osteoto-my compared with 57.5% with the dome osteotomy With the advent of biplanar technique in OWHTO Certain issues concerned including enhancement of os-teotomy healing, sagittal inclination of tibial plateau, preservation of patellar height together with secondary alteration of the normal knee kinematics. |
| 35 | 57 | 76 | The analysis of the effect of the size of the correction angle on HTO outcomes is described by Floerkemeier S et al. and Nelissen EM et al. In the first study it is concluded that the size of the correction angle does not have any effect on HTO result, and the second one showed that an osteotomical wedge size exceeding 10° is associated with significantly higher number of complications than a wedge size less than 10°. |
| 36 | 4 | 30 | If one part of the joint is worn out, the angle of the leg bone can often be changed to shift stresses onto other areas that are not so worn. |
| 37 | 10 | 35 | An appointment will be made for two weeks after your surgery, but if you have any problems before then, it is important you speak to your GP or the clinic where your surgery was performed. |
| 38 | 3 | 33 | During a tibial osteotomy your surgeon will adjust the alignment of your lower leg (tibia) and knee so your body weight is shifted toward a healthier part of the cartilage of your knee. |
| 39 | 318 | 87 | Now that doesn’t mean that we don’t do knee replacements in these patients (indeed, they are sometimes the only option), and it doesn’t mean that we don’t have success in this younger age group, but what follow-up studies have shown us is that the failure rate is much much higher in a young active patient versus that sedentary patient who is 65, because in the former group their pain goes and they want to be active again, and the knee replacement doesn’t cope with that terribly well. |
| 40 | 30 | 49 | The physiotherapy team will continue to work with you as an outpatient and they provide us with a report at the 6 week mark post op. We will see you in clinic at the 3 month mark and perform repeat x rays of the limb to assess the correction. |
| 41 | 7 | 35 | Your orthopedic surgeon will cut and alter the alignment or share of your leg bone in order to shift weight from the damaged area of the knee to the healthier side of the knee joint. |
| 42 | 54 | 48 | Patients complaining of symptoms of pain and “giving way” or instability during activities of daily living can be effectively treated with HTO alone, whereas patients complaining of both pain and instability primarily during pivoting sports are often better treated with realignment surgery in conjunction with a ligament reconstruction. |
| 43 | 11 | 40 | You will be able to leave hospital once you are able to walk safely with an appropriate mobility aid, move on your own between sitting and standing, and complete the prescribed exercises on your own, as outlined by your physiotherapist. |
| 44 | 7 | 34 | High tibial osteotomy is a surgical procedure to realign the leg and reduce the pain you have from your knee by transferring the body weight to the preserved normal outer side of the knee. |
| 45 | 52 | 77 | Mechanical axis (line from the center of the femoral head to the center of the knee), anatomical axis (a line from the piriformis fossa to the center of the knee joint and a line through the long axis of the tibia), and weight bearing axis (line drawn from the center of the femoral head to the center of ankle joint) are measured on the alignment view, where the location, type, and amount of corrective osteotomy is determined. |

**Wordy Items**

| Website No. | Frequency | Wordy Items | Suggestions |
| --- | --- | --- | --- |
| 1 | 23 | "ability", "benefits", "cartilage" * 4, "develop", "generally", "in conjunction with", "in order to", "known as", "located", "necessary", "performed", "portion" * 3, "procedure" * 2, "procedures", "result in", "typically" * 2 | "skill", "helps", "gristle", "make, grow", "broadly", "along with, and, combined with, coupled with, joined with, paired with, with", "to, for", "called, named", "found", "needed", "did/done", "part", "rule, way, method", "rules, ways, methods", "lead to", "often" |
| 2 | 43 | "advantage" * 2, "as a result", "as well as", "available", "cartilage", "created", "creates", "determine", "develop", "difficult", "evaluate", "function", "general", "however", "in most cases", "in order to", "in some cases" * 3, "incision", "maintain", "monitored", "operated", "option", "outside of", "participate", "performed", "procedure" * 7, "remains", "require", "required" * 2, "review", "typically" * 2, "usually" | "plus", "so, then, thus", "and, also", "offered, ready", "gristle", "made", "makes", "decide, figure", "make, grow", "hard", "check, rate", "act, role", "broad", "but", "mostly, most of these, often, usually", "to, for", "at times, sometimes", "cut", "keep, support", "checked, watched", "ran, worked", "choice, way", "outside (unless proceeding a pronoun)", "take part", "did/done", "rule, way, method", "stays", "need", "needed", "check", "often", "often" |
| 3 | 230 | "accordingly" * 2, "accuracy", "achieve", "achieved" * 3, "achieving" * 2, "additionally", "adequate", "adjacent to", "advantageous", "advantages" * 2, "aggressive", "anterior" * 7, "appropriate" * 6, "augmentation", "available", "beneficial", "bilateral", "cartilage" * 5, "combined" * 2, "commenced", "component", "contribute", "contributed", "contributes", "conversion" * 5, "demonstrate", "desired" * 2, "determined", "develop", "difficult", "difficulty", "disrupt", "elevated", "elevation", "employment", "encountered", "ensure" * 3, "evaluated", "examination", "external" * 6, "first introduced", "frequently", "function" * 2, "general", "gives rise to", "have an influence on", "however" * 6, "identical to", "identified", "identifying", "immediately" * 2, "in a similar fashion", "in order to", "in terms of", "incision" * 8, "indicated", "indication" * 2, "individual", "inferior" * 2, "initiated", "insufficient" * 2, "internal" * 3, "is defined as", "is in line with", "located" * 2, "location", "maintain" * 2, "maintained" * 2, "minimize", "modified", "multiple" * 2, "necessary", "necessitate" * 2, "observed", "obtained", "on a regular basis", "on the other hand" * 2, "operating", "performed" * 16, "portion", "position" * 2, "positioned", "posterior" * 14, "previous", "primary" * 2, "prior to", "procedure" * 12, "procedures", "produces", "rapid", "recommended" * 3, "reduce" * 4, "reduced", "reduces", "reducing", "reduction" * 4, "remain", "remaining", "remains", "require" * 2, "required", "requires", "result in" * 5, "results in", "subsequent" * 3, "subsequently", "taken into consideration", "the state of", "therefore" * 3, "visualizes", "whereas" * 4 | "so, just so", "correctness, exactness", "do, make", "did, mad", "doing, making", "added, more", "enough", "close to, near, next to, beside, by", "helpful", "pluses", "forward, strong, attacking", "front", "proper (adj.), set aside (verb)", "increase", "offered, ready", "helpful", "two-sided", "gristle", "joined", "began", "part", "give, help", "gave, helped", "gives, helps", "change", "show", "wished", "decided, figured", "make, grow", "hard", "trouble", "interrupt, confuse", "rose, lifted up", "height", "work, job, use", "met", "make sure", "checked, rated", "check", "outer", "introduced", "often", "act, role", "broad", "causes, leads to, results in", "affect, influence", "but", "the same as", "named, found", "naming, finding", "at once, right away, right now", "like", "to, for", "as for", "cut", "shown", "clue, sign", "person, single", "lesser", "started", "not enough", "inner, inside", "is", "conforms with", "found", "place", "keep, support", "kept, supported", "decrease, lessen", "changed", "many", "needed", "cause, need", "saw, seen", "got", "regularly", "however", "running, working", "did/done", "part", "place", "placed", "rear", "earlier, past", "main, first", "before", "rule, way, method", "rules, ways, methods", "makes", "quick", "suggested", "cut", "cut", "cuts", "cutting", "cut", "stay", "staying, left over", "stays", "need", "needed", "needs", "lead to", "leads to", "later, next", "later, after", "considered", "Consider removing this", "so, thus", "pictures", "since" |
| 4 | 27 | "accelerate", "achieved", "advantages" * 3, "anterior", "benefit", "benefits", "cartilage" * 2, "combined", "general", "indications" * 3, "inferior", "located", "maintain" * 2, "maintaining", "oblique", "posterior" * 2, "procedure", "procedures", "required", "via" | "hasten, quicken", "did, mad", "pluses", "front", "help", "helps", "gristle", "joined", "broad", "clues, signs", "lesser", "found", "keep, support", "keeping, supporting", "slanting", "rear", "rule, way, method", "rules, ways, methods", "needed", "in, on, by" |
| 5 | 137 | "ability" * 2, "actual" * 2, "adjustments", "administered", "advantages", "all of" * 2, "anterior", "anxiety", "approval", "as a result of", "benefit" * 2, "cartilage" * 7, "comprise", "continue", "creating", "determine", "difficult", "elect", "electing", "elevated" * 4, "eliminate" * 4, "ensure" * 3, "facilitates", "frequently", "function" * 5, "general" * 4, "generally" * 2, "impact" * 2, "in conjunction with", "in order to", "incision" * 2, "incisions", "individuals who", "initially", "is comprised of", "located", "location", "modifications", "modified", "monitor", "necessary" * 2, "notify", "obtain", "operate", "option" * 4, "outside of" * 2, "over and over again", "participate", "perform" * 2, "performed" * 3, "performing", "permitted", "physician", "position", "preparation", "primary" * 2, "prior to" * 2, "procedure", "proceed", "proficient", "program", "recommended" * 5, "reduce" * 7, "reducing", "regimen", "remain", "remaining", "require" * 3, "required" * 3, "requires" * 2, "similar", "therefore" * 3, "typically" * 6, "usually" * 4 | "skill", "real", "settlements", "managed", "pluses", "all (unless proceeding a pronoun), Consider removing this", "front", "fear", "praise, consent", "because of, due to, following", "help", "gristle", "form, include", "keep, keep on", "making", "decide, figure", "hard", "choose, pick", "choosing, picking", "rose, lifted up", "cut, drop", "make sure", "eases, helps", "often", "act, role", "broad", "broadly", "hit, change", "along with, and, combined with, coupled with, joined with, paired with, with", "to, for", "cut", "cuts", "those who", "at first", "comprises", "found", "place", "changes", "changed", "check, watch", "needed", "let know, tell", "get", "run, work", "choice, way", "outside (unless proceeding a pronoun)", "repeatedly", "take part", "do", "did/done", "doing", "let", "doctor", "place", "readiness", "main, first", "before", "rule, way, method", "do, go on", "expert, skilled", "plan", "suggested", "cut", "cutting", "routine, rule", "stay", "staying, left over", "need", "needed", "needs", "like", "so, thus", "often", "often" |
| 6 | 25 | "appropriate", "approximately", "as long as", "cartilage" * 4, "creating", "determine", "general", "in most cases", "incision", "incomplete", "location", "option", "performed" * 2, "portion", "procedure" * 4, "recommend", "typically", "usually" | "proper (adj.), set aside (verb)", "about", "if, since", "gristle", "making", "decide, figure", "broad", "mostly, most of these, often, usually", "cut", "partial", "place", "choice, way", "did/done", "part", "rule, way, method", "suggest", "often", "often" |
| 7 | 235 | "absolutely", "accurately", "achieved" * 5, "adapt", "almost all", "analysis", "anterior" * 2, "appropriate", "at the time" * 3, "available", "bilateral" * 2, "cartilage" * 52, "combined", "composed" * 2, "comprised", "concluded" * 4, "conversion" * 4, "criteria" * 2, "definitive", "determine", "determined" * 3, "deviation" * 2, "difficult" * 4, "difficulties" * 2, "difficulty", "equivalent", "established", "evaluate" * 3, "evaluated" * 3, "evaluating" * 2, "evaluation" * 5, "final" * 5, "have a tendency", "however" * 4, "in order to" * 4, "in spite of", "in the case of", "incision" * 2, "indicated" * 3, "institution", "investigated", "limitation" * 2, "limitations", "located", "maintain" * 2, "necessary", "not possible", "observed", "obtain" * 3, "obtained" * 4, "performed" * 11, "permitted" * 2, "position" * 3, "posterior" * 5, "previous", "primarily", "prior to", "procedure" * 3, "procedures" * 30, "rapid", "recommended" * 3, "remaining", "required" * 2, "result in", "resulted in", "retained", "review", "reviewed", "simultaneously", "strategy", "subsequently", "sufficient", "therefore" * 3, "usually" * 3, "was comprised of", "whereas" * 2 | "wholly", "correctly, exactly", "did, mad", "make fit", "most", "review, breakdown, exam, study", "front", "proper (adj.), set aside (verb)", "when", "offered, ready", "two-sided", "gristle", "joined", "made up, created, calm (adj.)", "formed, included", "closed, ended", "change", "requirements", "final", "decide, figure", "decided, figured", "change", "hard", "troubles", "trouble", "equal", "set up, proved", "check, rate", "checked, rated", "checking, rating", "check, rating", "last", "tend to", "but", "to, for", "aside, despite, although", "in, with, if, by, for (or delete)", "cut", "shown", "office, company, school", "reviewed, checked, looked over", "limit", "limits", "found", "keep, support", "needed", "impossible", "saw, seen", "get", "got", "did/done", "let", "place", "rear", "earlier, past", "mainly, firstly", "before", "rule, way, method", "rules, ways, methods", "quick", "suggested", "staying, left over", "needed", "lead to", "lead to", "kept, held", "check", "checked", "at the same time", "plan", "later, after", "enough, ample", "so, thus", "often", "comprised", "since" |
| 8 | 232 | "a large number of", "ability", "accompanied", "accuracy" * 2, "accurate" * 2, "accurately", "achieve" * 2, "achieved", "achieves", "achieving", "acquired", "adapt", "adequate", "advantages" * 4, "advocated", "almost all", "alternative", "analysis" * 11, "anterior", "appropriate" * 3, "approximately" * 3, "as long as", "as well as" * 3, "ascending", "available", "cartilage" * 21, "characterize", "characterized" * 2, "combining", "concluded", "conclusive", "consequently" * 2, "construct" * 2, "contributed", "criteria", "descending", "determined" * 2, "determining", "developing", "deviation" * 2, "difficulties", "established", "evaluate", "evidences" * 2, "examine", "explain", "external", "final", "function" * 3, "general", "generally", "however" * 17, "identified", "identify" * 2, "illustrates", "in spite of", "in terms of" * 3, "incision", "incorporated", "indication", "indications", "individual", "insufficient" * 3, "internal", "investigate" * 3, "investigated" * 4, "investigating", "is dependent on" * 2, "limitations" * 2, "maintain" * 2, "maintained", "majority" * 2, "maximum", "methodologies", "modification", "modifications", "modified" * 2, "modifying", "necessarily", "necessary" * 2, "necessitates", "objective", "observing", "obtain", "obtained", "on the contrary", "operated", "performed" * 2, "position" * 5, "possess", "posterior" * 2, "predominantly", "preparation", "previous" * 3, "primary" * 3, "prior to", "probability", "procedure" * 4, "produced", "produces" * 3, "reason why", "recommended" * 2, "reduce", "reduced" * 3, "reduces", "reducing" * 2, "remain", "remains" * 3, "result in", "resulted in" * 2, "resulting in", "results in" * 4, "review", "similar", "substantial", "substantially", "sufficient", "superior", "the majority of", "therefore" * 2, "took into account", "transmitted", "variations", "whereas" | "numerous, many", "skill", "went with", "correctness, exactness", "correct, exact", "correctly, exactly", "do, make", "did, mad", "does, makes", "doing, making", "gained, got", "make fit", "enough", "pluses", "spoke for", "most", "choice", "review, breakdown, exam, study", "front", "proper (adj.), set aside (verb)", "about", "if, since", "and, also", "climbing, upward", "offered, ready", "gristle", "describe", "described", "joining", "closed, ended", "final", "so", "build", "gave, helped", "requirements", "downward", "decided, figured", "deciding, figuring", "making, growing", "change", "troubles", "set up, proved", "check, rate", "shows", "check, look at", "show, tell", "outer", "last", "act, role", "broad", "broadly", "but", "named, found", "name, find", "draws, shows", "aside, despite, although", "as for", "cut", "blended, joined, mixed", "clue, sign", "clues, signs", "person, single", "not enough", "inner, inside", "review, check, look over", "reviewed, checked, looked over", "reviewing, checking, looking over", "depends on, hinges on", "limits", "keep, support", "kept, supported", "most", "most, greatest", "methods, designs, plans", "change", "changes", "changed", "changing", "needed, needed to", "needed", "causes, needs", "aim, goal", "seeing", "get", "got", "rather, instead", "ran, worked", "did/done", "place", "have, own", "rear", "superiorly", "readiness", "earlier, past", "main, first", "before", "chance", "rule, way, method", "made", "makes", "reason", "suggested", "cut", "cut", "cuts", "cutting", "stay", "stays", "lead to", "lead to", "leading to", "leads to", "check", "like", "real, strong, large", "really, strongly, largely", "enough, ample", "better, boss", "most, most of", "so, thus", "considered", "sent", "changes, differences", "since" |
| 9 | 121 | "adequate", "advantage", "all of", "anxiety", "appear", "as a result", "as well as" * 2, "assistance", "cartilage" * 7, "continue" * 2, "continued", "created", "creates", "creating", "determine" * 3, "determines", "develop", "difficult" * 2, "elevated", "ensure", "final", "frame of mind", "general", "generally" * 4, "impact" * 2, "in conjunction with", "in order to", "in some cases" * 2, "incision" * 3, "maintain" * 2, "maintaining", "monitor", "monitored" * 2, "multiple", "necessary" * 2, "operating" * 2, "option", "outside of", "participate", "perform" * 4, "performed" * 2, "physician" * 6, "physicians" * 6, "portion", "position", "procedure" * 8, "program", "recommend" * 5, "reduce" * 3, "remain" * 2, "remains" * 2, "require", "required", "resulting in", "typically" * 4, "usually" * 12 | "enough", "plus", "all (unless proceeding a pronoun), Consider removing this", "fear", "seem, come", "so, then, thus", "and, also", "help", "gristle", "keep, keep on", "kept on", "made", "makes", "making", "decide, figure", "decides, figures", "make, grow", "hard", "rose, lifted up", "make sure", "last", "attitude, posture, view, viewpoint", "broad", "broadly", "hit, change", "along with, and, combined with, coupled with, joined with, paired with, with", "to, for", "at times, sometimes", "cut", "keep, support", "keeping, supporting", "check, watch", "checked, watched", "many", "needed", "running, working", "choice, way", "outside (unless proceeding a pronoun)", "take part", "do", "did/done", "doctor", "doctors", "part", "place", "rule, way, method", "plan", "suggest", "cut", "stay", "stays", "need", "needed", "leading to", "often", "often" |
| 10 | 100 | "adapted", "adjacent to", "alternatives", "analysis", "appears", "appropriate", "as well as", "attempt", "benefit", "cartilage", "category", "collection" * 2, "combined" * 3, "concerning" * 4, "concluded", "consolidation", "containing", "develop", "developed", "elevated", "equivalent", "evaluating", "examination", "exchange", "explain" * 2, "external" * 4, "final", "function", "hazardous", "however" * 7, "identified" * 2, "immediately", "in order to", "incomplete", "indication" * 2, "initial" * 5, "initially" * 2, "internal", "observed", "obtained", "occurrences", "of major importance", "operated", "opt for", "option" * 4, "options", "performed" * 6, "posterior", "primary", "procedure" * 4, "produced", "recommended" * 2, "reinforce", "remained" * 4, "remaining", "review" * 2, "similar", "tertiary", "the fact that", "usually" * 2 | "made fit", "close to, near, next to, beside, by", "choices", "review, breakdown, exam, study", "seems, comes", "proper (adj.), set aside (verb)", "and, also", "try", "help", "gristle", "class, group", "mass, heap", "joined", "about, on", "closed, ended", "combination, merger", "having, holding", "make, grow", "made, grown", "rose, lifted up", "equal", "checking, rating", "check", "trade", "show, tell", "outer", "last", "act, role", "risky, unsafe", "but", "named, found", "at once, right away, right now", "to, for", "partial", "clue, sign", "first", "at first", "inner, inside", "saw, seen", "got", "events", "is important, are important, was important", "ran, worked", "choose", "choice, way", "choices, ways", "did/done", "rear", "main, first", "rule, way, method", "made", "suggested", "strengthen", "stayed", "staying, left over", "check", "like", "third", "that", "often" |
| 11 | 54 | "achieved", "adequate", "administered", "alternative", "appropriate", "as long as", "as to whether", "at the time", "containing", "continuing", "determined", "developing" * 2, "difficult" * 2, "difficulty", "evident", "general", "generally", "however" * 2, "initially", "minimise", "minimised", "operated" * 3, "option", "performed" * 3, "performing" * 2, "predominant", "previous" * 3, "prior to" * 5, "recommended", "remain", "require", "required" * 2, "result in", "resulting in", "review", "reviewed", "therefore", "usually", "viable" | "did, mad", "enough", "managed", "choice", "proper (adj.), set aside (verb)", "if, since", "whether", "when", "having, holding", "keeping on", "decided, figured", "making, growing", "hard", "trouble", "clear", "broad", "broadly", "but", "at first", "decrease, lessen", "decreased, lessened", "ran, worked", "choice, way", "did/done", "doing", "superior", "earlier, past", "before", "suggested", "stay", "need", "needed", "lead to", "leading to", "check", "checked", "so, thus", "often", "workable" |
| 12 | 23 | "advantages", "appropriate", "cartilage" * 2, "determined", "external", "frequently", "function", "generally", "however", "in an effort to", "options", "performed" * 2, "portion", "position", "procedure", "reducing", "requires" * 3, "satisfied", "subsequently" | "pluses", "proper (adj.), set aside (verb)", "gristle", "decided, figured", "outer", "often", "act, role", "broadly", "but", "to", "choices, ways", "did/done", "part", "place", "rule, way, method", "cutting", "needs", "happy, content", "later, after" |
| 13 | 52 | "ability", "additional", "an alternative", "approximately", "benefits", "cartilage", "continues", "convenient", "developing", "difficulty", "elevate", "elevating" * 2, "elevation", "except when", "general", "however", "immediately", "impaired", "in the case of", "incision" * 2, "incisions", "modify", "obtained", "operated" * 2, "operating", "perform", "performed", "prior to", "purchased", "recommended", "reduce" * 7, "require" * 2, "required" * 2, "usually" * 7, "write down" | "skill", "added, extra", "any other, another", "about", "helps", "gristle", "keeps on", "handy", "making, growing", "trouble", "raise, lift up", "raising, lifting up", "height", "unless", "broad", "but", "at once, right away, right now", "harmed, weakened, reduced", "in, with, if, by, for (or delete)", "cut", "cuts", "change", "got", "ran, worked", "running, working", "do", "did/done", "before", "bought", "suggested", "cut", "need", "needed", "often", "write" |
| 14 | 43 | "anticipated", "appear", "appropriate", "assistance" * 2, "at all times", "benefit", "continue", "contribute", "develop", "elevated", "ensure", "function" * 3, "however" * 3, "it is important that", "necessary", "operated", "outside of" * 2, "participated", "preparation", "procedure" * 3, "recommended" * 2, "reduce" * 2, "reduces" * 2, "remain", "request", "require" * 2, "required" * 2, "usually" * 3 | "expected, awaited", "seem, come", "proper (adj.), set aside (verb)", "help", "always", "help", "keep, keep on", "give, help", "make, grow", "rose, lifted up", "make sure", "act, role", "but", "must, should", "needed", "ran, worked", "outside (unless proceeding a pronoun)", "took part", "readiness", "rule, way, method", "suggested", "cut", "cuts", "stay", "ask", "need", "needed", "often" |
| 15 | 13 | "ability", "advantages", "consequently", "criteria", "develop", "deviates", "eliminating", "generally", "individuals", "observed", "procedure", "reducing", "usually" | "skill", "pluses", "so", "requirements", "make, grow", "strays, turns away", "cutting, dropping", "broadly", "people", "saw, seen", "rule, way, method", "cutting", "often" |
| 16 | 56 | "ability" * 2, "alleviates", "benefits", "by means of", "cartilage" * 5, "create", "created", "creating", "determine", "determined", "develop" * 2, "generally", "however", "in certain cases", "in conjunction with", "in order to", "incision", "incisions", "known as" * 4, "necessary", "performed" * 3, "portion" * 5, "position" * 2, "procedure" * 5, "procedures" * 4, "reduces", "remain", "result in" * 2, "typically" * 3, "variations" | "skill", "makes easier", "helps", "by, with, from, in, over, through", "gristle", "make", "made", "making", "decide, figure", "decided, figured", "make, grow", "broadly", "but", "at times, sometimes", "along with, and, combined with, coupled with, joined with, paired with, with", "to, for", "cut", "cuts", "called, named", "needed", "did/done", "part", "place", "rule, way, method", "rules, ways, methods", "cuts", "stay", "lead to", "often", "changes, differences" |
| 17 | 397 | "a certain amount of", "a wide range of", "ability", "accompanied" * 2, "accompanying", "accordingly" * 2, "accuracy" * 7, "accurate" * 3, "achievable", "achieved" * 8, "achieving", "acquired" * 3, "adapted" * 3, "additional" * 5, "alternative", "analysis" * 2, "and also", "anterior" * 8, "apex" * 3, "appear", "appears", "appropriate" * 3, "approximately", "are prone to", "as a result of", "ascending", "available" * 5, "beneficial", "bilateral" * 2, "capacity" * 3, "cartilage" * 27, "characterized", "combined" * 7, "compensate", "congenital" * 6, "consolidation" * 2, "consolidations", "construct", "construction", "constructs" * 3, "contemplating", "continue", "continued", "continues", "continuing", "created", "demonstrate", "demonstrated", "desired", "determining", "develop", "developed", "deviation", "difficult", "difficulties", "encouraging", "ensure", "equines", "established", "evaluated" * 2, "evaluating", "evaluation" * 3, "evidences" * 2, "evident", "explaining", "external" * 4, "final" * 2, "frequently", "function", "general consensus", "generally" * 2, "generate", "generating" * 2, "has no", "however" * 6, "identification", "identified" * 2, "identify", "identifying" * 2, "in advance of", "in association with" * 2, "in cases where", "in isolation", "in order to" * 3, "in terms of" * 3, "in the absence of", "in the context of" * 2, "in the sense that", "indicated" * 9, "indicating" * 2, "indication" * 5, "indications" * 7, "initial" * 4, "internal" * 3, "investigated", "is composed of", "known as", "limitation", "limited number of", "located", "location", "maintained", "maximum", "modify" * 2, "multiple" * 5, "necessary", "necessitate" * 2, "necessitates", "observed" * 7, "obtain", "obtained", "occurrence" * 2, "on the other hand", "operated" * 3, "option" * 2, "options", "outside of", "particular", "perform" * 2, "performed" * 18, "performing" * 2, "position" * 4, "positioned", "positioning" * 3, "posterior" * 12, "previous" * 2, "previously", "primary" * 2, "prior to" * 3, "procedure" * 11, "procedures" * 11, "produces", "propagated", "rapid", "recommended" * 2, "reduce" * 3, "reduced", "reduces" * 2, "reduction", "request", "required" * 3, "requiring", "result in" * 2, "resulting in" * 2, "results in", "reversion", "review" * 2, "satisfied" * 4, "similar", "strategy", "subsequent" * 2, "substantial" * 2, "sufficient" * 3, "sufficiently", "taken into consideration", "the majority of", "the presence of", "therefore" * 6, "uncommonly", "usually", "utilized", "value" * 5, "whenever", "whereas" * 2, "with regard to" | "some, much", "assorted, extensive, numerous", "skill", "went with", "going with", "so, just so", "correctness, exactness", "correct, exact", "doable, makeable", "did, mad", "doing, making", "gained, got", "made fit", "added, extra", "choice", "review, breakdown, exam, study", "and, also", "front", "tip", "seem, come", "seems, comes", "proper (adj.), set aside (verb)", "about", "tend to", "because of, due to, following", "climbing, upward", "offered, ready", "helpful", "two-sided", "ability, power, position", "gristle", "described", "joined", "pay", "inborn", "combination, merger", "combinations, mergers", "build", "building", "builds", "thinking about", "keep, keep on", "kept on", "keeps on", "keeping on", "made", "show", "showed", "wished", "deciding, figuring", "make, grow", "made, grown", "change", "hard", "troubles", "urging", "make sure", "horses", "set up, proved", "checked, rated", "checking, rating", "check, rating", "shows", "clear", "showing, telling", "outer", "last", "often", "act, role", "consensus", "broadly", "create, make", "creating, making", "lacks", "but", "ID", "named, found", "name, find", "naming, finding", "ahead of, before, by", "along with, as well as", "where", "along", "to, for", "as for", "without", "in, about, for, of", "in that", "shown", "showing", "clue, sign", "clues, signs", "first", "inner, inside", "reviewed, checked, looked over", "comprises", "called, named", "limit", "a few, little, meager, not many, scant, only so many, some, spare, sparse", "found", "place", "kept, supported", "most, greatest", "change", "many", "needed", "cause, need", "causes, needs", "saw, seen", "get", "got", "event", "however", "ran, worked", "choice, way", "choices, ways", "outside (unless proceeding a pronoun)", "specific", "do", "did/done", "doing", "place", "placed", "placing", "rear", "earlier, past", "before, earlier", "main, first", "before", "rule, way, method", "rules, ways, methods", "makes", "bred, reproduced", "quick", "suggested", "cut", "cut", "cuts", "cut", "ask", "needed", "needing", "lead to", "leading to", "leads to", "return", "check", "happy, content", "like", "plan", "later, next", "real, strong, large", "enough, ample", "amply", "considered", "most, most of", "Consider removing this", "so, thus", "rarely", "often", "used", "cost, worth", "when", "since", "about, regarding" |
| 18 | 18 | "cartilage" * 2, "determines", "incision" * 2, "maintain", "perform", "performed" * 3, "procedure" * 4, "required", "requirement", "the space of", "usually" | "gristle", "decides, figures", "cut", "keep, support", "do", "did/done", "rule, way, method", "needed", "need", "Consider removing this", "often" |
| 19 | 189 | "accelerate", "accordingly", "accurate" * 4, "accurately" * 2, "achieve", "achieved" * 2, "achieves", "adapted", "advantage", "advantages" * 3, "and also", "anterior" * 9, "appropriate" * 2, "approximately" * 3, "as well as", "attempted", "available", "beneficial", "benefit", "bilateral" * 3, "cartilage" * 4, "combined" * 3, "compensate", "component", "contains", "contribute", "contribution", "convenient", "conversion", "demonstrate", "desired", "difficult" * 3, "examination", "examine", "facilitate", "feasible", "general" * 7, "generally", "however" * 13, "identification", "imminent", "in conjunction with", "in contrast to", "in most cases", "in order to", "incision" * 3, "indicated" * 4, "indication", "indications" * 3, "individuals", "individuals who", "inferior" * 4, "initially", "location", "maintain" * 2, "maintained", "maintains" * 2, "minimize", "minimized", "necessitate", "obtain", "obtained", "obtains", "on the surface", "option", "perform", "performed" * 6, "performing", "position" * 3, "positioned", "posterior" * 23, "primary" * 2, "procedure" * 2, "reason why", "recommended" * 3, "recommends", "reduce" * 2, "reduces" * 2, "remaining", "require" * 4, "required" * 2, "requires" * 2, "retain", "similar" * 2, "subsequent", "subsequently", "sufficient", "sufficiently" * 2, "therefore" * 2 | "hasten, quicken", "so, just so", "correct, exact", "correctly, exactly", "do, make", "did, mad", "does, makes", "made fit", "plus", "pluses", "and, also", "front", "proper (adj.), set aside (verb)", "about", "and, also", "tried", "offered, ready", "helpful", "help", "two-sided", "gristle", "joined", "pay", "part", "has, holds", "give, help", "gift", "handy", "change", "show", "wished", "hard", "check", "check, look at", "ease, help", "can be done", "broad", "broadly", "but", "ID", "near", "along with, and, combined with, coupled with, joined with, paired with, with", "compared to", "mostly, most of these, often, usually", "to, for", "cut", "shown", "clue, sign", "clues, signs", "people", "those who", "lesser", "at first", "place", "keep, support", "kept, supported", "keeps, supports", "decrease, lessen", "decreased, lessened", "cause, need", "get", "got", "gets", "seemingly, apparently", "choice, way", "do", "did/done", "doing", "place", "placed", "rear", "main, first", "rule, way, method", "reason", "suggested", "suggests", "cut", "cuts", "staying, left over", "need", "needed", "needs", "keep, hold", "like", "later, next", "later, after", "enough, ample", "amply", "so, thus" |
| 20 | 50 | "achieve", "achieved", "adapted", "adequate", "alternative", "appropriate", "assisted" * 4, "at all times", "cartilage", "continue" * 8, "continue on", "determine", "eccentric" * 2, "elevation", "encourage" * 2, "ensure", "function", "indications", "individual", "individuals", "initiate", "limitations", "maintain", "maintaining", "monitor", "numerous", "performing", "position", "possess", "previous", "procedure", "reduce", "remaining", "required", "transmits", "transmitted", "typically", "variations" | "do, make", "did, mad", "made fit", "enough", "choice", "proper (adj.), set aside (verb)", "aided, helped", "always", "gristle", "keep, keep on", "continue", "decide, figure", "odd", "height", "urge", "make sure", "act, role", "clues, signs", "person, single", "people", "begin", "limits", "keep, support", "keeping, supporting", "check, watch", "many", "doing", "place", "have, own", "earlier, past", "rule, way, method", "cut", "staying, left over", "needed", "sends", "sent", "often", "changes, differences" |
| 21 | 108 | "ability", "accurate", "anterior" * 5, "as well as", "bilateral" * 2, "cartilage" * 10, "category", "certainly", "combined" * 2, "encouraging", "ensure", "evaluation" * 2, "evident", "final", "generally" * 2, "identification", "identifies", "identifying", "in association with" * 4, "in cases where", "in order to" * 2, "indicated" * 5, "indicating", "indication" * 2, "indications" * 4, "initial" * 3, "initially" * 2, "is dependent on", "limited number of", "modify", "necessary", "observed" * 2, "obtain", "on the contrary", "on the other hand", "particular" * 2, "perform", "performed" * 8, "position", "positioned", "positioning" * 2, "posterior" * 8, "previous", "procedure" * 3, "procedures" * 3, "reduces", "resulted in", "resulting in", "sooner or later", "sufficient", "the fact that", "the majority of", "the presence of", "value", "with regard to" | "skill", "correct, exact", "front", "and, also", "two-sided", "gristle", "class, group", "surely", "joined", "urging", "make sure", "check, rating", "clear", "last", "broadly", "ID", "names, finds", "naming, finding", "along with, as well as", "where", "to, for", "shown", "showing", "clue, sign", "clues, signs", "first", "at first", "depends on, hinges on", "a few, little, meager, not many, scant, only so many, some, spare, sparse", "change", "needed", "saw, seen", "get", "rather, instead", "however", "specific", "do", "did/done", "place", "placed", "placing", "rear", "earlier, past", "rule, way, method", "rules, ways, methods", "cuts", "lead to", "leading to", "eventually", "enough, ample", "that", "most, most of", "Consider removing this", "cost, worth", "about, regarding" |
| 22 | 56 | "appropriate", "as well as", "cartilage" * 3, "determines", "difficult", "elevated" * 2, "evaluate", "general", "incision" * 4, "incisions", "indicated", "maintain", "multiple", "obtained", "operated", "perform", "performed" * 7, "portion", "portions" * 3, "position", "primary", "procedure" * 8, "procedures", "reciprocating", "reduces", "required", "requirement", "segment" * 4, "so as to", "the space of", "usually", "visualize" | "proper (adj.), set aside (verb)", "and, also", "gristle", "decides, figures", "hard", "rose, lifted up", "check, rate", "broad", "cut", "cuts", "shown", "keep, support", "many", "got", "ran, worked", "do", "did/done", "part", "parts", "place", "main, first", "rule, way, method", "rules, ways, methods", "giving in return", "cuts", "needed", "need", "part", "to", "Consider removing this", "often", "picture" |
| 23 | 53 | "additional", "adjustment", "at the time" * 2, "component" * 2, "components" * 2, "conclude", "concluded", "conversion" * 6, "demonstrated", "detrimental", "difficult", "frequently", "function", "however", "inferior", "minimize" * 2, "performed" * 2, "position" * 2, "posterior", "previous" * 3, "primary" * 4, "procedure", "procedures", "produced", "reducing", "require" * 2, "required" * 3, "requiring", "review", "similar" * 2, "subsequent", "substantially", "the presence of" | "added, extra", "settlement", "when", "part", "parts", "close, end", "closed, ended", "change", "showed", "harmful", "hard", "often", "act, role", "but", "lesser", "decrease, lessen", "did/done", "place", "rear", "earlier, past", "main, first", "rule, way, method", "rules, ways, methods", "made", "cutting", "need", "needed", "needing", "check", "like", "later, next", "really, strongly, largely", "Consider removing this" |
| 24 | 173 | "a great deal of", "accuracy", "accurate" * 2, "accurately", "achieved" * 3, "actually" * 2, "additional", "adjustment", "adjustments", "advantages", "alterations" * 2, "alternative" * 3, "anterior" * 2, "apex", "appear", "appeared", "appears", "approximately" * 2, "as a result", "as to whether" * 2, "as well as", "ascending", "available" * 2, "benefit", "bilateral", "cartilage" * 4, "combined" * 2, "combining", "concluded" * 2, "conclusion", "consolidation", "contributing", "creates", "definitive" * 2, "demonstrated" * 3, "desired", "determine", "determined" * 2, "developed", "difficulties", "eminence", "evidenced", "examination" * 2, "external" * 3, "function" * 3, "general" * 2, "generally", "however" * 9, "in most cases", "in order to" * 4, "in spite of", "incomplete", "indication" * 5, "indications", "insufficient" * 2, "internal", "investigated" * 2, "modifications" * 2, "modified", "multiple", "necessary", "observed" * 2, "on the basis of", "on the contrary", "particular", "performed", "position", "posterior" * 4, "previous", "primary", "procedure" * 14, "procedures", "propensity" * 2, "rapid", "recommend" * 2, "reduce" * 2, "reduced", "reducing" * 4, "reduction", "remains", "require", "required", "requiring", "result in" * 2, "resulting in", "results in", "review" * 2, "solicited", "strategy" * 2, "subsequently", "the majority of", "the presence of" * 2, "therefore", "transmitted", "typically", "usually" * 6, "whereas" | "much, vast", "correctness, exactness", "correct, exact", "correctly, exactly", "did, mad", "really", "added, extra", "settlement", "settlements", "pluses", "changes", "choice", "front", "tip", "seem, come", "seemed, came", "seems, comes", "about", "so, then, thus", "whether", "and, also", "climbing, upward", "offered, ready", "help", "two-sided", "gristle", "joined", "joining", "closed, ended", "close, end", "combination, merger", "giving, helping", "makes", "final", "showed", "wished", "decide, figure", "decided, figured", "made, grown", "troubles", "high place", "showed", "check", "outer", "act, role", "broad", "broadly", "but", "mostly, most of these, often, usually", "to, for", "aside, despite, although", "partial", "clue, sign", "clues, signs", "not enough", "inner, inside", "reviewed, checked, looked over", "changes", "changed", "many", "needed", "saw, seen", "by, from, because of, assuming, based on, from", "rather, instead", "specific", "did/done", "place", "rear", "earlier, past", "main, first", "rule, way, method", "rules, ways, methods", "inclination, tendency", "quick", "suggest", "cut", "cut", "cutting", "cut", "stays", "need", "needed", "needing", "lead to", "leading to", "leads to", "check", "asked for", "plan", "later, after", "most, most of", "Consider removing this", "so, thus", "sent", "often", "often", "since" |
| 25 | 15 | "administered", "cartilage" * 3, "general", "indicated", "outside of" * 2, "performed" * 2, "procedure" * 2, "reduced", "usually" * 2 | "managed", "gristle", "broad", "shown", "outside (unless proceeding a pronoun)", "did/done", "rule, way, method", "cut", "often" |
| 26 | 45 | "accelerated", "accuracy" * 4, "accurately", "achieve", "actual", "appropriate", "concerning", "contains", "create", "desired", "difficult", "difficulties", "exchanged", "exchanging", "generate", "horizontally", "however" * 2, "identical", "incision", "indicate" * 2, "initial", "oblique", "obtaining", "perform", "performing" * 2, "positioning", "prior to", "procedure" * 2, "procedures" * 2, "reduce", "remains" * 3, "subsequently", "sufficient", "therefore", "value" | "hastened, quickened", "correctness, exactness", "correctly, exactly", "do, make", "real", "proper (adj.), set aside (verb)", "about, on", "has, holds", "make", "wished", "hard", "troubles", "traded", "trading", "create, make", "sideways", "but", "same", "cut", "show", "first", "slanting", "getting", "do", "doing", "placing", "before", "rule, way, method", "rules, ways, methods", "cut", "stays", "later, after", "enough, ample", "so, thus", "cost, worth" |
| 27 | 42 | "alleviating", "an alternative", "as opposed to", "as well as", "benefit", "benefits", "cartilage", "develop", "developed", "function" * 3, "general", "have the ability to" * 2, "immediately" * 2, "impair", "impaired" * 4, "in order to", "necessary", "option", "performed" * 3, "portion", "position" * 2, "procedure" * 4, "reimburse", "require", "result in", "results in", "submit", "usually" * 2 | "making easier", "any other, another", "compared to", "and, also", "help", "helps", "gristle", "make, grow", "made, grown", "act, role", "broad", "can", "at once, right away, right now", "harm, weaken, reduce", "harmed, weakened, reduced", "to, for", "needed", "choice, way", "did/done", "part", "place", "rule, way, method", "pay back", "need", "lead to", "leads to", "send, give", "often" |
| 28 | 38 | "accurate" * 2, "available", "beneficial", "benefits", "characteristics", "commitment", "difficult", "ensure", "generally", "indicated" * 2, "is able to", "location", "necessary", "option", "perform", "preparation" * 2, "procedure" * 6, "procedures", "recommended", "reduce" * 4, "regimen", "required" * 2, "take into consideration" * 2, "typically", "validate" | "correct, exact", "offered, ready", "helpful", "helps", "traits", "pledge", "hard", "make sure", "broadly", "shown", "can", "place", "needed", "choice, way", "do", "readiness", "rule, way, method", "rules, ways, methods", "suggested", "cut", "routine, rule", "needed", "consider", "often", "confirm" |
| 29 | 10 | "create", "function", "in most cases", "incision", "maintain", "monitored" * 2, "operated", "typically", "usually" | "make", "act, role", "mostly, most of these, often, usually", "cut", "keep, support", "checked, watched", "ran, worked", "often", "often" |
| 30 | 32 | "and also", "assist", "beneficial", "cartilage" * 3, "contribute", "determines" * 2, "developing", "encouraged", "immediately" * 2, "incision" * 2, "maintain", "operated" * 2, "performing", "previous", "procedure" * 6, "recommended", "reduce" * 2, "require", "requirement", "usually" | "and, also", "aid, help", "helpful", "gristle", "give, help", "decides, figures", "making, growing", "urged", "at once, right away, right now", "cut", "keep, support", "ran, worked", "doing", "earlier, past", "rule, way, method", "suggested", "cut", "need", "need", "often" |
| 31 | 136 | "ability" * 2, "abundant", "accelerated", "achieve", "achieved" * 2, "actual", "additional" * 3, "adequate", "advantage", "advantages", "aggressive", "alternative", "approval", "as to whether", "as well as" * 8, "available" * 3, "bilateral" * 6, "cartilage" * 3, "conclusion", "continue", "contribute", "determined" * 2, "developing" * 2, "discovered" * 2, "evaluation" * 2, "function" * 3, "impaired" * 4, "imperative", "in order to", "in terms of" * 3, "incision" * 2, "initial", "internal" * 3, "investigate" * 5, "known as" * 2, "limitations", "location" * 2, "maintain", "obtain" * 2, "obtained", "operated", "operating", "outside of", "participate", "perform" * 6, "performed" * 6, "performing", "position", "predominantly" * 2, "prior to", "procedure" * 10, "procedures" * 2, "program" * 3, "rapid", "reduced", "require", "resulting in", "review" * 4, "sufficiently", "typically" * 4, "usually", "utilize" * 2, "utilized", "visualize", "with regard to" | "skill", "enough", "hastened, quickened", "do, make", "did, mad", "real", "added, extra", "enough", "plus", "pluses", "forward, strong, attacking", "choice", "praise, consent", "whether", "and, also", "offered, ready", "two-sided", "gristle", "close, end", "keep, keep on", "give, help", "decided, figured", "making, growing", "found out", "check, rating", "act, role", "harmed, weakened, reduced", "urgent", "to, for", "as for", "cut", "first", "inner, inside", "review, check, look over", "called, named", "limits", "place", "keep, support", "get", "got", "ran, worked", "running, working", "outside (unless proceeding a pronoun)", "take part", "do", "did/done", "doing", "place", "superiorly", "before", "rule, way, method", "rules, ways, methods", "plan", "quick", "cut", "need", "leading to", "check", "amply", "often", "often", "use", "used", "picture", "about, regarding" |
| 32 | 116 | "acceptable", "additional", "adjustment", "adjustments", "alternative", "and also", "available" * 2, "beneficial", "benefit", "bilateral" * 2, "category", "combined", "constitute", "constituted", "contains", "contributed", "conversion" * 4, "conversions" * 2, "criteria", "ensures", "external" * 2, "frequently", "have no", "however" * 5, "identification", "identified" * 6, "identify" * 3, "in contrast to", "in order to", "in spite of", "incomplete", "indicates", "indication", "internal" * 2, "investigated", "is consistent with", "it is probable that", "majority" * 2, "monitor", "not possible", "observed", "operated" * 3, "operating" * 3, "option", "options", "performed" * 12, "performing" * 3, "previous", "primary" * 5, "procedure" * 4, "procedures" * 8, "produced", "reduced", "results in", "reviewing", "satisfied", "similar" * 2, "speculate", "the fact that", "the majority of" * 3, "the reason for", "thereof" | "welcome", "added, extra", "settlement", "settlements", "choice", "and, also", "offered, ready", "helpful", "help", "two-sided", "class, group", "joined", "be, form", "was, formed", "has, holds", "gave, helped", "change", "changes", "requirements", "makes sure", "outer", "often", "lacks", "but", "ID", "named, found", "name, find", "compared to", "to, for", "aside, despite, although", "partial", "shows", "clue, sign", "inner, inside", "reviewed, checked, looked over", "coheres to, conforms with", "probably", "most", "check, watch", "impossible", "saw, seen", "ran, worked", "running, working", "choice, way", "choices, ways", "did/done", "doing", "earlier, past", "main, first", "rule, way, method", "rules, ways, methods", "made", "cut", "leads to", "checking", "happy, content", "like", "reflect, guess, surmise, suppose", "that", "most, most of", "because, since, why", "its, their" |
| 33 | 49 | "achieve", "adequate", "advantage", "alleviated", "as opposed to", "as well as", "available", "cartilage" * 3, "continues", "develop", "function" * 2, "however", "in order to", "incision", "incisions", "monitored", "operating", "options", "perform", "performed", "prior to", "procedure" * 13, "reduce", "reducing", "remain", "required", "satisfied", "segment" * 2, "subsequent", "the majority of" * 2, "therefore", "usually" | "do, make", "enough", "plus", "made easier", "compared to", "and, also", "offered, ready", "gristle", "keeps on", "make, grow", "act, role", "but", "to, for", "cut", "cuts", "checked, watched", "running, working", "choices, ways", "do", "did/done", "before", "rule, way, method", "cut", "cutting", "stay", "needed", "happy, content", "part", "later, next", "most, most of", "so, thus", "often" |
| 34 | 124 | "accordingly", "achieve", "achieved" * 2, "advantage", "advantages" * 2, "advised", "alteration", "amendments", "analysis", "anterior" * 2, "approval", "as of now", "assisted", "bilateral" * 2, "combined" * 2, "concluded" * 2, "consolidation", "constitutes" * 2, "contributing", "conversion", "criteria" * 2, "demonstrated" * 3, "density", "desired", "developed" * 6, "difficult", "ensured", "evaluate", "evaluation" * 2, "evaluations", "examination" * 2, "final" * 3, "general", "generally", "has no", "however" * 4, "in contrast to", "in spite of", "incision", "initiated", "inquiries", "internal", "limitations", "maintain", "maintained" * 2, "modification" * 2, "modified", "numerous" * 2, "oblique" * 4, "observed", "obtained", "operated", "operating", "participating", "particular", "performed" * 3, "posterior" * 10, "previous" * 2, "primary", "probability", "procedure" * 7, "procedures", "rapid", "rapidly", "reduce", "remaining", "required" * 2, "requiring", "rigidity" * 2, "segment", "similar", "subsequent", "superior", "utilization", "utilized", "utilizing" | "so, just so", "do, make", "did, mad", "plus", "pluses", "told, recommended", "change", "changes", "review, breakdown, exam, study", "front", "praise, consent", "about", "aided, helped", "two-sided", "joined", "closed, ended", "combination, merger", "makes up, forms", "giving, helping", "change", "requirements", "showed", "thickness", "wished", "made, grown", "hard", "made sure", "check, rate", "check, rating", "checks, ratings", "check", "last", "broad", "broadly", "lacks", "but", "compared to", "aside, despite, although", "cut", "started", "questions", "inner, inside", "limits", "keep, support", "kept, supported", "change", "changed", "many", "slanting", "saw, seen", "got", "ran, worked", "running, working", "taking part", "specific", "did/done", "rear", "earlier, past", "main, first", "chance", "rule, way, method", "rules, ways, methods", "quick", "quickly", "cut", "staying, left over", "needed", "needing", "stiffness", "part", "like", "later, next", "better, boss", "use", "used", "using" |
| 35 | 147 | "accompanied", "achieve", "achieving", "additional", "adequate", "adjacent", "analysis" * 3, "as a result", "as well as" * 2, "at the time" * 3, "cartilage" * 3, "categories", "category", "certainly", "clarify", "combined", "concluded" * 3, "consequently", "consolidation", "continue", "conversion", "criteria", "demonstrated" * 4, "demonstrates" * 2, "desired", "despite the fact that", "determine", "develop", "developing", "deviation", "due to the fact that", "elevated", "evaluate", "evaluated" * 6, "evaluation", "external", "final", "function" * 2, "fundamental", "general" * 3, "generally", "heterogeneous", "high degree of", "however" * 3, "identified", "implementation", "indications" * 4, "initial", "is able to", "it should be noted", "limitation", "massive", "methodology", "monitoring", "multiple", "necessary", "not many", "not possible", "objective" * 9, "objectives", "observed" * 4, "obtained" * 2, "on the side of", "operated" * 5, "particular" * 2, "performed" * 11, "posterior", "predominant", "preferable", "previous", "primary" * 3, "program" * 2, "recommend", "reduce", "reducing", "reduction", "remains", "results in" * 3, "review", "similar" * 2, "subsequent", "sufficient", "the date of", "the sum of", "therefore" * 2, "value", "visualized" | "went with", "do, make", "doing, making", "added, extra", "enough", "next to", "review, breakdown, exam, study", "so, then, thus", "and, also", "when", "gristle", "classes, groups", "class, group", "surely", "make clear", "joined", "closed, ended", "so", "combination, merger", "keep, keep on", "change", "requirements", "showed", "shows", "wished", "although, even though, despite", "decide, figure", "make, grow", "making, growing", "change", "because, since, given that", "rose, lifted up", "check, rate", "checked, rated", "check, rating", "outer", "last", "act, role", "basic", "broad", "broadly", "varied", "abundant, ample", "but", "named, found", "carrying out", "clues, signs", "first", "can", "note", "limit", "large", "method, design, plan", "checking, watching", "many", "needed", "few", "impossible", "aim, goal", "aims, goals", "saw, seen", "got", "with", "ran, worked", "specific", "did/done", "rear", "superior", "better", "earlier, past", "main, first", "plan", "suggest", "cut", "cutting", "cut", "stays", "leads to", "check", "like", "later, next", "enough, ample", "Consider removing this", "all", "so, thus", "cost, worth", "pictured" |
| 36 | 48 | "accuracy", "achieve", "achieved", "an alternative", "appropriate", "at about", "benefits", "cartilage" * 3, "desired" * 2, "develop", "difficult" * 2, "ensure", "external" * 4, "function", "general", "generally" * 3, "however", "in some cases", "initial", "necessary", "operating", "perform", "performed" * 6, "prior to", "procedure" * 5, "reduced", "requiring", "similar" * 2, "usually" | "correctness, exactness", "do, make", "did, mad", "any other, another", "proper (adj.), set aside (verb)", "about", "helps", "gristle", "wished", "make, grow", "hard", "make sure", "outer", "act, role", "broad", "broadly", "but", "at times, sometimes", "first", "needed", "running, working", "do", "did/done", "before", "rule, way, method", "cut", "needing", "like", "often" |
| 37 | 28 | "assistance", "benefits" * 2, "elevated", "general" * 3, "however", "incision", "necessary", "outside of", "performed" * 3, "procedure" * 4, "procedures", "reduce", "remain" * 2, "remaining", "required", "result in", "usually" * 3 | "help", "helps", "rose, lifted up", "broad", "but", "cut", "needed", "outside (unless proceeding a pronoun)", "did/done", "rule, way, method", "rules, ways, methods", "cut", "stay", "staying, left over", "needed", "lead to", "often" |
| 38 | 16 | "assist", "cartilage" * 2, "continue" * 2, "elevating", "general", "incision", "operated", "outside of", "performed" * 3, "procedure", "usually" * 2 | "aid, help", "gristle", "keep, keep on", "raising, lifting up", "broad", "cut", "ran, worked", "outside (unless proceeding a pronoun)", "did/done", "rule, way, method", "often" |
| 39 | 442 | "a number of", "ability", "absolutely", "absolutely essential", "accordingly", "accurate" * 6, "accurately" * 3, "achieve" * 4, "achieved" * 3, "achieving", "actually" * 23, "additional", "advantages" * 2, "advise", "advised", "all of" * 2, "alleviate", "almost all", "alternative" * 4, "and also", "anterior", "appropriate" * 4, "appropriately", "approximately" * 5, "as a result of", "as long as" * 2, "as opposed to", "as well as" * 5, "assuming that", "at present", "at the time" * 5, "at this time", "attempts", "attractive", "available" * 3, "benefit" * 3, "both of", "cartilage" * 17, "certainly" * 2, "combined" * 2, "commences", "compress" * 2, "compresses", "construct", "contemplate", "continues", "create" * 2, "created", "creates" * 2, "creating" * 2, "desired", "determine", "detrimental", "develop" * 2, "developed" * 8, "developing", "develops" * 2, "difficult" * 6, "difficulty", "discovered", "disrupt", "disrupted", "disrupting", "eccentric" * 2, "eliminated", "encourages", "explain", "explaining", "external" * 2, "familiar", "for the sake of", "frequently" * 4, "from start to finish", "function" * 2, "general" * 3, "generally" * 3, "has a tendency", "has no", "however" * 2, "identify", "immediately" * 4, "impact" * 4, "in association with", "in contrast to", "in most cases", "in order for", "in order to", "in other words", "in some cases", "in terms of" * 6, "in the case of" * 3, "in the right", "inasmuch as", "incision" * 2, "inconvenience", "indicating", "indication" * 2, "indications", "individual", "individuals", "initially" * 4, "insufficient", "internal", "investigated", "just about", "known as" * 2, "limitation", "maintain" * 2, "maintained", "manufactured", "manufactures", "meticulous", "modification", "monitor" * 2, "monitoring", "necessary", "need to have" * 4, "needs to have", "never ever", "no more than", "oblique", "on the other hand", "operated" * 2, "operating" * 4, "option" * 7, "options" * 7, "outside of" * 3, "over the course of", "particular" * 2, "perform" * 3, "performed" * 8, "performing" * 4, "permit", "point of view", "portion", "position" * 22, "posterior" * 2, "previous" * 2, "previously" * 2, "prior to" * 4, "procedure" * 27, "procedures" * 7, "producing", "reason why", "reduce" * 8, "reduced" * 4, "reduces", "reducing", "reduction" * 2, "regimen", "remain" * 2, "remains" * 2, "require" * 5, "required" * 8, "requirements", "requiring" * 2, "retention", "reviewed", "sufficient", "superior" * 2, "taken into account", "the concept of", "the fact that", "the other way around", "therefore" * 5, "up until", "usually" * 20, "via" * 3, "whereas" * 2, "with regard to" | "a few", "skill", "wholly", "essential", "so, just so", "correct, exact", "correctly, exactly", "do, make", "did, mad", "doing, making", "really", "added, extra", "pluses", "tell, recommend", "told, recommended", "all (unless proceeding a pronoun), Consider removing this", "make easier", "most", "choice", "and, also", "front", "proper (adj.), set aside (verb)", "properly", "about", "because of, due to, following", "if, since", "compared to", "and, also", "if", "now, today", "when", "now, right now", "tries", "pleasing", "offered, ready", "help", "both (unless proceeding a pronoun)", "gristle", "surely", "joined", "begins", "sqeeze", "sqeezes", "build", "think about", "keeps on", "make", "made", "makes", "making", "wished", "decide, figure", "harmful", "make, grow", "made, grown", "making, growing", "makes, grows", "hard", "trouble", "found out", "interrupt, confuse", "interrupted, confused", "interrupting, confusing", "odd", "cut, dropped", "urges", "show, tell", "showing, telling", "outer", "known", "for", "often", "completely, thoroughly", "act, role", "broad", "broadly", "tends to", "lacks", "but", "name, find", "at once, right away, right now", "hit, change", "along with, as well as", "compared to", "mostly, most of these, often, usually", "for", "to, for", "that is", "at times, sometimes", "as for", "in, with, if, by, for (or delete)", "correct, right, justified", "because, since, as, as far as", "cut", "bother", "showing", "clue, sign", "clues, signs", "person, single", "people", "at first", "not enough", "inner, inside", "reviewed, checked, looked over", "about", "called, named", "limit", "keep, support", "kept, supported", "made", "makes", "very careful", "change", "check, watch", "checking, watching", "needed", "need", "needs", "never", "only", "slanting", "however", "ran, worked", "running, working", "choice, way", "choices, ways", "outside (unless proceeding a pronoun)", "during, throughout", "specific", "do", "did/done", "doing", "let", "opinion", "part", "place", "rear", "earlier, past", "before, earlier", "before", "rule, way, method", "rules, ways, methods", "making", "reason", "cut", "cut", "cuts", "cutting", "cut", "routine, rule", "stay", "stays", "need", "needed", "needs", "needing", "keeping, holding", "checked", "enough, ample", "better, boss", "considered", "Consider removing this", "that", "the opposite", "so, thus", "until", "often", "in, on, by", "since", "about, regarding" |
| 40 | 75 | "accurately", "alternate", "alternative", "anterior", "at the time", "available", "benefit", "both of", "cartilage" * 8, "combined", "continue" * 3, "develop", "difficulty", "elevation", "examination", "examine", "frequently", "however", "identify", "in association with", "in order to", "in some cases", "in the case of", "incisions", "initiate", "known as" * 5, "minimise", "necessary", "obtain", "operated", "options", "other similar", "perform" * 4, "performed" * 5, "performing", "portion", "predominantly", "procedure" * 2, "procedures", "profound", "reduce" * 2, "request", "require", "similar", "to a certain extent", "up until", "usually" * 7 | "correctly, exactly", "take turns (between), every other (adj.)", "choice", "front", "when", "offered, ready", "help", "both (unless proceeding a pronoun)", "gristle", "joined", "keep, keep on", "make, grow", "trouble", "height", "check", "check, look at", "often", "but", "name, find", "along with, as well as", "to, for", "at times, sometimes", "in, with, if, by, for (or delete)", "cuts", "begin", "called, named", "decrease, lessen", "needed", "get", "ran, worked", "choices, ways", "similar", "do", "did/done", "doing", "part", "superiorly", "rule, way, method", "rules, ways, methods", "deep, thoughtful", "cut", "ask", "need", "like", "in a sense, somewhat, partly", "until", "often" |
| 41 | 25 | "approximately" * 2, "determine" * 2, "difficult", "eliminate", "ensure", "general", "generally", "in order to", "incision", "located", "performed" * 2, "procedure" * 4, "recommend" * 3, "reduce" * 2, "required", "typically" | "about", "decide, figure", "hard", "cut, drop", "make sure", "broad", "broadly", "to, for", "cut", "found", "did/done", "rule, way, method", "suggest", "cut", "needed", "often" |
| 42 | 199 | "a great deal of", "accelerated", "acceptable", "accomplished", "achieved", "additional", "additionally", "adequate", "adjustment", "administered", "advantage", "advantageous", "advantages" * 2, "advised", "alteration", "alternative" * 2, "analysis" * 4, "anterior" * 9, "apparent", "appears", "appropriate", "appropriately", "approximately" * 3, "as well as" * 2, "bilateral", "both of", "cartilage" * 4, "cessation", "concluded", "consolidation", "construction", "continue", "contribute", "created", "creates", "debilitating", "desired" * 2, "determine", "determined" * 3, "determining", "elevated" * 2, "elevates", "elicit" * 2, "endeavors", "ensure" * 4, "ensured", "evidenced" * 2, "examination", "examined", "explain", "external" * 2, "facilitates", "final" * 2, "general", "has a tendency", "has the ability to", "however" * 2, "identified", "identifying", "imperative", "in close proximity to", "in conjunction with" * 2, "in the absence of", "in the case of" * 2, "incision", "indicated", "inhibition", "initially" * 2, "is defined as", "it is important to note", "known as", "maintain" * 2, "maintained" * 2, "maintaining", "modifications" * 2, "modified", "modifying", "multiple", "necessary", "objective", "obtained", "operating", "options" * 3, "perform" * 4, "performed" * 6, "position" * 2, "positioned", "positioning" * 5, "posterior" * 15, "preparation", "primarily", "prior to", "procedure" * 6, "reduce", "reducing" * 2, "remain", "required" * 3, "requiring", "result in", "resulting in", "results in" * 2, "review", "reviewed" * 2, "satisfied" * 3, "scrutinized", "subsequent" * 3, "suitability" * 2, "superior" * 2, "the presence of", "therefore", "typically" * 2, "usually", "via", "viable", "whereas" * 2 | "much, vast", "hastened, quickened", "welcome", "did, done", "did, mad", "added, extra", "added, more", "enough", "settlement", "managed", "plus", "helpful", "pluses", "told, recommended", "change", "choice", "review, breakdown, exam, study", "front", "clear, plain", "seems, comes", "proper (adj.), set aside (verb)", "properly", "about", "and, also", "two-sided", "both (unless proceeding a pronoun)", "gristle", "stop, pause", "closed, ended", "combination, merger", "building", "keep, keep on", "give, help", "made", "makes", "weakening", "wished", "decide, figure", "decided, figured", "deciding, figuring", "rose, lifted up", "raises, lifts up", "draw out, call forth", "tries, attempts", "make sure", "made sure", "showed", "check", "checked, looked at", "show, tell", "outer", "eases, helps", "last", "broad", "tends to", "can", "but", "named, found", "naming, finding", "urgent", "close to, near", "along with, and, combined with, coupled with, joined with, paired with, with", "without", "in, with, if, by, for (or delete)", "cut", "shown", "restraint", "at first", "is", "note", "called, named", "keep, support", "kept, supported", "keeping, supporting", "changes", "changed", "changing", "many", "needed", "aim, goal", "got", "running, working", "choices, ways", "do", "did/done", "place", "placed", "placing", "rear", "readiness", "mainly, firstly", "before", "rule, way, method", "cut", "cutting", "stay", "needed", "needing", "lead to", "leading to", "leads to", "check", "checked", "happy, content", "inspected, examined", "later, next", "fitness", "better, boss", "Consider removing this", "so, thus", "often", "often", "in, on, by", "workable", "since" |
| 43 | 52 | "additional", "adhere", "advise", "advised" * 2, "alterations", "appropriate", "approximately", "as long as", "assistance", "cartilage", "continue", "desired", "developed", "elevated", "explain", "explained", "however" * 2, "individual" * 2, "initial", "initially", "it is important that", "known as", "minimise", "necessary", "no matter how", "notify", "operated" * 3, "portion", "position" * 4, "predominantly", "procedure" * 3, "reduce" * 3, "required", "requires" * 2, "therefore" * 2, "typically", "usually", "variation" | "added, extra", "stick to, follow", "tell, recommend", "told, recommended", "changes", "proper (adj.), set aside (verb)", "about", "if, since", "help", "gristle", "keep, keep on", "wished", "made, grown", "rose, lifted up", "show, tell", "showed, told", "but", "person, single", "first", "at first", "must, should", "called, named", "decrease, lessen", "needed", "however", "let know, tell", "ran, worked", "part", "place", "superiorly", "rule, way, method", "cut", "needed", "needs", "so, thus", "often", "often", "change, difference" |
| 44 | 39 | "a number of", "ability", "appropriate" * 2, "approximately" * 2, "at the time", "at this time", "available" * 2, "benefit" * 2, "elevated", "encourage", "encouraged", "explains", "general", "however", "identified" * 2, "impair", "in order to", "maximum", "necessary", "option", "options" * 2, "outside of", "particular", "prior to", "procedure" * 2, "procedures", "proceed", "reduce" * 2, "require", "result in" * 2 | "a few", "skill", "proper (adj.), set aside (verb)", "about", "when", "now, right now", "offered, ready", "help", "rose, lifted up", "urge", "urged", "shows, tells", "broad", "but", "named, found", "harm, weaken, reduce", "to, for", "most, greatest", "needed", "choice, way", "choices, ways", "outside (unless proceeding a pronoun)", "specific", "before", "rule, way, method", "rules, ways, methods", "do, go on", "cut", "need", "lead to" |
| 45 | 110 | "ability" * 3, "accelerate", "achieve" * 3, "achieved" * 2, "achieving", "adapted", "additional" * 3, "advantage", "advantages" * 2, "adverse" * 2, "anterior" * 5, "apex", "appropriate", "at about", "cartilage", "combined", "comprised", "concluded", "consolidation", "continues", "conversion" * 4, "created" * 2, "desired", "determine" * 3, "determined", "developed", "difficult", "elevated", "evaluated" * 3, "evidences", "examination", "familiar", "final", "generally", "however", "immediately", "in terms of" * 2, "in the presence of", "incision" * 3, "incorporated", "indicated", "indicates", "indications" * 2, "locates", "locating", "location", "maintained", "maximum", "necessary", "necessitates", "observed", "option" * 2, "posterior" * 3, "primary", "procedure", "procedures", "recommended" * 2, "reducing", "remaining", "require", "required" * 5, "requiring", "resulted in", "results in", "rigidity", "segment", "segments", "subsequently", "superior" * 3, "the presence of", "usually", "viable", "whereas" * 3 | "skill", "hasten, quicken", "do, make", "did, mad", "doing, making", "made fit", "added, extra", "plus", "pluses", "harmful", "front", "tip", "proper (adj.), set aside (verb)", "about", "gristle", "joined", "formed, included", "closed, ended", "combination, merger", "keeps on", "change", "made", "wished", "decide, figure", "decided, figured", "made, grown", "hard", "rose, lifted up", "checked, rated", "shows", "check", "known", "last", "broadly", "but", "at once, right away, right now", "as for", "with, before", "cut", "blended, joined, mixed", "shown", "shows", "clues, signs", "finds", "finding", "place", "kept, supported", "most, greatest", "needed", "causes, needs", "saw, seen", "choice, way", "rear", "main, first", "rule, way, method", "rules, ways, methods", "suggested", "cutting", "staying, left over", "need", "needed", "needing", "lead to", "leads to", "stiffness", "part", "parts", "later, after", "better, boss", "Consider removing this", "often", "workable", "since" |
